# Supplementary material for: Global discovery of human-infective RNA viruses: A modelling analysis
Source: PLoS Pathog. 2020 Nov 30;16(11):e1009079. doi: 10.1371/journal.ppat.1009079 (PMC7728385; doi:10.1371/journal.ppat.1009079)
Supplement: S1 Fig — The map was plotted with respect to transmissibility (top left: strictly zoonotic, top right: transmissible in humans), and transmission mode (bottom left: vector-borne viruses, bottom right: non-vector-borne viruses). In each subplot, the red spots indicate discovery points or centroids of polygons (administrative regions)–depending on the preciseness of the location provided by the original paper, with the size representing the cumulative virus species count. Centroid is the coordinate of the centre of mass in a spatial object. The red curve at the bottom left corner indicates the cumulative virus species discovery count over time. (PDF) [file ppat.1009079.s001.pdf]

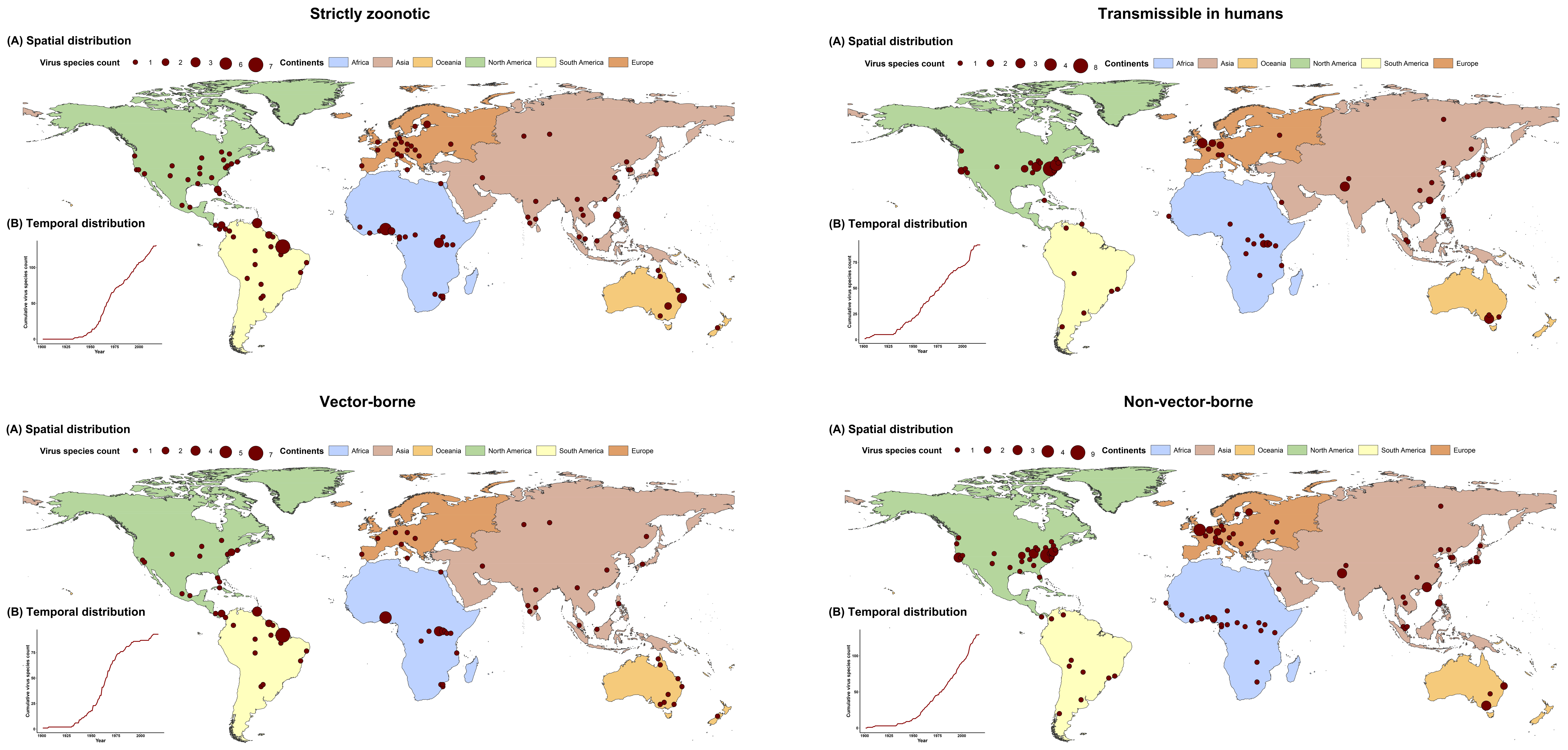

Strictly zoonotic
(A) Spatial distribution
Virus species count 1 2 3 6 7
Continents Africa Asia Oceania North America South America Europe
(B) Temporal distribution
Cumulative virus species count
0 50 100
1900 1925 1950 1975 2000
Year
Transmissible in humans
(A) Spatial distribution
Virus species count 1 2 3 4 8
Continents Africa Asia Oceania North America South America Europe
(B) Temporal distribution
Cumulative virus species count
0 25 50 75
1900 1925 1950 1975 2000
Year
Vector-borne
(A) Spatial distribution
Virus species count 1 2 4 5 7
Continents Africa Asia Oceania North America South America Europe
(B) Temporal distribution
Cumulative virus species count
0 25 50 75
1900 1925 1950 1975 2000
Year
Non-vector-borne
(A) Spatial distribution
Virus species count 1 2 3 4 9
Continents Africa Asia Oceania North America South America Europe
(B) Temporal distribution
Cumulative virus species count
0 50 100
1900 1925 1950 1975 2000
Year
