# Supplementary figures and images for: Global discovery of human-infective RNA viruses: A modelling analysis
Source: PLoS Pathog. 2020 Nov 30;16(11):e1009079. doi: 10.1371/journal.ppat.1009079 (PMC7728385; doi:10.1371/journal.ppat.1009079)

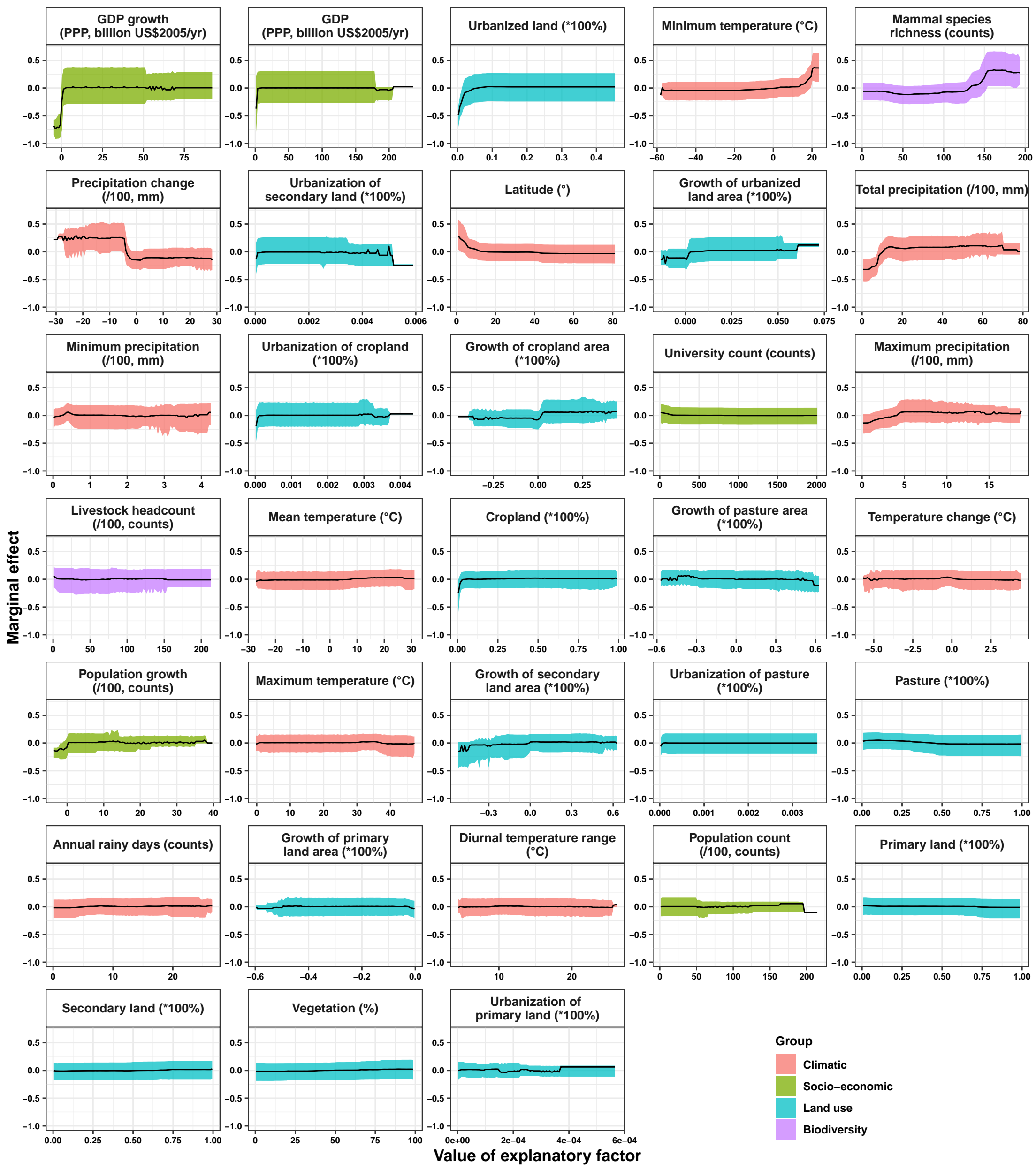

Supplement: S2 Fig — Partial dependence plots show the effect of an individual explanatory factor over its range on the response after factoring out other explanatory factors. Fitted lines represent the median (black) and 95% quantiles (coloured) based on 1000 replicated models. Y axes are centred around the mean without scaling. X axes show the range of sampled values of explanatory factors. (PDF) [file ppat.1009079.s002.pdf]

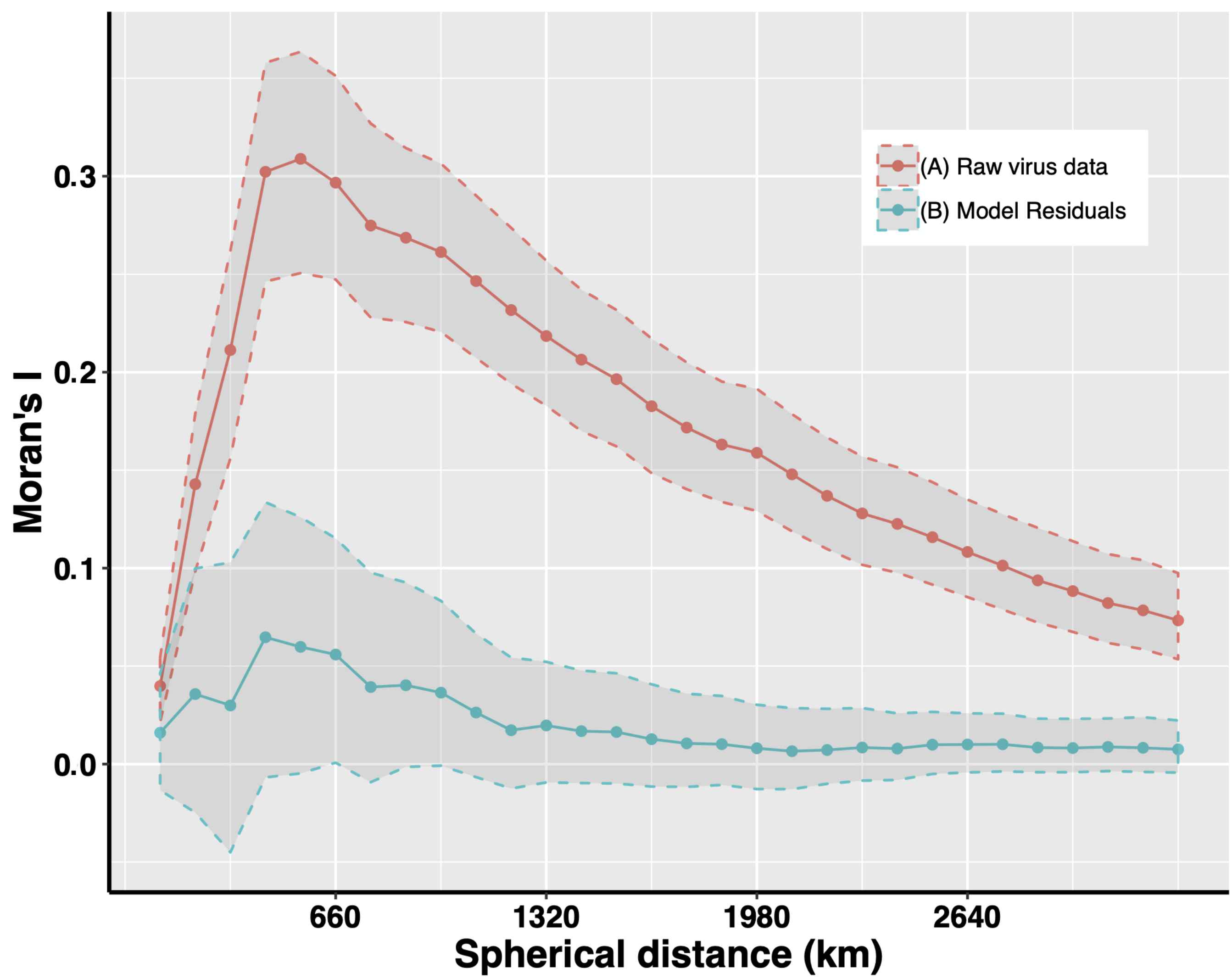

Supplement: S3 Fig — The solid line and dots represented the median Moran’s I value, and the grey area represented its 95% quantiles generated from 1000 samples (A: Raw virus data) or replicate BRT models (B: Model residuals). (PDF) [file ppat.1009079.s003.pdf]

(A) Strictly zoonotic

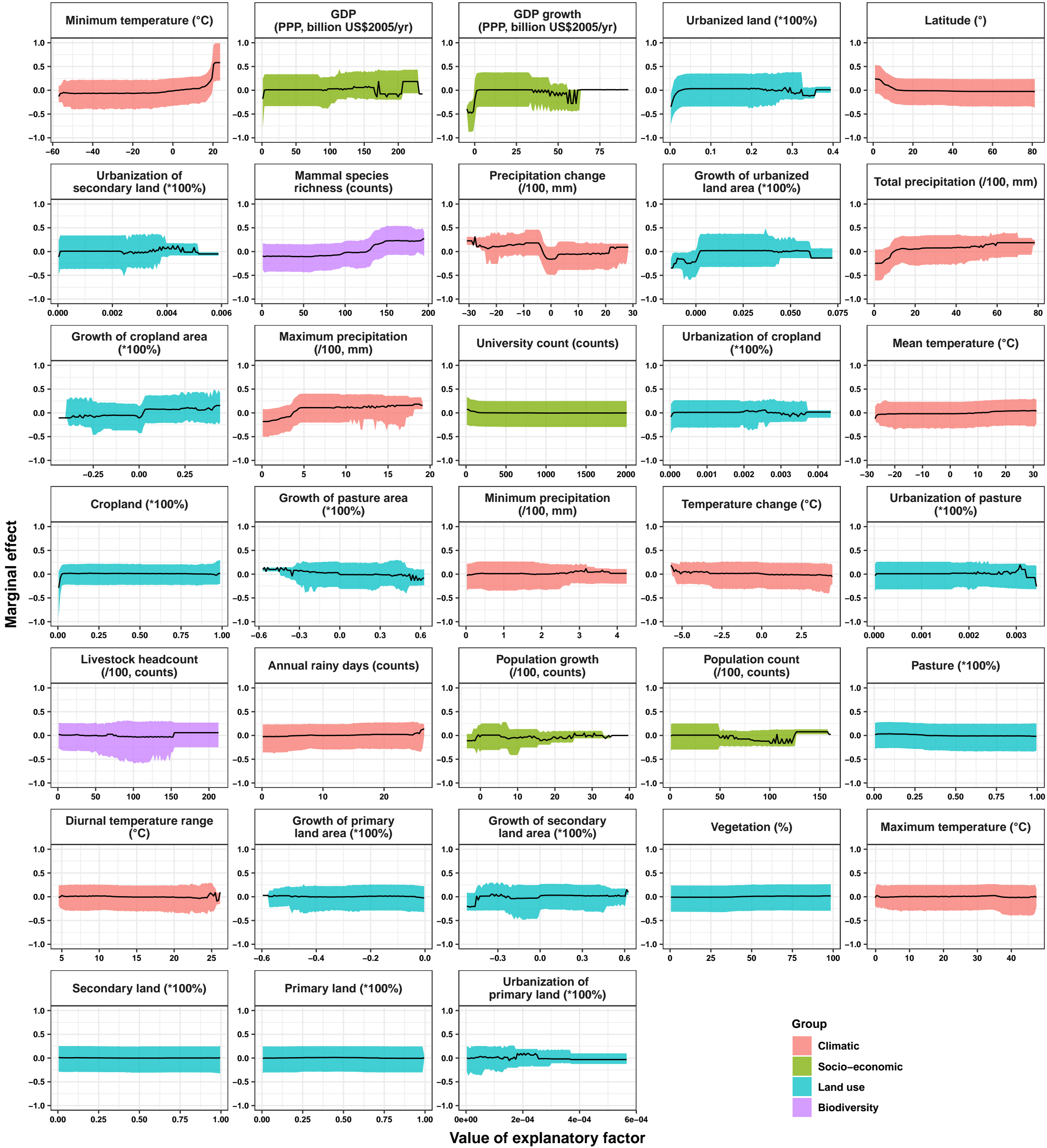

(B) Transmissible in humans

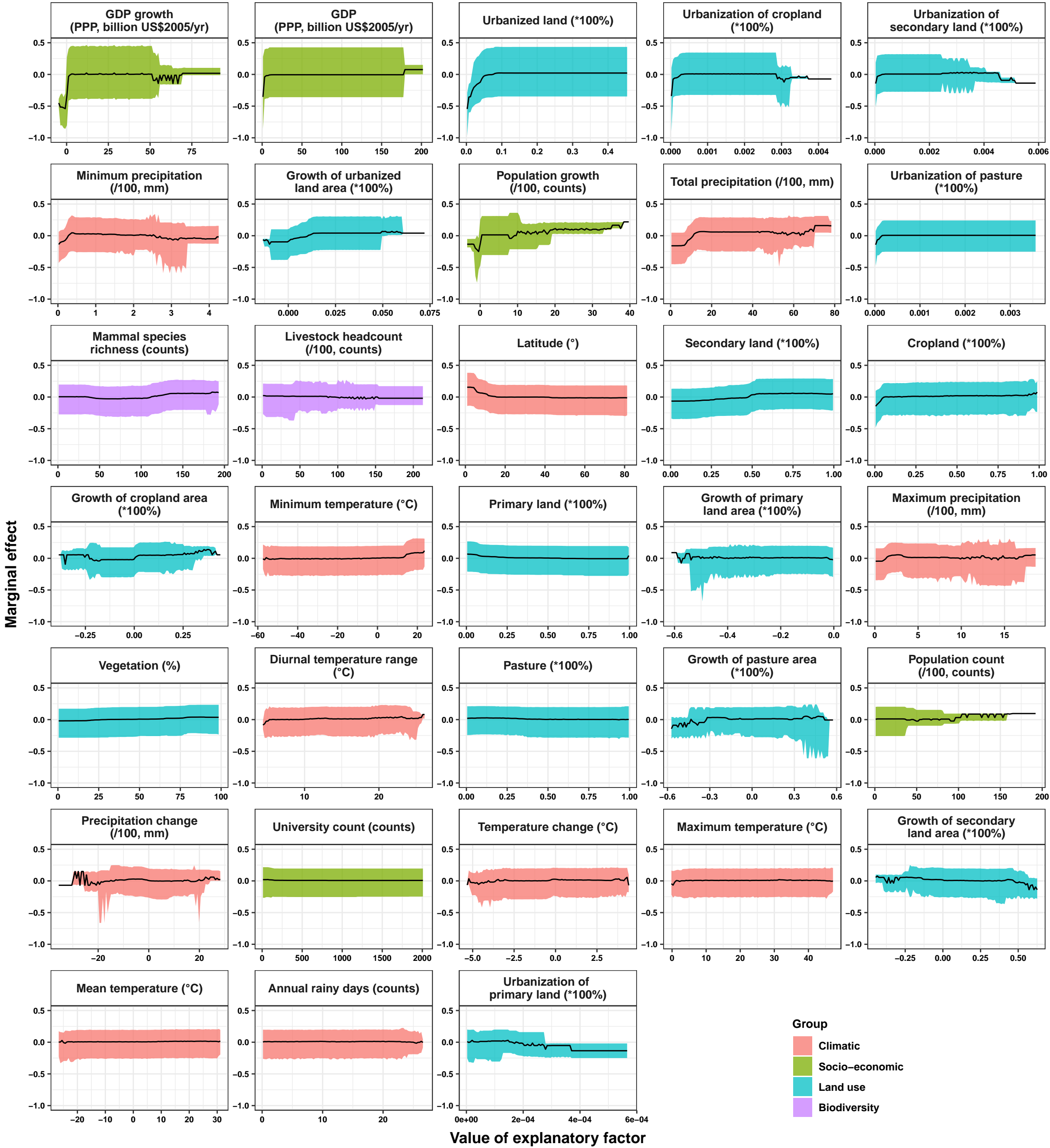

Supplement: S4 Fig — (A) Strictly zoonotic, (B) Transmissible in humans. Partial dependence plots show the effect of an individual explanatory factor over its range on the response after factoring out other explanatory factors. Fitted lines represent the median (black) and 95% quantiles (coloured) based on 1000 replicated models. Y axes are centred around the mean without scaling. X axes show the range of sampled values of explanatory factors. (PDF) [file ppat.1009079.s004.pdf]

(A) Vector-borne

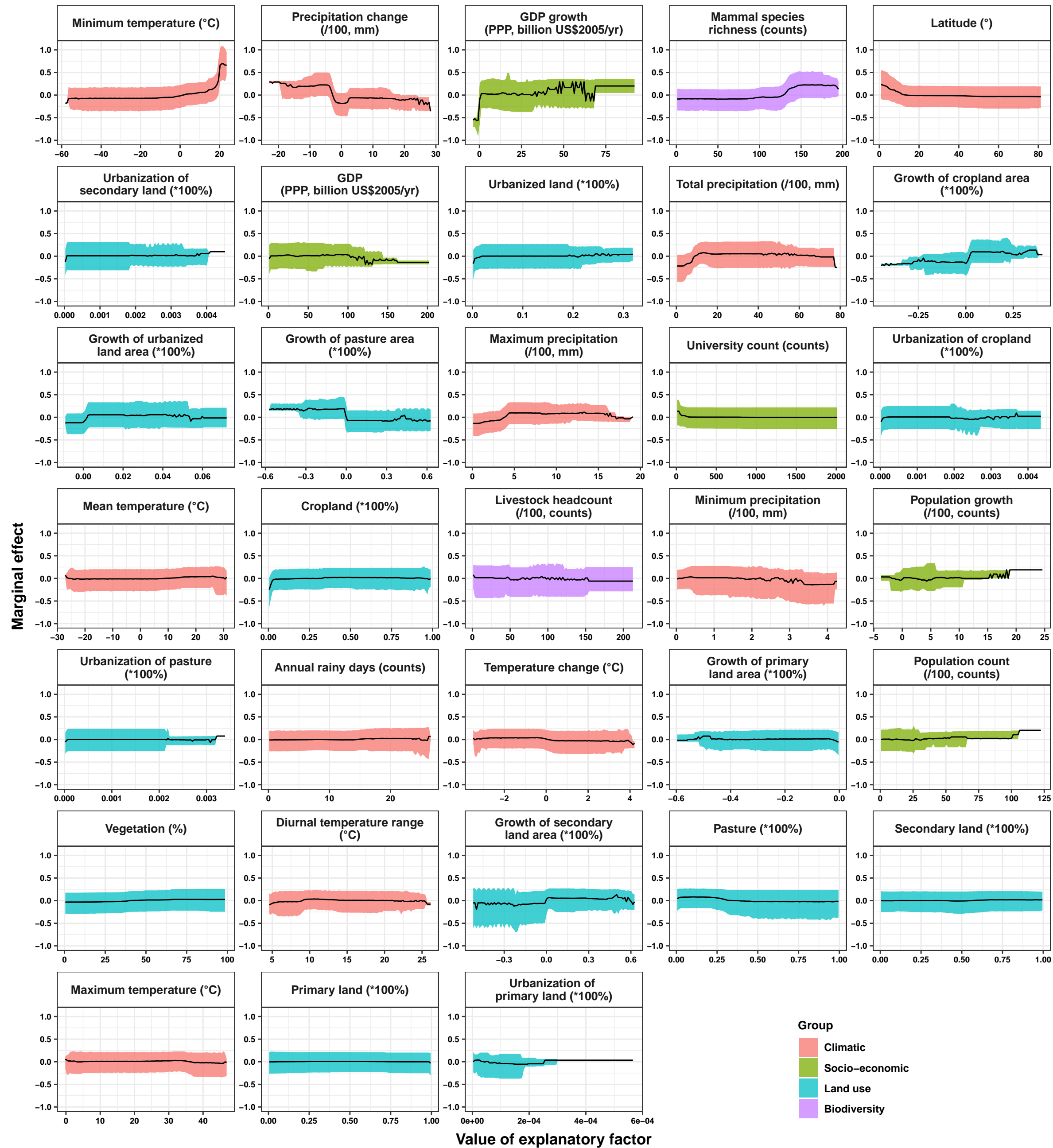

(B) Non-vector-borne

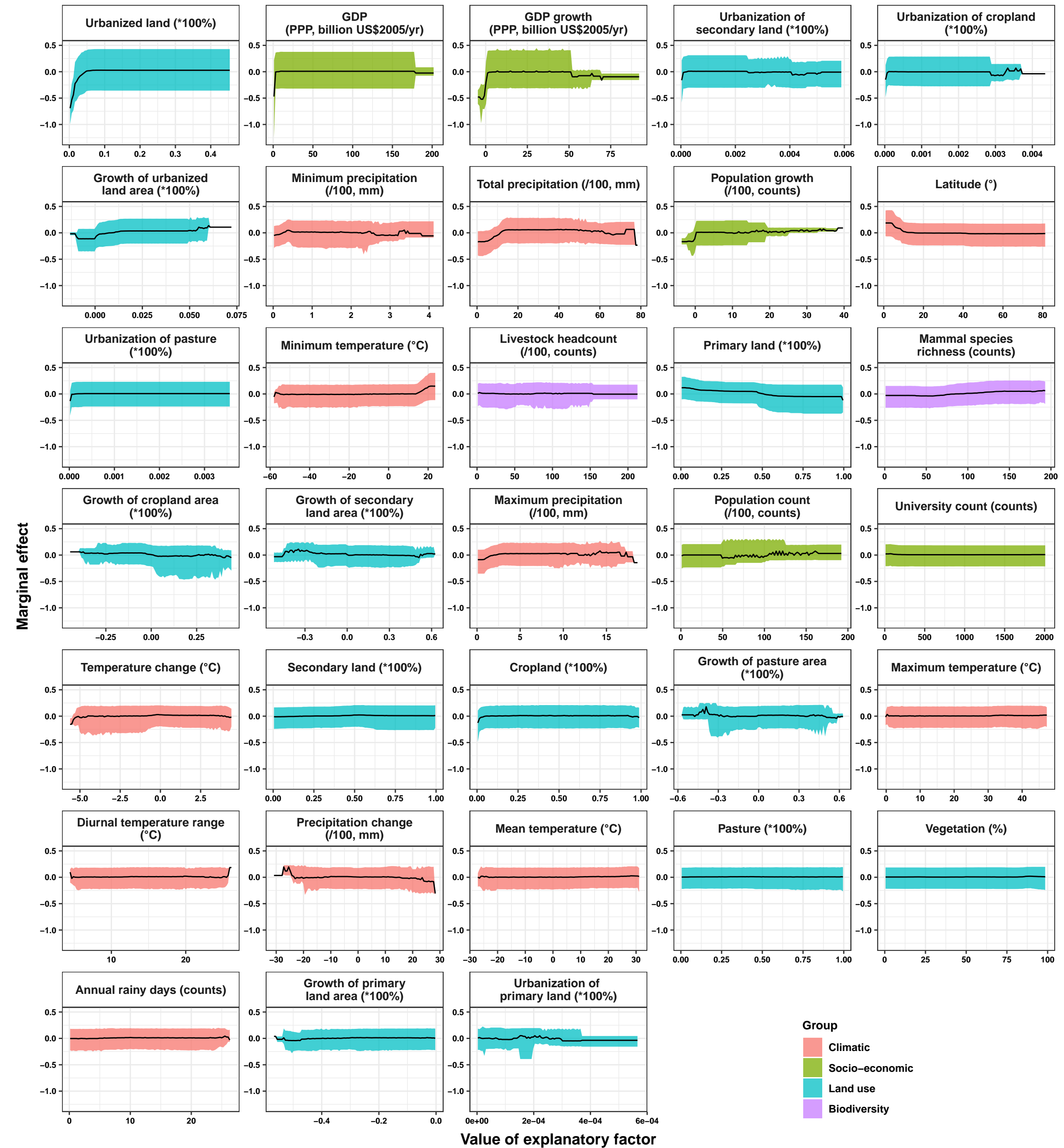

Supplement: S5 Fig — (A) Vector-borne, (B) Non-vector-borne. Partial dependence plots show the effect of an individual explanatory factor over its range on the response after factoring out other explanatory factors. Fitted lines represent the median (black) and 95% quantiles (coloured) based on 1000 replicated models. Y axes are centred around the mean without scaling. X axes show the range of sampled values of explanatory factors. (PDF) [file ppat.1009079.s005.pdf]

Strictly zoonotic

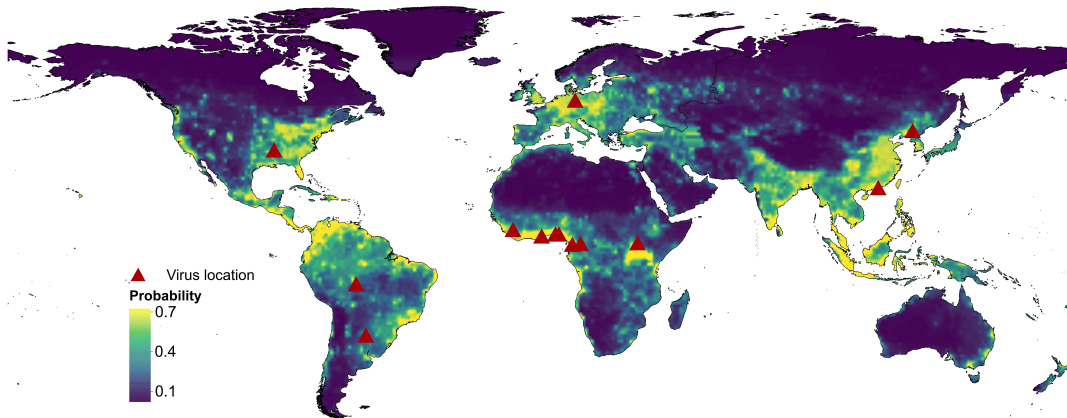

Transmissible in humans

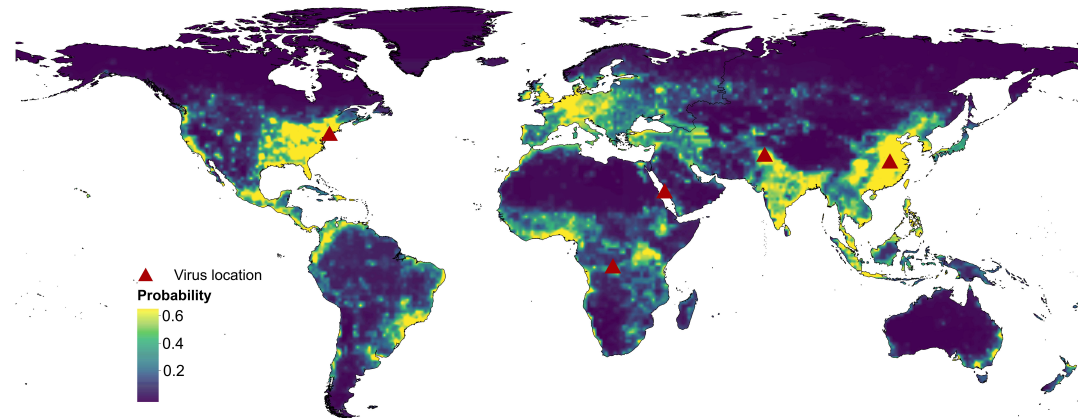

Vector-borne

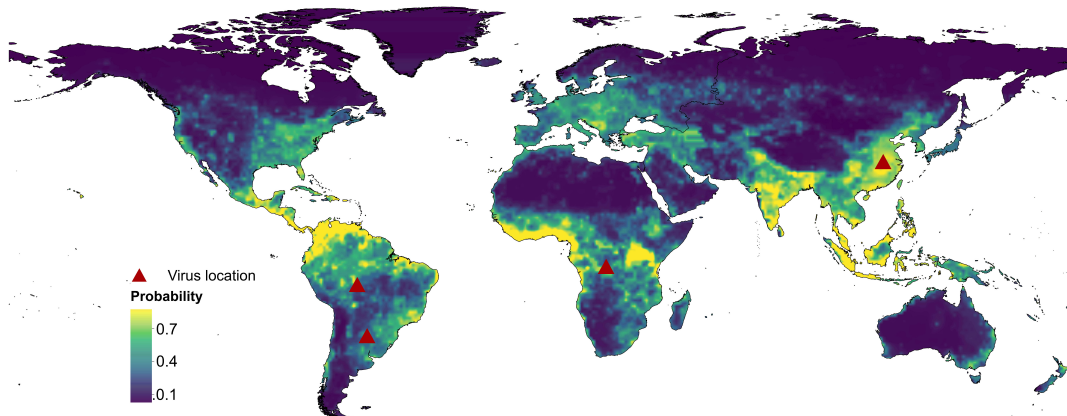

Non-vector-borne

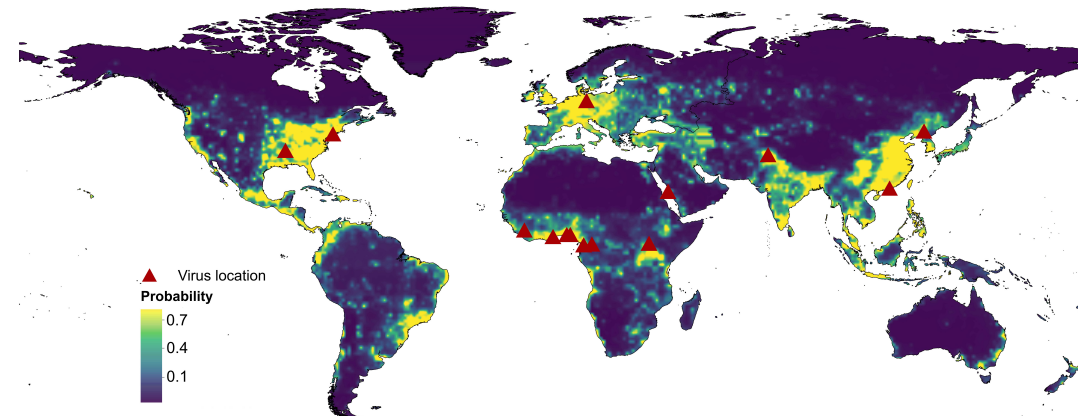

Supplement: S7 Fig — The triangles represented the actual discovery sites from 2010 to 2018, and the background colour represented the predicted discovery probability. (PDF) [file ppat.1009079.s007.pdf]

1901-1904

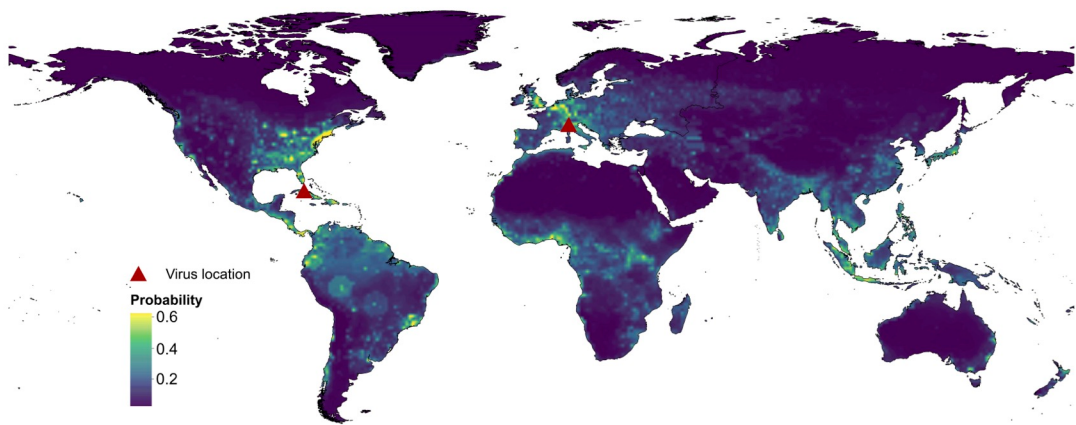

1905-1914

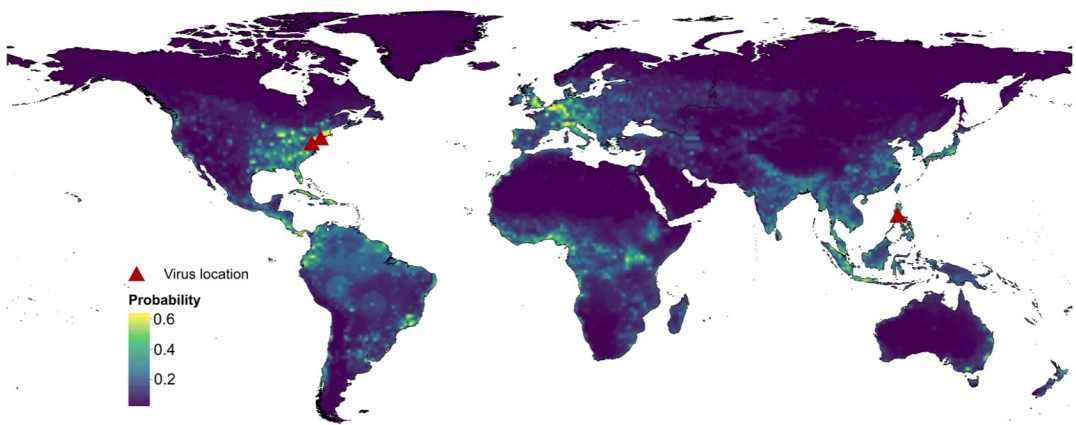

1915-1924

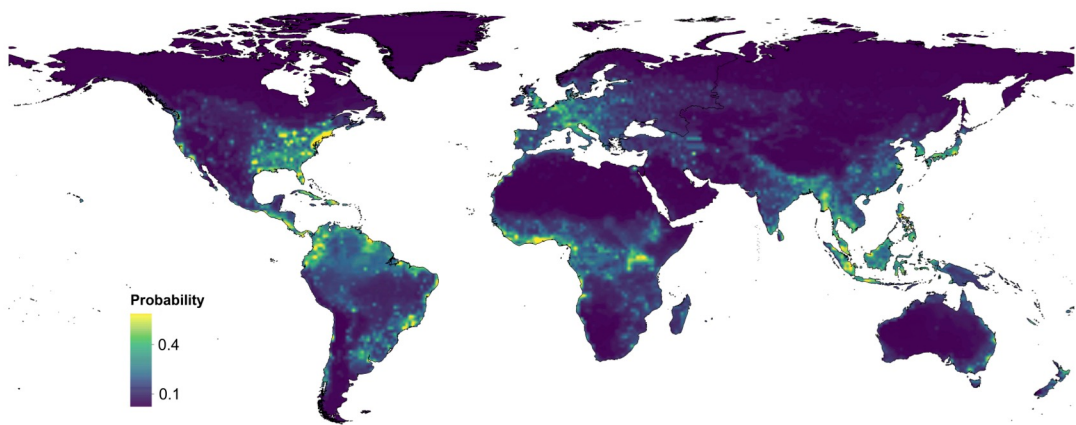

1925-1934

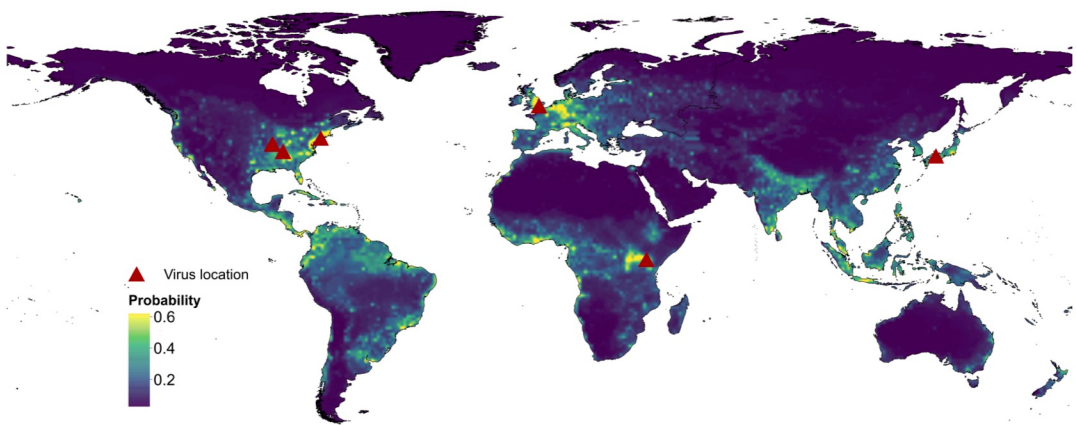

1935-1944

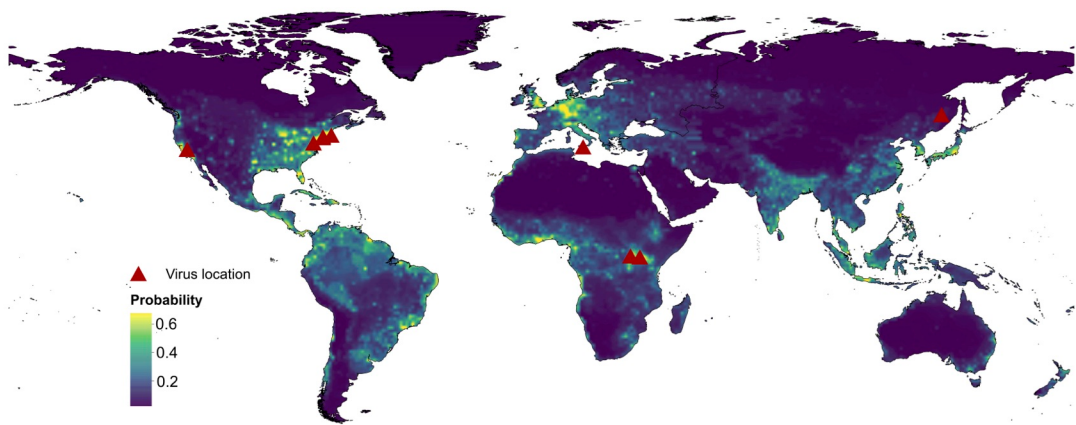

1945-1954

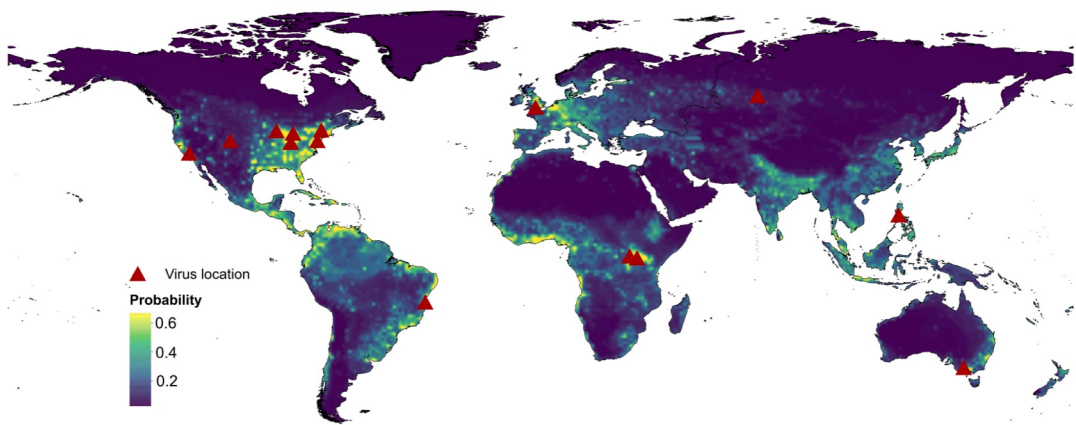

1955-1964

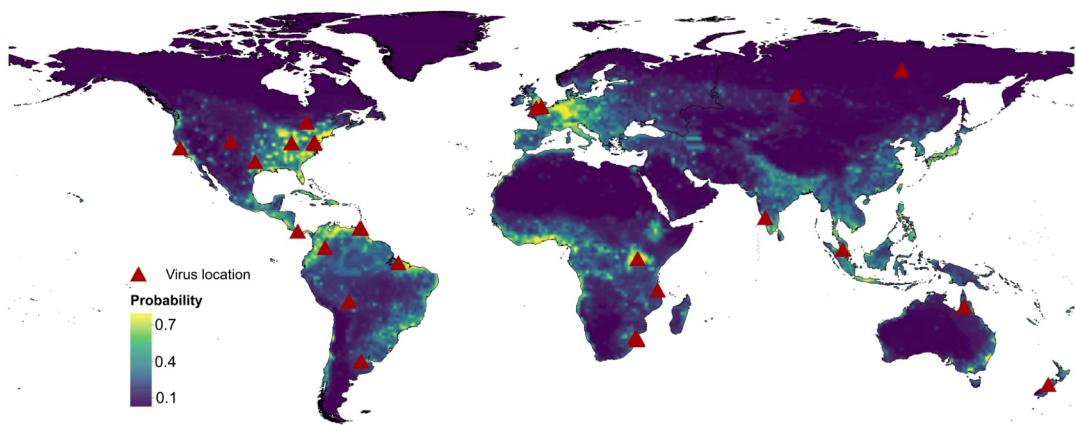

1965-1974

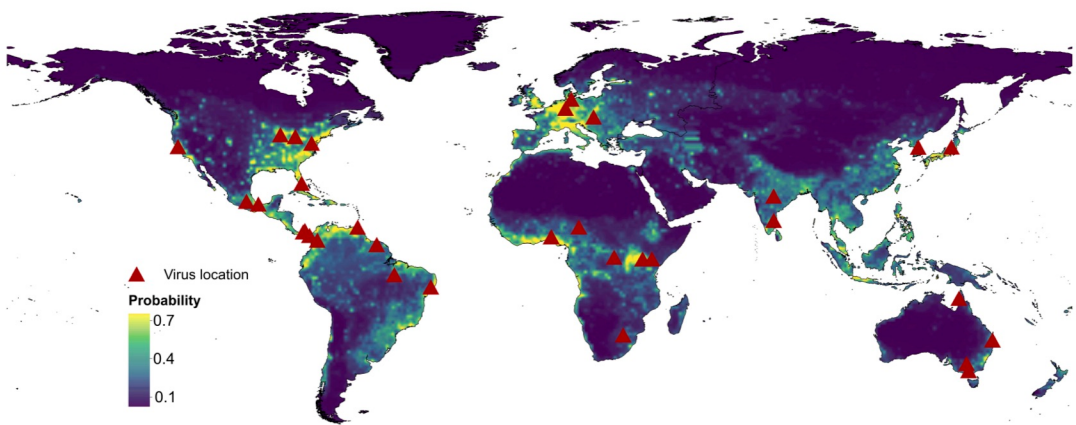

1975-1984

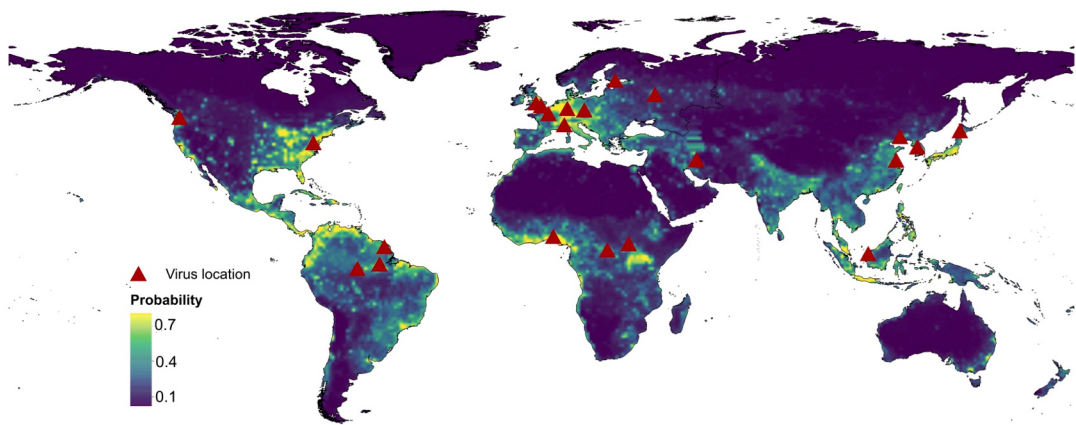

1985-1994

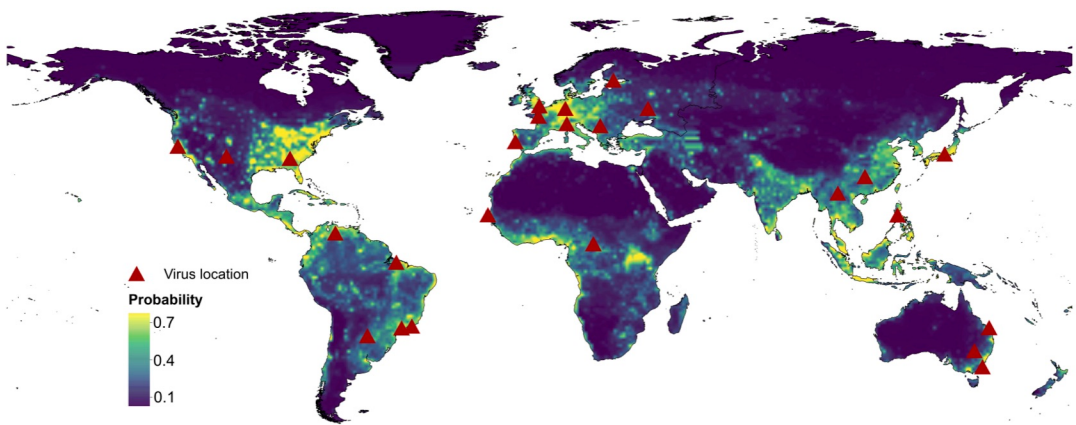

1995-2004

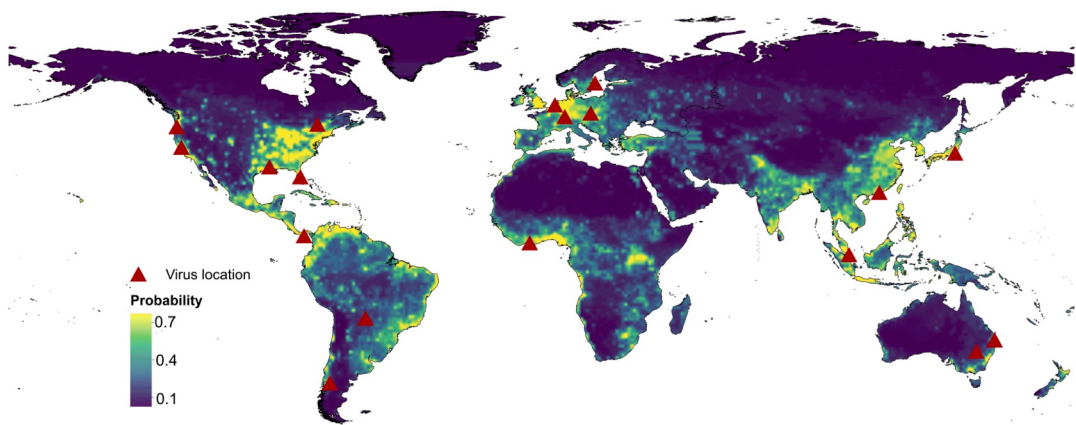

2005-2014

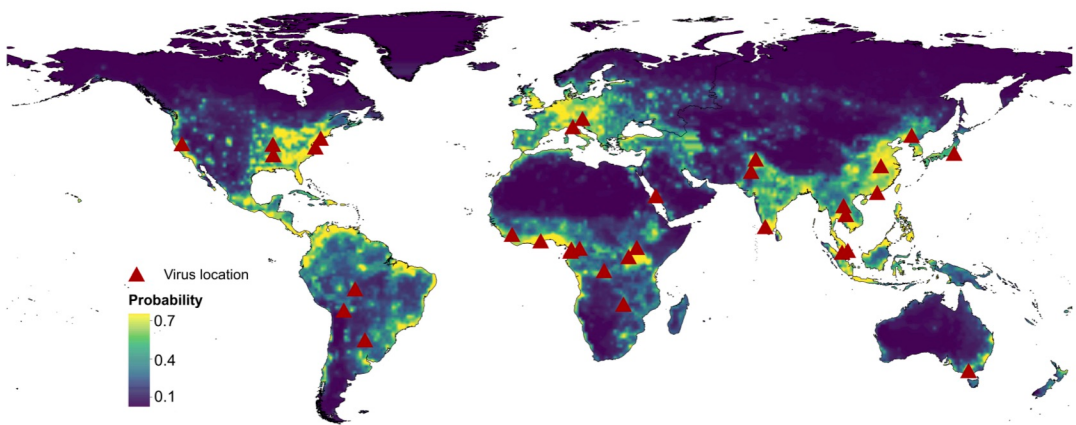

Supplement: S8 Fig — The triangles represented the actual discovery sites in each decade, and the background colour represented the predicted discovery probability. (PDF) [file ppat.1009079.s008.pdf]
