# Supplementary material for: Global discovery of human-infective RNA viruses: A modelling analysis
Source: PLoS Pathog. 2020 Nov 30;16(11):e1009079. doi: 10.1371/journal.ppat.1009079 (PMC7728385; doi:10.1371/journal.ppat.1009079)
Supplement: S6 Fig — The values of these explanatory variables and latitude in each grid cell were used to predict the virus discovery in the corresponding grid cell across the globe in 2010–2019. Explanatory variables were log transformed where necessary to get better visualization, not meaning they entered the model by logged values. (PDF) [file ppat.1009079.s006.pdf]

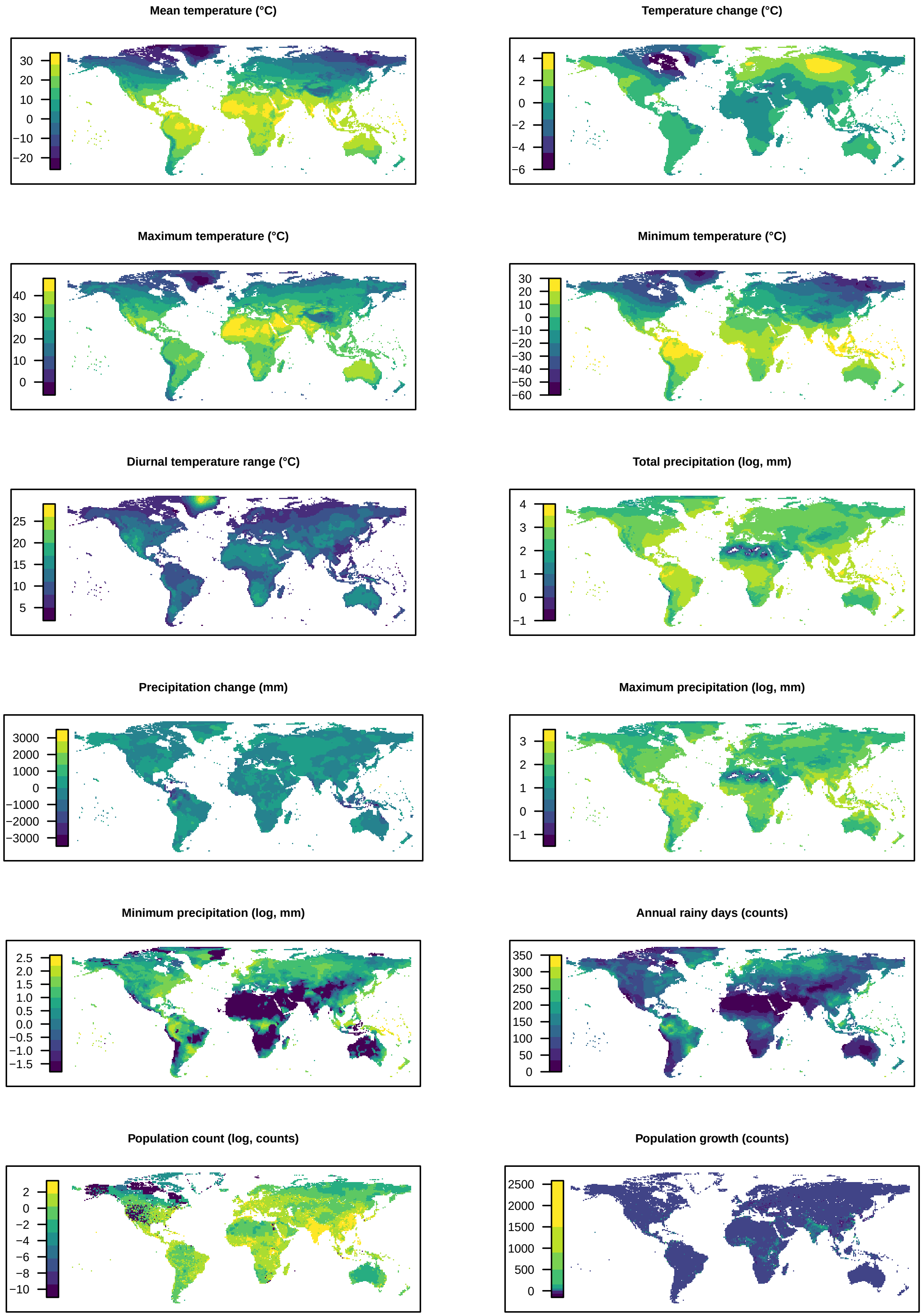
Mean temperature (°C)
30 20 10 0 −10 −20
Temperature change (°C)
4 2 0 −2 −4 −6
Maximum temperature (°C)
40 30 20 10 0
Minimum temperature (°C)
30 20 10 0 −10 −20 −30 −40 −50 −60
Diurnal temperature range (°C)
25 20 15 10 5
Total precipitation (log, mm)
4 3 2 1 0 −1
Precipitation change (mm)
3000 2000 1000 0 −1000 −2000 −3000
Maximum precipitation (log, mm)
3 2 1 0 −1
Minimum precipitation (log, mm)
2.5 2.0 1.5 1.0 0.5 0.0 −0.5 −1.0 −1.5
Annual rainy days (counts)
350 300 250 200 150 100 50 0
Population count (log, counts)
2 0 −2 −4 −6 −8 −10
Population growth (counts)
2500 2000 1500 1000 500 0

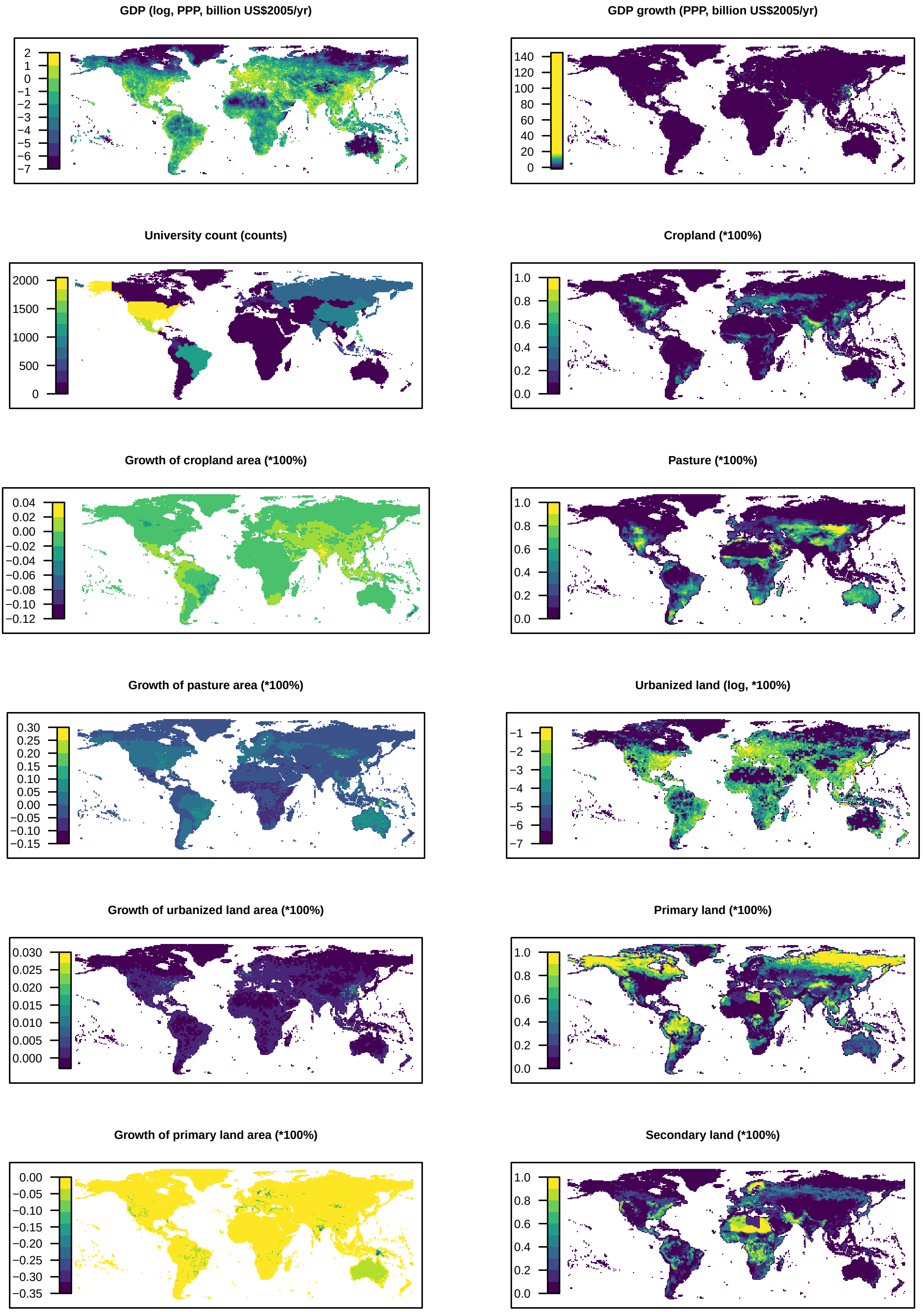
GDP (log, PPP, billion US$2005/yr)
2 1 0 −1 −2 −3 −4 −5 −6 −7
GDP growth (PPP, billion US$2005/yr)
140 120 100 80 60 40 20 0
University count (counts)
2000 1500 1000 500 0
Cropland (*100%)
1.0 0.8 0.6 0.4 0.2 0.0
Growth of cropland area (*100%)
0.04 0.02 0.00 −0.02 −0.04 −0.06 −0.08 −0.10 −0.12
Pasture (*100%)
1.0 0.8 0.6 0.4 0.2 0.0
Growth of pasture area (*100%)
0.30 0.25 0.20 0.15 0.10 0.05 0.00 −0.05 −0.10 −0.15
Urbanized land (log, *100%)
−1 −2 −3 −4 −5 −6 −7
Growth of urbanized land area (*100%)
0.030 0.025 0.020 0.015 0.010 0.005 0.000
Primary land (*100%)
1.0 0.8 0.6 0.4 0.2 0.0
Growth of primary land area (*100%)
0.00 −0.05 −0.10 −0.15 −0.20 −0.25 −0.30 −0.35
Secondary land (*100%)
1.0 0.8 0.6 0.4 0.2 0.0

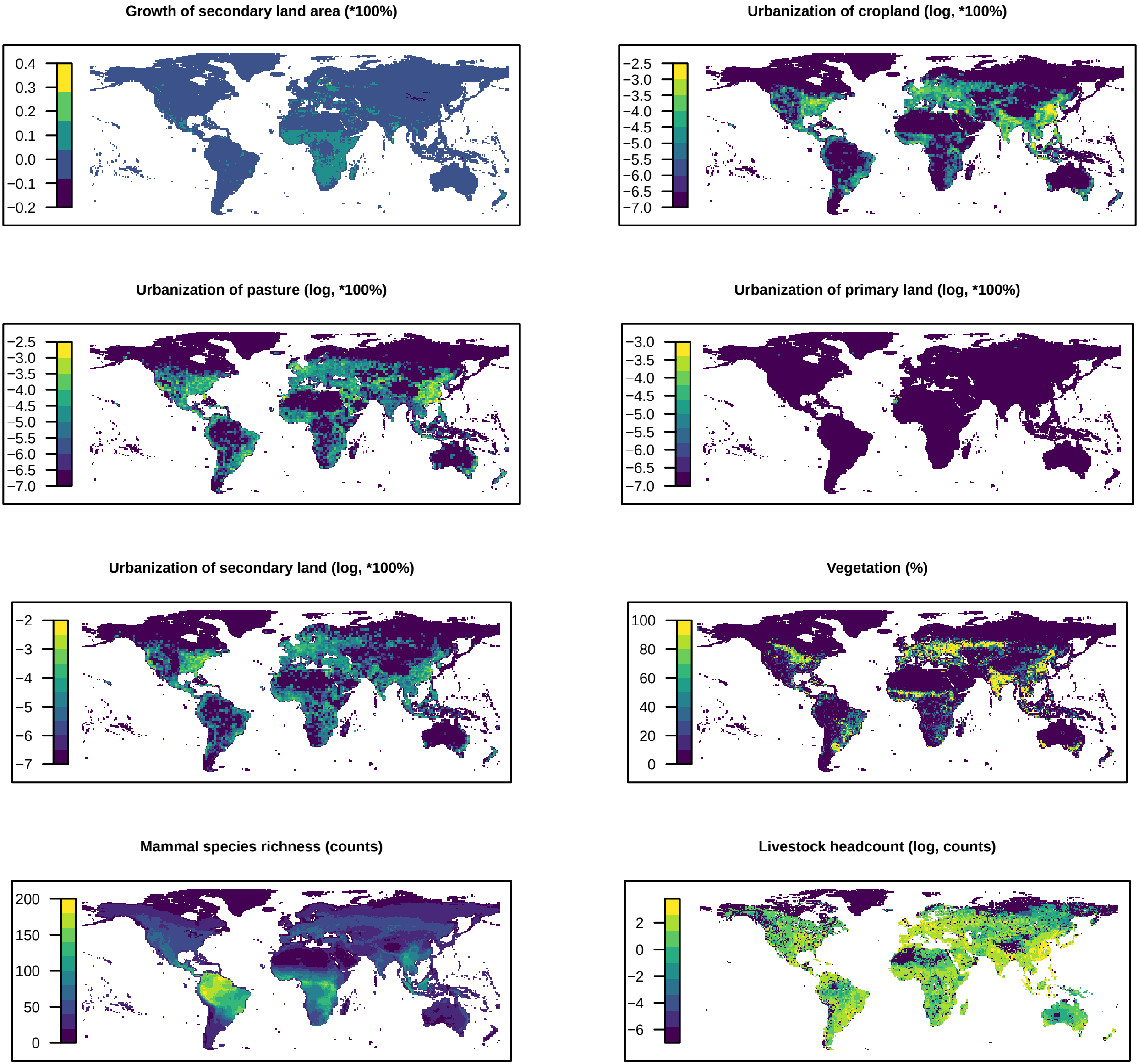

Growth of secondary land area (*100%)
0.4
0.3
0.2
0.1
0.0
−0.1
−0.2
Urbanization of cropland (log, *100%)
−2.5
−3.0
−3.5
−4.0
−4.5
−5.0
−5.5
−6.0
−6.5
−7.0
Urbanization of pasture (log, *100%)
−2.5
−3.0
−3.5
−4.0
−4.5
−5.0
−5.5
−6.0
−6.5
−7.0
Urbanization of primary land (log, *100%)
−3.0
−3.5
−4.0
−4.5
−5.0
−5.5
−6.0
−6.5
−7.0
Urbanization of secondary land (log, *100%)
−2
−3
−4
−5
−6
−7
Vegetation (%)
100
80
60
40
20
0
Mammal species richness (counts)
200
150
100
50
0
Livestock headcount (log, counts)
2
0
−2
−4
−6
