# Supplementary material for: Global discovery of human-infective RNA viruses: A modelling analysis
Source: PLoS Pathog. 2020 Nov 30;16(11):e1009079. doi: 10.1371/journal.ppat.1009079 (PMC7728385; doi:10.1371/journal.ppat.1009079)
Supplement: S1 Table — (DOCX) [file ppat.1009079.s009.docx]

## S1 Table Summary of the human RNA virus database

| **Species** | **Genus** | **Family** | **Year** | **Geographical information** | | | | | | **Transmission level** | **Vector-borne** |
| --- | --- | --- | --- | --- | --- | --- | --- | --- | --- | --- | --- |
|  |  |  |  | **Origin** | **Lat** | **Long** | **Type** | **Level** | **Location of patient** |  |  |
| *Argentinian mammarenavirus* ^1^ | *mammarenavirus* | *arena* | 1958 | Junín, Buenos Aires Province, Argentina | -34.59 | -60.95 | city | - | Y | Transmissible in humans | N |
| *Brazilian mammarenavirus* ^2^ | *mammarenavirus* | *arena* | 1994 | Jardim Sabiá Distrcit, São Paulo city, Brazil | -23.55 | -46.63 | district | - | Y | Transmissible in humans | N |
| *Cali mammarenavirus* ^3^ | *mammarenavirus* | *arena* | 1971 | Guatapé, Antioquia Department, Colombia | 6.23 | -75.16 | municipality | 3 | Y | Strictly zoonotic | N |
| *Chapare mammarenavirus* ^4^ | *mammarenavirus* | *arena* | 2008 | Samuzabeti town, Chapare Province, Bolivia | -17.41 | -66.17 | town | - | Y | Strictly zoonotic | N |
| *Guanarito mammarenavirus* ^5^ | *mammarenavirus* | *arena* | 1991 | Guanarito, Portuguesa State, Venezuela | 8.51 | -69.00 | city | 3 | Y | Transmissible in humans | N |
| *Lassa mammarenavirus* ^6^ | *mammarenavirus* | *arena* | 1970 | Lassa, Borno State, Nigeria | 10.69 | 13.27 | town | - | Y | Transmissible in humans | N |
| *Lujo mammarenavirus* ^7^ | *mammarenavirus* | *arena* | 2009 | Lusaka city, Lusaka District, Zambia | -15.39 | 28.32 | city | 3 | Y | Transmissible in humans | N |
| *Lymphocytic choriomeningitis mammarenavirus* ^8^ | *mammarenavirus* | *arena* | 1934 | St. Louis county, Missouri, USA | 38.73 | -90.38 | county | 3 | Y | Transmissible in humans^†^ | N |
| *Machupo mammarenavirus* ^9^ | *mammarenavirus* | *arena* | 1964 | Beni Department, Bolivia | -14.38 | -65.1 | department | 2 | Y | Transmissible in humans | N |
| *Mobala mammarenavirus* ^10^ | *mammarenavirus* | *arena* | 1985 | Bouboui & Gomoka village, Boali town, Central Africa | 4.89 | 18.14 | village | - | Y | Strictly zoonotic | N |
| *Whitewater Arroyo mammarenavirus* ^11^ | *mammarenavirus* | *arena* | 2000 | Alameda County, California, USA | 37.6 | -121.72 | county | 3 | Y | Strictly zoonotic | N |
| *Mamastrovirus 1* ^12^ | *mamastrovirus* | *astro* | 1975 | London, UK | 51.51 | -0.13 | city | 3 | N | Transmissible in humans | N |
| *Mamastrovirus 6* ^13^ | *mamastrovirus* | *astro* | 2008 | Melbourne, Victoria, Australia | -37.81 | 144.96 | city | - | Y | Transmissible in humans | N |
| *Mamastrovirus 8* ^14^ | *mamastrovirus* | *astro* | 2009 | St. Louis city, USA | 38.63 | -90.2 | city | - | Y | Transmissible in humans | N |
| *Mamastrovirus 9* ^15^ | *mamastrovirus* | *astro* | 2009 | Accomack and Northampton Counties, Virginia, USA | 37.71 | -75.81 | county | 3 | Y | Transmissible in humans | N |
| *Mammalian 1 orthobornavirus* ^16^ | *orthobornavirus* | *borna* | 1985 | Gießen, Gießen District, Hesse State, Germany | 50.58 | 8.68 | town | - | Y | Strictly zoonotic | N |
| *Mammalian 2 orthobornavirus* ^17^ | *orthobornavirus* | *borna* | 2015 | Saxony-Anhalt State, Germany | 51.95 | 11.69 | state | 2 | Y | Strictly zoonotic | N |
| *Norwalk virus* ^18^ | *norovirus* | *calici* | 1972 | Norwalk, Ohio, USA | 41.24 | -82.62 | city | - | Y | Transmissible in humans | N |
| *Sapporo virus* ^19^ | *sapovirus* | *calici* | 1980 | Sapporo, Hokkaido Prefecture, Japan | 43.06 | 141.35 | city | 3 | Y | Transmissible in humans | N |
| *Vesicular exanthema of swine virus* ^20^ | *vesivirus* | *calici* | 1998 | Corvallis, Oregon, USA | 44.56 | -123.26 | city | - | N | Strictly zoonotic | N |
| *Alphacoronavirus 1* ^21^ | *alphacoronavirus* | *corona* | 2007 | Chiba City, Chiba Prefecture, Japan | 35.61 | 140.11 | city | 3 | Y | Strictly zoonotic | N |
| *Human coronavirus 229E* ^22^ | *alphacoronavirus* | *corona* | 1966 | Chicago, USA | 41.88 | -87.63 | city | - | Y | Transmissible in humans | N |
| *Human coronavirus NL63* ^23^ | *alphacoronavirus* | *corona* | 2004 | Rotterdam, Netherlands | 51.92 | 4.48 | city | 3 | Y | Transmissible in humans | N |
| *Betacoronavirus 1* ^24^ | *betacoronavirus* | *corona* | 1967 | Bethesda, Montgomery County, Maryland, USA | 38.98 | -77.09 | CDP | - | Y | Transmissible in humans | N |
| *Human coronavirus HKU1* ^25^ | *betacoronavirus* | *corona* | 2005 | Hong Kong, China | 22.4 | 114.11 | city | 2 | Y | Transmissible in humans | N |
| *Middle East respiratory syndrome-related coronavirus* ^26^ | *betacoronavirus* | *corona* | 2012 | Jeddah, Makkah region, Saudi Arabia | 21.29 | 39.24 | city | - | Y | Transmissible in humans | N |
| *Severe acute respiratory syndrome-related coronavirus* ^27^ | *betacoronavirus* | *corona* | 2003 | Hong Kong, China | 22.4 | 114.11 | city | 2 | Y | Transmissible in humans | N |
| *Human torovirus* ^28^ | *torovirus* | *corona* | 1984 | Birmingham, UK | 52.49 | -1.89 | city | - | Y | Transmissible in humans | N |
| *Bundibugyo ebolavirus* ^29^ | *ebolavirus* | *filo* | 2008 | Bundibugyo & Kikyo town, Bwamba county, Uganda | 0.71 | 30.06 | town | - | Y | Transmissible in humans | N |
| *Reston ebolavirus* ^30^ | *ebolavirus* | *filo* | 1991 | Manila, Philippines | 14.6 | 120.98 | city | 3 | Y | Strictly zoonotic | N |
| *Sudan ebolavirus* ^31^ | *ebolavirus* | *filo* | 1977 | Maridi Town, Maridi County, South Sudan | 4.91 | 29.45 | town | - | Y | Transmissible in humans | N |
| *Tai Forest ebolavirus* ^32^ | *ebolavirus* | *filo* | 1995 | Abidjan, Abidjan department, Côte d'Ivoire | 5.36 | -4.01 | city | - | Y | Strictly zoonotic | N |
| *Zaire ebolavirus* ^33^ | *ebolavirus* | *filo* | 1977 | Yambuku village, DRC | 2.83 | 22.22 | village | - | Y | Transmissible in humans | N |
| *Marburg Marburgvirus* ^34^ | *marburgvirus* | *filo* | 1968 | Marburg, Germany | 50.8 | 8.77 | town | - | Y | Transmissible in humans | N |
| *Aroa virus* ^35^ | *flavivirus* | *flavi* | 1971 | La Arenosa village, Panama City, Panama | 9.04 | -79.95 | village | - | Y | Strictly zoonotic | Y |
| *Bagaza virus* ^36^ | *flavivirus* | *flavi* | 2009 | Kerala state, India | 10.85 | 76.27 | state | 2 | Y | Strictly zoonotic | Y |
| *Banzi virus* ^37^ | *flavivirus* | *flavi* | 1959 | Maponde's Kraal (Usutu river), South Africa | -26.52 | 31.67 | village | - | Y | Strictly zoonotic | Y |
| *Cacipacore virus* ^38^ | *flavivirus* | *flavi* | 2011 | Theobroma city, State of Rondônia, Brazil | -10.16 | -62.36 | city | 3 | Y | Strictly zoonotic | Y |
| *Dengue virus* ^39^ | *flavivirus* | *flavi* | 1907 | Fort William McKinley, Philippines (now Fort Bonifacio, located in Taguig City, Metro Manila, Philippines) | 14.56 | 121.07 | area | - | Y | Transmissible in humans | Y |
| *Edge Hill virus* ^40^ | *flavivirus* | *flavi* | 1985 | New South Wales, Australia | -31.25 | 146.92 | state | 2 | Y | Strictly zoonotic | Y |
| *Gadgets Gully virus* ^41^ | *flavivirus* | *flavi* | 1991 | Heron Island, Great Barrier reef, Australia | -23.44 | 151.91 | area | - | Y | Strictly zoonotic | Y |
| *Ilheus virus* ^42^ | *flavivirus* | *flavi* | 1947 | Ilheus city, State of Bahia, Brazil | -14.79 | -39.05 | city | 3 | Y | Strictly zoonotic | Y |
| *Japanese encephalitis virus* ^43^ | *flavivirus* | *flavi* | 1935 | Okayama, Okayama Prefecture, Japan | 34.66 | 133.92 | city | 3 | Y | Transmissible in humans^†^ | Y |
| *Kokobera virus* ^40^ | *flavivirus* | *flavi* | 1964 | Mitchell River, Australia; Lockhart River, Australia (Cape York Peninsula) | -16.17 | 142.84 | area | - | Y | Strictly zoonotic | Y |
| *Kyasanur forest disease virus* ^44^ | *flavivirus* | *flavi* | 1957 | Shimoga District, State of Karnataka, India | 13.93 | 75.57 | district | 3 | Y | Strictly zoonotic | Y |
| *Langat virus* ^45^ | *flavivirus* | *flavi* | 1956 | Kuala Lumpur, Malaysia | 3.14 | 101.69 | city | 2 | Y | Strictly zoonotic | Y |
| *Louping ill virus* ^46^ | *flavivirus* | *flavi* | 1934 | New York, New York State, USA | 40.71 | -74.01 | city | - | Y | Strictly zoonotic | Y |
| *Murray Valley encephalitis virus* ^47^ | *flavivirus* | *flavi* | 1952 | Murray Valley, Northern Victoria, Australia | -36.9 | 142.96 | area | - | Y | Strictly zoonotic | Y |
| *Ntaya virus* ^48^ | *flavivirus* | *flavi* | 1952 | Bwamba county, Uganda | 0.75 | 30.02 | county | 3 | Y | Strictly zoonotic | Y |
| *Omsk hemorrhagic fever virus* ^49^ | *flavivirus* | *flavi* | 1948 | Omsk Oblast, Russia | 54.99 | 73.32 | federal subject | 2 | Y | Strictly zoonotic | Y |
| *Powassan virus* ^50^ | *flavivirus* | *flavi* | 1959 | Powassan, Ontario, Canada | 46.08 | -79.37 | municipality | - | Y | Strictly zoonotic | Y |
| *Rio Bravo virus* ^51^ | *flavivirus* | *flavi* | 1962 | Dallas city, Texas, USA | 32.78 | -96.8 | city | - | Y | Strictly zoonotic | N |
| *Saint Louis encephalitis virus* ^52^ | *flavivirus* | *flavi* | 1933 | St. Louis City, USA | 38.63 | -90.2 | city | - | Y | Strictly zoonotic | Y |
| *Tembusu virus* ^53^ | *flavivirus* | *flavi* | 1975 | Kampong Tijirak village, Kuching Town, Malaysia | 1.55 | 110.36 | village | - | Y | Strictly zoonotic | Y |
| *Tick-borne encephalitis virus* ^54^ | *flavivirus* | *flavi* | 1938 | Khabarovsk Krai, Russia | 48.52 | 135.1 | federal subject | 2 | Y | Transmissible in humans ^†^ | Y |
| *Uganda S virus* ^55^ | *flavivirus* | *flavi* | 1952 | Bwamba county, Bundibugyo District, Uganda | 0.75 | 30.02 | county | 3 | Y | Strictly zoonotic | Y |
| *Usutu virus* ^56^ | *flavivirus* | *flavi* | 2009 | Emilia Romagna region, Italy | 44.6 | 11.22 | region | 2 | Y | Strictly zoonotic | Y |
| *Wesselsbron virus* ^57^ | *flavivirus* | *flavi* | 1957 | Lake Simbu region, KwaZulu-Natal Province, South Africa | -27.36 | 32.32 | area | - | Y | Strictly zoonotic | Y |
| *West Nile virus* ^58^ | *flavivirus* | *flavi* | 1940 | Omogo, West Nile district, Uganda | 0.42 | 33.21 | village | - | Y | Transmissible in humans^†^ | Y |
| *Yellow fever virus* ^59^ | *flavivirus* | *flavi* | 1901 | Quemados town, Cuba | 22.79 | -80.25 | town | - | N* | Transmissible in humans | Y |
| *Zika virus* ^60^ | *flavivirus* | *flavi* | 1952 | Zika forest, Uganda | 0.12 | 32.53 | area | - | Y | Transmissible in humans | Y |
| *Hepacivirus C* ^61^ | *hepacivirus* | *flavi* | 1989 | Emeryville, California, USA | 37.83 | -122.29 | city | - | N | Transmissible in humans | N |
| *Pegivirus C* ^62^ | *pegivirus* | *flavi* | 1995 | Yokohama, Kanagawa Prefecture, Japan | 35.69 | 139.69 | city | 3 | Y | Transmissible in humans | N |
| *Pegivirus H* ^63^ | *pegivirus* | *flavi* | 2015 | New York, New York State, USA | 40.71 | -74.01 | city | - | Y | Transmissible in humans | N |
| *Pestivirus A* ^64^ | *pestivirus* | *flavi* | 1988 | Milan, Province of Milan, Lombardy region, Italy | 45.46 | 9.19 | city | - | Y | Strictly zoonotic | N |
| *Andes orthohantavirus* ^65^ | *orthohantavirus* | *hanta* | 1996 | El Bolsón, Río Negro Province, Argentina | -41.96 | -71.54 | town | - | Y | Transmissible in humans | N |
| *Bayou orthohantavirus* ^66^ | *orthohantavirus* | *hanta* | 1995 | Louisiana state, USA | 30.98 | -91.96 | state | 2 | Y | Strictly zoonotic | N |
| *Black creek canal orthohantavirus* ^67^ | *orthohantavirus* | *hanta* | 1995 | Miami-Dade County, Florida, USA | 25.76 | -80.33 | county | 3 | Y | Strictly zoonotic | N |
| *Choclo orthohantavirus* ^68^ | *orthohantavirus* | *hanta* | 2000 | Las Tablas Town, Las Tablas District, Panama | 7.77 | -80.27 | town | - | Y | Strictly zoonotic | N |
| *Dobrava-Belgrade orthohantavirus* ^69^ | *orthohantavirus* | *hanta* | 1992 | Belgrade, Yugoslavia (now Belgrade, Serbia) | 44.79 | 20.45 | city | 2 | Y | Strictly zoonotic | N |
| *Hantaan orthohantavirus* ^70^ | *orthohantavirus* | *hanta* | 1978 | Seoul, Korea | 37.57 | 126.98 | city | 2 | Y | Strictly zoonotic | N |
| *Laguna Negra orthohantavirus* ^71^ | *orthohantavirus* | *hanta* | 1997 | Chaco region, Paraguay | -20.09 | -59.47 | region | 2 | Y | Strictly zoonotic | N |
| *Puumala orthohantavirus* ^72^ | *orthohantavirus* | *hanta* | 1980 | Helsinki, Uusimaa region, Finland | 60.17 | 24.94 | city | - | Y | Strictly zoonotic | N |
| *Sangassou orthohantavirus* ^73^ | *orthohantavirus* | *hanta* | 2010 | Sangassou village, Macenta district, Forest Guinea | 8.24 | -9.32 | village | - | Y | Strictly zoonotic | N |
| *Seoul orthohantavirus* ^74^ | *orthohantavirus* | *hanta* | 1982 | Jiangsu, China | 33.14 | 119.79 | province | 2 | Y | Strictly zoonotic | N |
| *Sin Nombre orthohantavirus* ^75^ | *orthohantavirus* | *hanta* | 1993 | New Mexico, USA | 34.52 | -105.87 | state | 2 | Y | Strictly zoonotic | N |
| *Thailand orthohantavirus* ^76^ | *orthohantavirus* | *hanta* | 2006 | Surin province, Thailand | 14.88 | 103.49 | province | 2 | Y | Strictly zoonotic | N |
| *Thottapalayam orthohantavirus* ^77^ | *orthohantavirus* | *hanta* | 2007 | Nong khai Province, Thailand | 17.88 | 102.74 | province | 2 | Y | Strictly zoonotic | N |
| *Tula orthohantavirus* ^78^ | *orthohantavirus* | *hanta* | 1996 | Moravia, Czech Republic | 49.2 | 16.61 | region | 2 | Y | Strictly zoonotic | N |
| *Orthohepevirus A* ^79^ | *orthohepevirus* | *hepe* | 1983 | Moscow Oblast, Russia (now Moscow city) | 55.34 | 38.29 | federal subject | 2 | N | Transmissible in humans | N |
| *Orthohepevirus C* ^80^ | *orthohepevirus* | *hepe* | 2018 | Hong Kong, China | 22.4 | 114.11 | city | 2 | Y | Strictly zoonotic | N |
| *Crimean-Congo haemorrhagic fever orthonairovirus* ^81^ | *orthonairovirus* | *nairo* | 1967 | Kisangani, Tshopo province, DRC | 0.53 | 25.19 | city | - | Y | Transmissible in humans | N |
| *Dugbe orthonairovirus* ^82^ | *orthonairovirus* | *nairo* | 1969 | Ibadan, Nigeria | 7.35 | 3.88 | city | - | Y | Strictly zoonotic | Y |
| *Nairobi sheep disease orthonairovirus* ^83^ | *orthonairovirus* | *nairo* | 1969 | Vellore, Vellore District, Tamil Nadu state, India | 12.92 | 79.13 | city | - | Y | Strictly zoonotic | Y |
| *Thiafora orthonairovirus* ^84^ | *orthonairovirus* | *nairo* | 1989 | Saulges village, Western France | 47.98 | -0.41 | village | - | Y | Strictly zoonotic | Y |
| *Influenza A virus* ^85^ | *Alphainfluenzavirus* | *orthomyxo* | 1933 | Mill hill, London, UK | 51.62 | -0.22 | city | - | N | Transmissible in humans | N |
| *Influenza B virus* ^86^ | *Betainfluenzavirus* | *orthomyxo* | 1940 | Irvington village, Greenburgh town, New York, USA | 41.03 | -73.87 | village | - | Y | Transmissible in humans | N |
| *Influenza C virus* ^87^ | *Gammainfluenzavirus* | *orthomyxo* | 1950 | Ann Arbor city, Michigan state, USA | 42.28 | -83.74 | city | - | Y | Transmissible in humans | N |
| *Dhori thogotovirus* ^88^ | *thogotovirus* | *orthomyxo* | 1985 | Évora District, Beja District and Portalegre District, Portugal | 39.4 | -8.22 | district | 2 | Y | Strictly zoonotic | Y |
| *Thogoto thogotovirus* ^82^ | *thogotovirus* | *orthomyxo* | 1969 | Ibadan, Nigeria | 7.35 | 3.88 | city | - | Y | Strictly zoonotic | Y |
| *Avian avulavirus 1* ^89^ | *avulavirus* | *paramyxo* | 1943 | Washington, D. C., USA | 38.91 | -77.04 | city | - | Y | Strictly zoonotic | N |
| *Hendra henipavirus* ^90^ | *henipavirus* | *paramyxo* | 1995 | Hendra, Brisbane, Queensland, Australia | -27.42 | 153.07 | suburb | - | Y | Strictly zoonotic | N |
| *Nipah henipavirus* ^91^ | *henipavirus* | *paramyxo* | 1999 | Singapore | 1.36 | 103.87 | country | 1 | Y | Transmissible in humans | N |
| *Canine morbillivirus* ^92^ | *morbillivirus* | *paramyxo* | 1955 | Buffalo, New York, USA | 42.89 | -78.88 | city | - | Y | Strictly zoonotic | N |
| *Measles morbillivirus* ^93^ | *morbillivirus* | *paramyxo* | 1911 | Washington, D. C., USA | 38.91 | -77.04 | city | - | N | Transmissible in humans | N |
| *Human respirovirus 1* ^94^ | *respirovirus* | *paramyxo* | 1958 | Washington, D. C., USA | 38.91 | -77.04 | city | - | Y | Transmissible in humans | N |
| *Human respirovirus 3* ^94^ | *respirovirus* | *paramyxo* | 1958 | Washington, D. C., USA | 38.91 | -77.04 | city | - | Y | Transmissible in humans | N |
| *Achimota rubulavirus 2* ^95^ | *rubulavirus* | *paramyxo* | 2013 | Volta, Ghana | 6.05 | 0.37 | region | 2 | Y | Strictly zoonotic | N |
| *Human rubulavirus 2* ^96^ | *rubulavirus* | *paramyxo* | 1956 | Cincinnati, Ohio, USA | 39.1 | -84.51 | city | - | N | Transmissible in humans | N |
| *Human rubulavirus 4* ^97^ | *rubulavirus* | *paramyxo* | 1960 | Bethesda, Montgomery County, Maryland, USA | 38.98 | -77.09 | CDP | - | N | Transmissible in humans | N |
| *Mammalian rubulavirus 5* ^98^ | *rubulavirus* | *paramyxo* | 1959 | Stanford, Santa Clara County, California, USA | 37.42 | -122.17 | CDP | - | N | Strictly zoonotic | N |
| *Menangle rubulavirus* ^99^ | *rubulavirus* | *paramyxo* | 1998 | New South Wales, Australia | -31.25 | 146.92 | state | 2 | Y | Strictly zoonotic | N |
| *Mumps rubulavirus* ^100^ | *rubulavirus* | *paramyxo* | 1934 | Nashville, Tennessee, USA | 36.16 | -86.78 | city | - | Y | Transmissible in humans | N |
| *Simian rubulavirus* ^101^ | *rubulavirus* | *paramyxo* | 1968 | Gosen-shi city, Niigata-ken Prefecture, Japan | 37.74 | 139.18 | city | 3 | Y | Strictly zoonotic | N |
| *Sosuga rubulavirus* ^102^ | *rubulavirus* | *paramyxo* | 2014 | Area between South Sudan and Uganda | 3.76 | 32.82 | area | - | Y | Strictly zoonotic | N |
| *Tioman rubulavirus* ^103^ | *rubulavirus* | *paramyxo* | 2007 | Tioman Island, Rompin District, Pahang, Malaysia | 2.79 | 104.17 | island | - | Y | Strictly zoonotic | N |
| *Bunyamwera orthobunyavirus* ^104^ | *orthobunyavirus* | *peribunya* | 1946 | Bwamba county, Bundibugyo District, Uganda | 0.75 | 30.02 | county | 3 | Y | Strictly zoonotic | Y |
| *Bwamba orthobunyavirus* ^105^ | *orthobunyavirus* | *peribunya* | 1941 | Bwamba county, Bundibugyo District, Uganda | 0.75 | 30.02 | county | 3 | Y | Transmissible in humans | Y |
| *California encephalitis orthobunyavirus* ^106^ | *orthobunyavirus* | *peribunya* | 1952 | Kern county, California, USA | 35.49 | -118.86 | county | 3 | Y | Strictly zoonotic | Y |
| *Caraparu orthobunyavirus* ^107^ | *orthobunyavirus* | *peribunya* | 1961 | Belém, State of Pará, Brazil | -1.39 | -48.42 | city | 3 | Y | Strictly zoonotic | Y |
| *Catu orthobunyavirus* ^107^ | *orthobunyavirus* | *peribunya* | 1961 | Belém, State of Pará, Brazil | -1.39 | -48.42 | city | 3 | Y | Strictly zoonotic | Y |
| *Guama orthobunyavirus* ^107^ | *orthobunyavirus* | *peribunya* | 1961 | Belém, State of Pará, Brazil | -1.39 | -48.42 | city | 3 | Y | Strictly zoonotic | Y |
| *Guaroa orthobunyavirus* ^108^ | *orthobunyavirus* | *peribunya* | 1959 | Guaroa, Meta Department, Colombia | 3.71 | -73.24 | municipality | 3 | Y | Strictly zoonotic | Y |
| *Kairi orthobunyavirus* ^109^ | *orthobunyavirus* | *peribunya* | 1967 | Brokopondo District, Suriname | 4.77 | -55.05 | district | 2 | Y | Strictly zoonotic | Y |
| *Madrid orthobunyavirus* ^110^ | *orthobunyavirus* | *peribunya* | 1964 | Almirante Town, Changuinola District, Panama | 9.3 | -82.42 | town | - | Y | Strictly zoonotic | Y |
| *Marituba orthobunyavirus* ^107^ | *orthobunyavirus* | *peribunya* | 1961 | Belém, State of Pará, Brazil | -1.39 | -48.42 | city | 3 | Y | Strictly zoonotic | Y |
| *Nyando orthobunyavirus* ^111^ | *orthobunyavirus* | *peribunya* | 1965 | Kano Plains, near Kisumu, Kisumu County, Kenya | -0.12 | 34.97 | village | - | Y | Strictly zoonotic | Y |
| *Oriboca orthobunyavirus* ^107^ | *orthobunyavirus* | *peribunya* | 1961 | Belém, State of Pará, Brazil | -1.39 | -48.42 | city | 3 | Y | Strictly zoonotic | Y |
| *Oropouche orthobunyavirus* ^112^ | *orthobunyavirus* | *peribunya* | 1961 | Oropouche, Trinidad and Tobago | 10.22 | -61.52 | island | - | Y | Transmissible in humans | Y |
| *Patois orthobunyavirus* ^113^ | *orthobunyavirus* | *peribunya* | 1972 | Sontecomapan, Catemaco Municipality, Mexico | 18.45 | -95.1 | village&city | - | Y | Strictly zoonotic | Y |
| *Shuni orthobunyavirus* ^114^ | *orthobunyavirus* | *peribunya* | 1975 | Ibadan, Nigeria | 7.38 | 3.95 | city | - | Y | Strictly zoonotic | Y |
| *Tacaiuma orthobunyavirus* ^109^ | *orthobunyavirus* | *peribunya* | 1967 | Brokopondo District, Suriname | 4.77 | -55.05 | district | 2 | Y | Strictly zoonotic | Y |
| *Wyeomyia orthobunyavirus* ^115^ | *orthobunyavirus* | *peribunya* | 1965 | Darién Province, Panama | 7.87 | -77.84 | province | 2 | Y | Strictly zoonotic | Y |
| *Candiru phlebovirus* ^116^ | *phlebovirus* | *phenu* | 1983 | Alenquer city, state of Pará, Brazil | -1.94 | -54.73 | city | 3 | Y | Strictly zoonotic | Y |
| *Punta Toro phlebovirus* ^117^ | *phlebovirus* | *phenu* | 1970 | Jungle area of Panama | 9.5 | -79.4 | area | - | N* | Strictly zoonotic | Y |
| *Rift Valley fever phlebovirus* ^118^ | *phlebovirus* | *phenu* | 1931 | Rift Valley, Rift Valley Province, Kenya | -0.28 | 36.07 | province | 2 | Y | Transmissible in humans^†^ | Y |
| *Sandfly fever Naples phlebovirus* ^119^ | *phlebovirus* | *phenu* | 1944 | Sicily (region level), Italy | 37.6 | 14.02 | region | 2 | Y | Strictly zoonotic | Y |
| *SFTS phlebovirus* ^120^ | *phlebovirus* | *phenu* | 2011 | Huaiyangshan, China | 31.37 | 115.39 | natural region | - | Y | Transmissible in humans | Y |
| *Uukuniemi phlebovirus* ^121^ | *phlebovirus* | *phenu* | 1970 | Nitra region, Central Slovakia | 47.87 | 18.19 | region | 2 | Y | Strictly zoonotic | Y |
| *Human picobirnavirus* ^122^ | *picobirnavirus* | *picobirna* | 1988 | Rio de Janerio city, State of Rio de Janerio, Brazil | -22.91 | -43.17 | city | 3 | Y | Transmissible in humans | N |
| *Equine rhinitis A virus* ^123^ | *aphthovirus* | *picorna* | 1962 | Beckenham, Kent, UK | 51.41 | -0.03 | village | - | Y | Strictly zoonotic | N |
| *Foot-and-mouth disease virus* ^124^ | *aphthovirus* | *picorna* | 1965 | Lübeck, Schleswig-Holstein State, Germany | 53.87 | 10.69 | city | 3 | Y | Strictly zoonotic | N |
| *Cardiovirus A* ^125^ | *cardiovirus* | *picorna* | 1947 | Manila, Philippines | 14.6 | 120.98 | city | 3 | Y | Strictly zoonotic | N |
| *Cardiovirus B* ^126^ | *cardiovirus* | *picorna* | 1963 | Town of Vilyuysk, Sakha Republic, Russsia | 63.75 | 121.62 | river | - | Y | Transmissible in humans | N |
| *Cosavirus A* ^127^ | *cosavirus* | *picorna* | 2008 | Mutiple locations in Pakistan | 30.38 | 69.35 | mutiple locations^#^ | - | Y | Transmissible in humans | N |
| *Cosavirus B* ^127^ | *cosavirus* | *picorna* | 2008 | Mutiple locations in Pakistan | 30.38 | 69.35 | mutiple locations^#^ | - | Y | Transmissible in humans | N |
| *Cosavirus D* ^127^ | *cosavirus* | *picorna* | 2008 | Mutiple locations in Pakistan | 30.38 | 69.35 | mutiple locations^#^ | - | Y | Transmissible in humans | N |
| *Cosavirus E* ^128^ | *cosavirus* | *picorna* | 2008 | Melbourne, Victoria, Australia | -37.81 | 144.96 | city | - | Y | Transmissible in humans | N |
| *Cosavirus F* ^129^ | *cosavirus* | *picorna* | 2012 | Islamabad, Pakistan | 33.68 | 73.05 | city | - | Y | Transmissible in humans | N |
| *Enterovirus A* ^130^ | *enterovirus* | *picorna* | 1949 | New York State, USA | 43.3 | -74.22 | state | 2 | Y | Transmissible in humans | N |
| *Enterovirus B* ^130^ | *enterovirus* | *picorna* | 1949 | Wilmington, Delaware, USA | 39.74 | -75.54 | city | - | Y | Transmissible in humans | N |
| *Enterovirus C* ^131^ | *enterovirus* | *picorna* | 1909 | New York, New York State, USA | 40.71 | -74.01 | city | - | Y | Transmissible in humans | N |
| *Enterovirus D* ^132^ | *enterovirus* | *picorna* | 1967 | Berkeley, California, USA | 37.87 | -122.27 | city | - | N | Transmissible in humans | N |
| *Enterovirus E* ^133^ | *enterovirus* | *picorna* | 1961 | Denver, Colorado, USA | 39.74 | -104.99 | city | - | Y | Strictly zoonotic | N |
| *Enterovirus H* ^134^ | *enterovirus* | *picorna* | 1965 | Korea | 37.66 | 127.98 | country | 1 | Y | Strictly zoonotic | N |
| *Rhinovirus A* ^135^ | *enterovirus* | *picorna* | 1953 | Salisbury, UK | 51.07 | -1.79 | city | 3 | N* | Transmissible in humans | N |
| *Rhinovirus B* ^136^ | *enterovirus* | *picorna* | 1960 | Salisbury, UK | 51.07 | -1.79 | city | - | N | Transmissible in humans | N |
| *Rhinovirus C* ^137^ | *enterovirus* | *picorna* | 2006 | New York, New York State, USA | 40.71 | -74.01 | city | - | Y | Transmissible in humans | N |
| *Erbovirus A* ^138^ | *erbovirus* | *picorna* | 2005 | Styria, Austria | 47.36 | 14.47 | state | 2 | Y | Strictly zoonotic | N |
| *Hepatovirus A* ^139^ | *hepatovirus* | *picorna* | 1973 | Bethesda, Montgomery County, Maryland, USA | 38.98 | -77.09 | CDP | - | N | Transmissible in humans | N |
| *Aichivirus A* ^140^ | *kobuvirus* | *picorna* | 1991 | Aichi Prefecture, Japan | 35.18 | 136.91 | prefecture | 2 | Y | Transmissible in humans | N |
| *Parechovirus A* ^141^ | *parechovirus* | *picorna* | 1958 | Cincinnati, Ohio, USA | 39.1 | -84.51 | city | - | Y | Transmissible in humans | N |
| *Parechovirus B* ^142^ | *parechovirus* | *picorna* | 2003 | Stockholm, Sweden | 59.33 | 18.07 | city | 3 | Y | Strictly zoonotic | N |
| *Salivirus A* ^143^ | *salivirus* | *picorna* | 2009 | Northern California, USA | 38.84 | -120.9 | state | 2 | Y | Transmissible in humans | N |
| *Avian metapneumovirus* ^144^ | *metapneumovirus* | *pneumo* | 2011 | Memphis, Tennessee, USA | 35.15 | -90.05 | city | - | Y | Strictly zoonotic | N |
| *Human metapneumovirus* ^145^ | *metapneumovirus* | *pneumo* | 2001 | Rotterdam, Netherlands | 51.92 | 4.48 | city | 3 | Y | Transmissible in humans | N |
| *Human orthopneumovirus* ^146^ | *orthopneumovirus* | *pneumo* | 1957 | Baltimore, Maryland, USA | 39.29 | -76.61 | city | - | N | Transmissible in humans | N |
| *Colorado tick fever virus* ^147^ | *coltivirus* | *reo* | 1946 | Denver, Colorado, USA | 39.74 | -104.99 | city | - | N | Transmissible in humans^†^ | Y |
| *Eyach virus* ^148^ | *coltivirus* | *reo* | 1980 | Prague, Czech Republic | 50.08 | 14.44 | city | 2 | Y | Strictly zoonotic | Y |
| *Corriparta virus* ^149^ | *orbivirus* | *reo* | 1967 | Aurukun, North Queensland, Australia | -13.36 | 141.73 | town | - | Y | Strictly zoonotic | Y |
| *Great Island virus* ^150^ | *orbivirus* | *reo* | 1963 | Kemerovo District, Kemerovo Oblast, Russia | 55.35 | 86.06 | district | 3 | Y | Strictly zoonotic | Y |
| *Lebombo virus* ^114^ | *orbivirus* | *reo* | 1975 | lbadan, Nigeria | 7.38 | 3.95 | city | - | Y | Strictly zoonotic | Y |
| *Orungo virus* ^114^ | *orbivirus* | *reo* | 1976 | Ibadan, Nigeria | 7.38 | 3.95 | city | - | Y | Strictly zoonotic | Y |
| *Mammalian orthoreovirus* ^151^ | *orthoreovirus* | *reo* | 1954 | Cincinnati, Ohio, USA | 39.1 | -84.51 | city | - | Y | Transmissible in humans | N |
| *Nelson Bay orthoreovirus* ^152^ | *orthoreovirus* | *reo* | 2007 | Melaka state, Malaysia | 2.19 | 102.25 | state | 2 | Y | Transmissible in humans | N |
| *Rotavirus A* ^153^ | *rotavirus* | *reo* | 1973 | Parkville, Melbourne, Victoria, Australia | -37.81 | 144.96 | village | - | Y | Transmissible in humans | N |
| *Rotavirus B* ^154^ | *rotavirus* | *reo* | 1984 | Jinzhou, China | 41.1 | 121.13 | city | 3 | Y | Transmissible in humans | N |
| *Rotavirus C* ^155^ | *rotavirus* | *reo* | 1986 | London, United Kingdom | 51.51 | -0.13 | city | 3 | Y | Transmissible in humans | N |
| *Rotavirus H* ^156^ | *rotavirus* | *reo* | 1987 | Huaihua city, Hunan Province, China | 27.55 | 109.96 | city | 3 | Y | Transmissible in humans | N |
| *Banna virus* ^157^ | *seadornavirus* | *reo* | 1990 | Xishuangbanna, Yunnan Province, China | 22.01 | 100.8 | city | 3 | Y | Strictly zoonotic | Y |
| *Primate T-lymphotropic virus 1* ^158^ | *deltaretrovirus* | *retro* | 1980 | Bethesda, Montgomery County, Maryland, USA | 38.98 | -77.09 | CDP | - | Y | Transmissible in humans | N |
| *Primate T-lymphotropic virus 2* ^159^ | *deltaretrovirus* | *retro* | 1982 | Seattle, Washington, USA | 47.61 | -122.33 | city | - | Y | Transmissible in humans | N |
| *Primate T-lymphotropic virus 3* ^160^ | *deltaretrovirus* | *retro* | 2005 | Remote settlement in the Océan department, South Province, Cameroon | 2.5 | 10.5 | area^#^ | - | Y | Strictly zoonotic | N |
| *Human immunodeficiency virus 1* ^161^ | *lentivirus* | *retro* | 1983 | Paris, France | 48.86 | 2.35 | city | 3 | N | Transmissible in humans | N |
| *Human immunodeficiency virus 2* ^162^ | *lentivirus* | *retro* | 1986 | Dakar city, Dakar region, Senegal | 14.72 | -17.47 | city | - | Y | Transmissible in humans | N |
| *Simian immunodeficiency virus* ^163^ | *lentivirus* | *retro* | 1992 | Atlanta, Georgia, USA | 33.75 | -84.39 | city | - | N | Strictly zoonotic | N |
| *Central chimpanzee simian foamy virus* ^164^ | *simiispumavirus* | *retro* | 2012 | Near Dja Nature Reserves, Southern Cameroon | 4.5 | 13.5 | approximate location^#^ | - | Y | Strictly zoonotic | N |
| *Eastern chimpanzee simian foamy virus* ^165^ | *simiispumavirus* | *retro* | 1971 | Kenya | -0.02 | 37.91 | country | 1 | Y | Strictly zoonotic | N |
| *Grivet simian foamy virus* ^166^ | *simiispumavirus* | *retro* | 1997 | Freiburg, Baden-Württemberg, Germany | 48.00 | 7.84 | city | 3 | N | Strictly zoonotic | N |
| *Guenon simian foamy virus* ^164^ | *simiispumavirus* | *retro* | 2012 | Near lolodrof, Southern Cameroon | 3.23 | 10.73 | approximate location^#^ | - | Y | Strictly zoonotic | N |
| *Taiwanese macaque simian foamy virus* ^167^ | *simiispumavirus* | *retro* | 2002 | Ottawa, Canada | 45.42 | -75.7 | city | 3 | Y | Strictly zoonotic | N |
| *Australian bat lyssavirus* ^168^ | *lyssavirus* | *rhabdo* | 1998 | Brisbane, Queensland, Australia | -27.47 | 153.03 | city | - | Y | Strictly zoonotic | N |
| *Duvenhage lyssavirus* ^169^ | *lyssavirus* | *rhabdo* | 1971 | Pretoria, Tshwane City, Gauteng province, South Africa | -25.75 | 28.23 | city | - | Y | Strictly zoonotic | N |
| *European bat 1 lyssavirus* ^170^ | *lyssavirus* | *rhabdo* | 1989 | Belgorod, Belgorod Oblast, Russia | 50.6 | 36.6 | City | 3 | Y | Strictly zoonotic | N |
| *European bat 2 lyssavirus* ^171^ | *lyssavirus* | *rhabdo* | 1986 | Helsinki, Uusimaa region, Finland | 60.17 | 24.94 | city | - | Y | Strictly zoonotic | N |
| *Irkut lyssavirus* ^172^ | *lyssavirus* | *rhabdo* | 2013 | Tonghua county, Tonghua City, Jilin Province, China | 41.68 | 125.76 | county | - | Y | Strictly zoonotic | N |
| *Mokola lyssavirus* ^173^ | *lyssavirus* | *rhabdo* | 1972 | Ibadan, Nigeria | 7.38 | 3.95 | city | - | Y | Strictly zoonotic | N |
| *Rabies lyssavirus* ^174^ | *lyssavirus* | *rhabdo* | 1903 | Pavia, province of Pavia, region of Lombardy, Italy | 45.18 | 9.16 | city | - | N | Transmissible in humans^†^ | N |
| *Bas-Congo tibrovirus* ^175^ | *tibrovirus* | *rhabdo* | 2012 | Mangala village, Boma Bungu Health Zone, DRC | -4.04 | 21.76 | village | - | Y | Transmissible in humans | Y |
| *Ekpoma 1 tibrovirus* ^176^ | *tibrovirus* | *rhabdo* | 2015 | Irrua, Edo State, Nigeria | 6.74 | 6.22 | village | - | Y | Strictly zoonotic | N |
| *Ekpoma 2 tibrovirus* ^176^ | *tibrovirus* | *rhabdo* | 2015 | Irrua, Edo State, Nigeria | 6.74 | 6.22 | village | - | Y | Strictly zoonotic | N |
| *Alagoas vesiculovirus* ^177^ | *vesiculovirus* | *rhabdo* | 1967 | State of Alagoas, Brazil | -9.57 | -36.78 | state | 2 | Y | Strictly zoonotic | Y |
| *Chandipura vesiculovirus* ^178^ | *vesiculovirus* | *rhabdo* | 1967 | Nagpur city, Nagpur district, Maharashtra state, India | 21.15 | 79.09 | city | - | Y | Strictly zoonotic | Y |
| *Cocal vesiculovirus* ^179^ | *vesiculovirus* | *rhabdo* | 1964 | Nariva swamp, Trinidad and Tobago | 10.43 | -61.06 | island | - | Y | Strictly zoonotic | Y |
| *Indiana vesiculovirus* ^180^ | *vesiculovirus* | *rhabdo* | 1958 | Beltsville, Prince George's County, Maryland, USA | 39.05 | -76.9 | CDP | - | Y | Strictly zoonotic | Y |
| *Isfahan vesiculovirus* ^181^ | *vesiculovirus* | *rhabdo* | 1977 | Dormian village, Isfahan Province, Iran | 33.28 | 52.36 | village | - | Y | Strictly zoonotic | Y |
| *Maraba vesiculovirus* ^182^ | *vesiculovirus* | *rhabdo* | 1984 | Serra Norte area, State of Pará, Brazil | -6.04 | -50.18 | area | - | Y | Strictly zoonotic | Y |
| *New Jersey vesiculovirus* ^183^ | *vesiculovirus* | *rhabdo* | 1950 | Madison, Wisconsin, USA | 43.07 | -89.4 | city | - | Y | Strictly zoonotic | Y |
| *Piry vesiculovirus* ^184^ | *vesiculovirus* | *rhabdo* | 1974 | Marabá city, State of Pará, Brazil | -5.38 | -49.13 | city | 3 | Y | Strictly zoonotic | Y |
| *Barmah Forest virus* ^185^ | *alphavirus* | *toga* | 1986 | South coast of New South Wales, Australia | -36.68 | 149.66 | state | 2 | Y | Transmissible in humans | Y |
| *Chikungunya virus* ^186^ | *alphavirus* | *toga* | 1956 | Newala district, Tanzania | -10.64 | 39.24 | district | 3 | Y | Transmissible in humans | Y |
| *Eastern equine encephalitis virus* ^187^ | *alphavirus* | *toga* | 1938 | Southwestern Massachusetts, USA | 42.20 | -71.10 | state | 2 | Y | Strictly zoonotic | Y |
| *Everglades virus* ^188^ | *alphavirus* | *toga* | 1970 | Homestead, Florida, USA | 25.47 | -80.48 | city | - | Y | Strictly zoonotic | Y |
| *Getah virus* ^189^ | *alphavirus* | *toga* | 1966 | Brisbane, Queensland, Australia | -27.47 | 153.025 | city | - | Y | Strictly zoonotic | Y |
| *Highlands J virus* ^190^ | *alphavirus* | *toga* | 2000 | Florida, USA | 27.66 | -81.52 | state | 2 | Y | Strictly zoonotic | Y |
| *Madariaga virus* ^191^ | *alphavirus* | *toga* | 1972 | Port of Spain, Trinidad | 10.67 | -61.52 | city | 2 | Y | Strictly zoonotic | Y |
| *Mayaro virus* ^192^ | *alphavirus* | *toga* | 1957 | Mayaro County, Trinidad and Tobago | 10.28 | -61.03 | county | - | Y | Strictly zoonotic | Y |
| *Mosso das Pedras virus* ^193^ | *alphavirus* | *toga* | 2013 | Chaco province, Argentina | -27.43 | -59.02 | state | 2 | Y | Strictly zoonotic | Y |
| *Mucambo virus* ^194^ | *alphavirus* | *toga* | 1965 | Mexico city, Mexico | 19.43 | -99.13 | city | 2 | Y | Strictly zoonotic | Y |
| *Ndumu virus* ^195^ | *alphavirus* | *toga* | 1961 | Ndumu, KwaZulu-Natal Province, South Africa | -26.93 | 32.26 | town/city | - | Y | Strictly zoonotic | Y |
| *Onyong-nyong virus* ^196^ | *alphavirus* | *toga* | 1961 | Entebbe, Wakiso District, Uganda | 0.05 | 32.46 | city | 3 | Y | Transmissible in humans | Y |
| *Pixuna virus* ^197^ | *alphavirus* | *toga* | 1991 | Belém, State of Pará, Brazil | -1.39 | -48.42 | city | 3 | Y | Strictly zoonotic | Y |
| *Rio Negro virus* ^198^ | *alphavirus* | *toga* | 1993 | General Belgrano Island, Formosa, Argentina | -26.19 | -58.18 | island | - | Y | Strictly zoonotic | Y |
| *Ross River virus* ^199^ | *alphavirus* | *toga* | 1972 | Edward River, New South Wales, Australia | -35.4 | 144.25 | river | - | Y | Transmissible in humans | Y |
| *Semliki Forest virus* ^200^ | *alphavirus* | *toga* | 1979 | Gießen, Gießen District, Hesse State, Germany | 50.58 | 8.68 | town | - | Y | Transmissible in humans | Y |
| *Sindbis virus* ^201^ | *alphavirus* | *toga* | 1955 | Cairo city, Cairo Governorate, Egypt | 30.04 | 31.24 | city | - | Y | Strictly zoonotic | Y |
| *Tonate virus* ^202^ | *alphavirus* | *toga* | 1976 | French Guyana, now French Guiana | 3.93 | -53.13 | Region | 2 | Y | Strictly zoonotic | Y |
| *Una virus* ^203^ | *alphavirus* | *toga* | 1963 | Belém, State of Pará, Brazil | -1.39 | -48.42 | city | 3 | Y | Strictly zoonotic | Y |
| *Venezuelan equine encephalitis virus* ^204^ | *alphavirus* | *toga* | 1943 | New York, New York State, USA | 40.71 | -74.01 | city | - | Y | Transmissible in humans | Y |
| *Western equine encephalitis virus* ^187^ | *alphavirus* | *toga* | 1938 | Fresno, California, USA | 36.75 | -119.77 | city | - | Y | Transmissible in humans^†^ | Y |
| *Whataroa virus* ^205^ | *alphavirus* | *toga* | 1964 | Greymouth town, Grey District, New Zealand | -42.45 | 171.21 | town | - | Y | Strictly zoonotic | Y |
| *Rubella virus* ^206^ | *rubivirus* | *toga* | 1942 | Washington, D. C., USA | 38.91 | -77.04 | city | - | Y | Transmissible in humans | N |
| *Hepatitis delta virus* ^207^ | *deltavirus* | *Unassigned* | 1977 | Turin, Piedmont region, Italy | 45.07 | 7.69 | city | - | N | Transmissible in humans | N |

DRC, Democratic Republic of the Congo; CDP, census-designated place; ^#^ locations inferred from maps given in the discovery papers; * location of the research field; lat and lon are the latitude and longitude of the centroid of the discovery location; ^†^ transmitted only via iatrogenic or maternal routes

# References

1 Parodi AS, Greenway DJ, Rugiero HR, et al. [Concerning the epidemic outbreak in Junin]. *Dia Med* 1958; **30**(62): 2300–1.

2 Lisieux T, Coimbra M, Nassar ES, et al. New arenavirus isolated in Brazil. *Lancet* 1994; **343**(8894): 391–2.

3 Buchmeier M, Adam E, Rawls WE. Serological evidence of infection by Pichinde virus among laboratory workers. *Infect Immun* 1974; **9**(5): 821–3.

4 Delgado S, Erickson BR, Agudo R, et al. Chapare virus, a newly discovered arenavirus isolated from a fatal hemorrhagic fever case in Bolivia. *PLoS Pathog* 2008; **4**(4): e1000047.

5 Salas R, de Manzione N, Tesh RB, et al. Venezuelan haemorrhagic fever. *Lancet* 1991; **338**(8774): 1033–6.

6 Buckley SM, Casals J. Lassa fever, a new virus disease of man from West Africa. 3. Isolation and characterization of the virus. *Am J Trop Med Hyg* 1970; **19**(4): 680–91.

7 Briese T, Paweska JT, McMullan LK, et al. Genetic detection and characterization of Lujo virus, a new hemorrhagic fever-associated arenavirus from southern Africa. *PLoS Pathog* 2009; **5**(5): e1000455.

8 Armstrong C, Lillie RD. Experimental Lymphocytic Choriomeningitis of Monkeys and Mice Produced by a Virus Encountered in Studies of the 1933 St. Louis Encephalitis Epidemic. *Public Health Reports (1896-1970)* 1934; **49**(35): 1019–27.

9 Mackenzie RB, Beye HK, Valverde L, Garron H. Epidemic Hemorrhagic Fever in Bolivia. I. A Preliminary Report of the Epidemiologic and Clinical Findings in a New Epidemic Area in South America. *Am J Trop Med Hyg* 1964; **13**: 620–5.

10 Georges AJ, Gonzalez JP, Abdul-Wahid S, Saluzzo JF, Meunier DM, McCormick JB. Antibodies to Lassa and Lassa-like viruses in man and mammals in the Central African Republic. *Trans R Soc Trop Med Hyg* 1985; **79**(1): 78–9.

11 Enserink M. Emerging diseases. New arenavirus blamed for recent deaths in California. *Science* 2000; **289**(5481): 842–3.

12 Appleton H, Higgins PG. Letter: Viruses and gastroenteritis in infants. *Lancet* 1975; **1**(7919): 1297.

13 Finkbeiner SR, Allred AF, Tarr PI, Klein EJ, Kirkwood CD, Wang D. Metagenomic analysis of human diarrhea: viral detection and discovery. *PLoS Pathog* 2008; **4**(2): e1000011.

14 Finkbeiner SR, Holtz LR, Jiang Y, et al. Human stool contains a previously unrecognized diversity of novel astroviruses. *Virol J* 2009; **6**: 161.

15 Finkbeiner SR, Li Y, Ruone S, et al. Identification of a novel astrovirus (astrovirus VA1) associated with an outbreak of acute gastroenteritis. *J Virol* 2009; **83**(20): 10836–9.

16 Rott R, Herzog S, Fleischer B, et al. Detection of serum antibodies to Borna disease virus in patients with psychiatric disorders. *Science* 1985; **228**(4700): 755–6.

17 Hoffmann B, Tappe D, Hoper D, et al. A Variegated Squirrel Bornavirus Associated with Fatal Human Encephalitis. *N Engl J Med* 2015; **373**(2): 154–62.

18 Kapikian AZ, Wyatt RG, Dolin R, Thornhill TS, Kalica AR, Chanock RM. Visualization by immune electron microscopy of a 27-nm particle associated with acute infectious nonbacterial gastroenteritis. *J Virol* 1972; **10**(5): 1075–81.

19 Chiba S, Sakuma Y, Kogasaka R, et al. Fecal shedding of virus in relation to the days of illness in infantile gastroenteritis due to calicivirus. *J Infect Dis* 1980; **142**(2): 247–9.

20 Smith AW, Berry ES, Skilling DE, et al. In vitro isolation and characterization of a calicivirus causing a vesicular disease of the hands and feet. *Clin Infect Dis* 1998; **26**(2): 434–9.

21 Terao Y, Takagi H, Phan TG, Okitsu S, Ushijima H. Identification of antibody against porcine coronavirus in human milk. *Clin Lab* 2007; **53**(3–4): 129–30.

22 Hamre D, Procknow JJ. A new virus isolated from the human respiratory tract. *Proc Soc Exp Biol Med* 1966; **121**(1): 190–3.

23 Fouchier RA, Hartwig NG, Bestebroer TM, et al. A previously undescribed coronavirus associated with respiratory disease in humans. *Proc Natl Acad Sci U S A* 2004; **101**(16): 6212–6.

24 McIntosh K, Dees JH, Becker WB, Kapikian AZ, Chanock RM. Recovery in tracheal organ cultures of novel viruses from patients with respiratory disease. *Proc Natl Acad Sci U S A* 1967; **57**(4): 933–40.

25 Woo PC, Lau SK, Chu CM, et al. Characterization and complete genome sequence of a novel coronavirus, coronavirus HKU1, from patients with pneumonia. *J Virol* 2005; **79**(2): 884–95.

26 Zaki AM, van Boheemen S, Bestebroer TM, Osterhaus AD, Fouchier RA. Isolation of a novel coronavirus from a man with pneumonia in Saudi Arabia. *N Engl J Med* 2012; **367**(19): 1814–20.

27 Peiris JS, Lai ST, Poon LL, et al. Coronavirus as a possible cause of severe acute respiratory syndrome. *Lancet* 2003; **361**(9366): 1319–25.

28 Beards GM, Hall C, Green J, Flewett TH, Lamouliatte F, Du Pasquier P. An enveloped virus in stools of children and adults with gastroenteritis that resembles the Breda virus of calves. *Lancet* 1984; **1**(8385): 1050–2.

29 Towner JS, Sealy TK, Khristova ML, et al. Newly discovered ebola virus associated with hemorrhagic fever outbreak in Uganda. *PLoS Pathog* 2008; **4**(11): e1000212.

30 Miranda ME, White ME, Dayrit MM, Hayes CG, Ksiazek TG, Burans JP. Seroepidemiological study of filovirus related to Ebola in the Philippines. *Lancet* 1991; **337**(8738): 425–6.

31 Bowen ET, Lloyd G, Harris WJ, Platt GS, Baskerville A, Vella EE. Viral haemorrhagic fever in southern Sudan and northern Zaire. Preliminary studies on the aetiological agent. *Lancet* 1977; **1**(8011): 571–3.

32 Le Guenno B, Formenty P, Wyers M, Gounon P, Walker F, Boesch C. Isolation and partial characterisation of a new strain of Ebola virus. *Lancet* 1995; **345**(8960): 1271–4.

33 Johnson KM, Lange JV, Webb PA, Murphy FA. Isolation and partial characterisation of a new virus causing acute haemorrhagic fever in Zaire. *Lancet* 1977; **1**(8011): 569–71.

34 Martini GA, Knauff HG, Schmidt HA, Mayer G, Baltzer G. A hitherto unknown infectious disease contracted from monkeys. "Marburg-virus" disease. *Ger Med Mon* 1968; **13**(10): 457–70.

35 Srihongse S, Johnson CM. The first isolation of Bussuquara virus from man. *Trans R Soc Trop Med Hyg* 1971; **65**(4): 541–2.

36 Bondre VP, Sapkal GN, Yergolkar PN, et al. Genetic characterization of Bagaza virus (BAGV) isolated in India and evidence of anti-BAGV antibodies in sera collected from encephalitis patients. *J Gen Virol* 2009; **90**(Pt 11): 2644–9.

37 Smithburn KC, Paterson HE, Heymann CS, Winter PA. An agent related to Uganda S virus from man and mosquitoes in South Africa. *S Afr Med J* 1959; **33**: 959–62.

38 Batista WC, Tavares Gda S, Vieira DS, Honda ER, Pereira SS, Tada MS. Notification of the first isolation of Cacipacore virus in a human in the State of Rondonia, Brazil. *Rev Soc Bras Med Trop* 2011; **44**(4): 528–30.

39 Ashburn PM, Craig CF. Experimental Inverstigations Regarding the Etiology of Dengue Fever. *The Journal of Infectious Diseases* 1907; **4**(3): 440–75.

40 Hawkes RA, Boughton CR, Naim HM, Wild J, Chapman B. Arbovirus infections of humans in New South Wales. Seroepidemiology of the flavivirus group of togaviruses. *Med J Aust* 1985; **143**(12–13): 555–61.

41 Humphery-Smith I, Cybinski DH, Byrnes KA, St George TD. Seroepidemiology of arboviruses among seabirds and island residents of the Great Barrier Reef and Coral Sea. *Epidemiol Infect* 1991; **107**(2): 435–40.

42 Laemmert HW, Jr., Hughes TP. The virus of Ilheus encephalitis; isolation, serological specificity and transmission. *J Immunol* 1947; **55**(1): 61–7.

43 Hayashi M. Übertragung des Virus von Encephalitis epidemica japonica auf Affen*. *Psychiatry and Clinical Neurosciences* 1935; **1**(1): 419–65.

44 Work TH, Trapido H. Summary of preliminary report of investigations of the Virus Research Centre on an epidemic disease affecting forest villagers and wild monkeys of Shimoga District, Mysore. *Indian J Med Sci* 1957; **11**(5): 341–2.

45 Smith CE. A virus resembling Russian spring-summer encephalitis virus from an ixodid tick in Malaya. *Nature* 1956; **178**(4533): 581–2.

46 Rivers TM, Schwentker FF. Louping Ill in Man. *J Exp Med* 1934; **59**(5): 669–85.

47 French EL. Murray Valley encephalitis isolation and characterization of the aetiological agent. *Med J Aust* 1952; **1**(4): 100–3.

48 Smithburn KC. Neutralizing antibodies against certain recently isolated viruses in the sera of human beings residing in East Africa. *J Immunol* 1952; **69**(2): 223–34.

49 Chumakov MP. Results of a study made of Omsk hemorrhagic fever (OL) by an expedition of the Institute of Neurology. *Vestn. Acad. Med. Nauk SSSR* 1948; **2**(19).

50 Mc LD, Donohue WL. Powassan virus: isolation of virus from a fatal case of encephalitis. *Can Med Assoc J* 1959; **80**(9): 708–11.

51 Sulkin SE, Burns KF, Shelton DF, Wallis C. Bat salivary gland virus: infections of man and monkey. *Tex Rep Biol Med* 1962; **20**: 113–27.

52 Webster LT, Fite GL. A Virus Encountered in the Study of Material from Cases of Encephalitis N the St. Louis and Kansas City Epidemics of 1933. *Science* 1933; **78**(2029): 463–5.

53 Bowen ET, Simpson DI, Platt GS, et al. Arbovirus infections in Sarawak, October 1968-February 1970: human serological studies in a land Dyak village. *Trans R Soc Trop Med Hyg* 1975; **69**(2): 182–6.

54 Solowjow WD. Zur Aetiologie der Zecken- Encephalitis. *Acta Med Urss* 1938; **1**(4): 484–92.

55 Dick GW, Haddow AJ. Uganda S virus; a hitherto unrecorded virus isolated from mosquitoes in Uganda. I. Isolation and pathogenicity. *Trans R Soc Trop Med Hyg* 1952; **46**(6): 600–18.

56 Pecorari M, Longo G, Gennari W, et al. First human case of Usutu virus neuroinvasive infection, Italy, August-September 2009. *Euro Surveill* 2009; **14**(50).

57 Smithburn KC, Kokernot RH, Weinbren MP, De Meillon B. Studies on arthropod-borne viruses of Tongaland. IX. Isolation of Wesselsbron virus from a naturally infected human being and from Aedes (Banksinella) circumluteolus Theo. *S Afr J Med Sci* 1957; **22**(2–3): 113–20.

58 Smithburn KC, Hughes TP, Burke AW, Paul JH. A Neurotropic Virus Isolated from the Blood of a Native of Uganda. *The American Journal of Tropical Medicine and Hygiene* 1940 **s1–20**(4): 471 – 92.

59 Reed W, Carroll JS, Agramonte A. The etiology of yellow fever: An additional note. *Journal of the American Medical Association* 1901; **XXXVI**(7): 431–40.

60 Dick GW. Zika virus. II. Pathogenicity and physical properties. *Trans R Soc Trop Med Hyg* 1952; **46**(5): 521–34.

61 Choo QL, Kuo G, Weiner AJ, Overby LR, Bradley DW, Houghton M. Isolation of a cDNA clone derived from a blood-borne non-A, non-B viral hepatitis genome. *Science* 1989; **244**(4902): 359–62.

62 Yoshiba M, Okamoto H, Mishiro S. Detection of the GBV-C hepatitis virus genome in serum from patients with fulminant hepatitis of unknown aetiology. *Lancet* 1995; **346**(8983): 1131–2.

63 Kapoor A, Kumar A, Simmonds P, et al. Virome Analysis of Transfusion Recipients Reveals a Novel Human Virus That Shares Genomic Features with Hepaciviruses and Pegiviruses. *MBio* 2015; **6**(5): e01466–15.

64 Giangaspero M, Wellemans G, Vanopdenbosch E, Belloli A, Verhulst A. Bovine viral diarrhoea. *Lancet* 1988; **2**(8602): 110.

65 Lopez N, Padula P, Rossi C, Lazaro ME, Franze-Fernandez MT. Genetic identification of a new hantavirus causing severe pulmonary syndrome in Argentina. *Virology* 1996; **220**(1): 223–6.

66 Morzunov SP, Feldmann H, Spiropoulou CF, et al. A newly recognized virus associated with a fatal case of hantavirus pulmonary syndrome in Louisiana. *J Virol* 1995; **69**(3): 1980–3.

67 Ravkov EV, Rollin PE, Ksiazek TG, Peters CJ, Nichol ST. Genetic and serologic analysis of Black Creek Canal virus and its association with human disease and Sigmodon hispidus infection. *Virology* 1995; **210**(2): 482–9.

68 Vincent MJ, Quiroz E, Gracia F, et al. Hantavirus pulmonary syndrome in Panama: identification of novel hantaviruses and their likely reservoirs. *Virology* 2000; **277**(1): 14–9.

69 Gligic A, Dimkovic N, Xiao SY, et al. Belgrade virus: a new hantavirus causing severe hemorrhagic fever with renal syndrome in Yugoslavia. *J Infect Dis* 1992; **166**(1): 113–20.

70 Lee HW, Lee PW, Johnson KM. Isolation of the etiologic agent of Korean Hemorrhagic fever. *J Infect Dis* 1978; **137**(3): 298–308.

71 Johnson AM, Bowen MD, Ksiazek TG, et al. Laguna Negra virus associated with HPS in western Paraguay and Bolivia. *Virology* 1997; **238**(1): 115–27.

72 Brummer-Korvenkontio M, Vaheri A, Hovi T, et al. Nephropathia epidemica: detection of antigen in bank voles and serologic diagnosis of human infection. *J Infect Dis* 1980; **141**(2): 131–4.

73 Klempa B, Koivogui L, Sylla O, et al. Serological evidence of human hantavirus infections in Guinea, West Africa. *J Infect Dis* 2010; **201**(7): 1031–4.

74 Song G, Qiu XZ, Ni DS, Zhao JN, Kong BX. [Etiological studies of epidemic hemorrhagic fever. I. Virus isolation in Apodemus agrarius from non-endemic area and its antigenic characterization]. *Zhongguo Yi Xue Ke Xue Yuan Xue Bao* 1982; **4**(2): 73–7.

75 Nichol ST, Spiropoulou CF, Morzunov S, et al. Genetic identification of a hantavirus associated with an outbreak of acute respiratory illness. *Science* 1993; **262**(5135): 914–7.

76 Pattamadilok S, Lee BH, Kumperasart S, et al. Geographical distribution of hantaviruses in Thailand and potential human health significance of Thailand virus. *Am J Trop Med Hyg* 2006; **75**(5): 994–1002.

77 Okumura M, Yoshimatsu K, Kumperasart S, et al. Development of serological assays for Thottapalayam virus, an insectivore-borne Hantavirus. *Clin Vaccine Immunol* 2007; **14**(2): 173–81.

78 Vapalahti O, Lundkvist A, Kukkonen SK, et al. Isolation and characterization of Tula virus, a distinct serotype in the genus Hantavirus, family Bunyaviridae. *J Gen Virol* 1996; **77 ( Pt 12)**: 3063–7.

79 Balayan MS, Andjaparidze AG, Savinskaya SS, et al. Evidence for a virus in non-A, non-B hepatitis transmitted via the fecal-oral route. *Intervirology* 1983; **20**(1): 23–31.

80 Sridhar S, Yip CCY, Wu S, et al. Rat Hepatitis E Virus as Cause of Persistent Hepatitis after Liver Transplant. *Emerg Infect Dis* 2018; **24**(12): 2241–50.

81 Simpson DI, Knight EM, Courtois G, Williams MC, Weinbren MP, Kibukamusoke JW. Congo virus: a hitherto undescribed virus occurring in Africa. I. Human isolations--clinical notes. *East Afr Med J* 1967; **44**(2): 86–92.

82 Causey OR, Kemp GE, Madbouly MH, Lee VH. Arbovirus surveillance in Nigeria, 1964-1967. *Bull Soc Pathol Exot Filiales* 1969; **62**(2): 249–53.

83 Dandawate CN, Work TH, Webb JK, Shah KV. Isolation of Ganjam virus from a human case of febrile illness: a report of a laboratory infection and serological survey of human sera from three different states of India. *Indian J Med Res* 1969; **57**(6): 975–82.

84 Chastel C, Main AJ, Richard P, Le Lay G, Legrand-Quillien MC, Beaucournu JC. Erve virus, a probable member of Bunyaviridae family isolated from shrews (Crocidura russula) in France. *Acta Virol* 1989; **33**(3): 270–80.

85 Smith W, Andrewes CH, Laidlaw PP. A virus obtained from influenza patients. *Lancet* 1933; **222**(5732): 66–68.

86 Thomas Francis J. Differentiation of Influenza A and Influenza B by the Complement-Fixation Reaction. *Proceedings of the Society for Experimental Biology and Medicine* 1940; **45**(3): 861–63.

87 Francis T, Jr., Quilligan JJ, Jr., Minuse E. Identification of another epidemic respiratory disease. *Science* 1950; **112**(2913): 495–7.

88 Filipe AR, Calisher CH, Lazuick J. Antibodies to Congo-Crimean haemorrhagic fever, Dhori, Thogoto and Bhanja viruses in southern Portugal. *Acta Virol* 1985; **29**(4): 324–8.

89 Burnet FM. Human infection with the virus of Newcastle disease of fowls. *The Medical Journal of Australia* 1943; **30**(6): 313–14.

90 Selvey L, Sheridan J. Outbreak of Severe Respiratory Disease in Humans and Horses Due to a Previously Unrecognized Paramyxovirus. *J Travel Med* 1995; **2**(4): 275.

91 Lee KE, Umapathi T, Tan CB, et al. The neurological manifestations of Nipah virus encephalitis, a novel paramyxovirus. *Ann Neurol* 1999; **46**(3): 428–32.

92 Karzon DT. Studies on a neutralizing antibody against canine distemper virus found in man. *Pediatrics* 1955; **16**(6): 809–18.

93 Goldberger J, Anderson JF. The nature of the virus of measles. *Journal of the American Medical Association* 1911; **LVII**(12): 971–72.

94 Chanock RM, Parrott RH, Cook K, et al. Newly recognized myxoviruses from children with respiratory disease. *N Engl J Med* 1958; **258**(5): 207–13.

95 Baker KS, Todd S, Marsh GA, et al. Novel, potentially zoonotic paramyxoviruses from the African straw-colored fruit bat Eidolon helvum. *J Virol* 2013; **87**(3): 1348–58.

96 Chanock RM. Association of a new type of cytopathogenic myxovirus with infantile croup. *J Exp Med* 1956; **104**(4): 555–76.

97 Johnson KM, Chanock RM, Cook MK, Huebner RJ. Studies of a new human hemadsorption virus. I. Isolation, properties and characterization. *Am J Hyg* 1960; **71**: 81–92.

98 Schultz EW, Habel K. SA virus; a new member of the myxovirus group. *J Immunol* 1959; **82**(3): 274–8.

99 Philbey AW, Kirkland PD, Ross AD, et al. An apparently new virus (family Paramyxoviridae) infectious for pigs, humans, and fruit bats. *Emerg Infect Dis* 1998; **4**(2): 269–71.

100 Johnson CD, Goodpasture EW. An Investigation of the Etiology of Mumps. *J Exp Med* 1934; **59**(1): 1–19.

101 Itoh H, Morimoto Y, Doi Y, Sanpe T. Studies on simian viruses some properties of SV 41 grown in vero cell cultures and search for serum neutralizing antibodies in humans and various animals. *Uirusu* 1968; **18**(6): 495–503.

102 Albarino CG, Foltzer M, Towner JS, et al. Novel paramyxovirus associated with severe acute febrile disease, South Sudan and Uganda, 2012. *Emerg Infect Dis* 2014; **20**(2): 211–6.

103 Yaiw KC, Crameri G, Wang L, et al. Serological evidence of possible human infection with Tioman virus, a newly described paramyxovirus of bat origin. *J Infect Dis* 2007; **196**(6): 884–6.

104 Smithburn KC, Haddow AJ, Mahaffy AF. A neurotropic virus isolated from Aedes mosquitoes caught in the Semliki forest. *Am J Trop Med Hyg* 1946; **26**: 189–208.

105 Smithburn KC, Mahaffy AF, Paul JH. Bwamba Fever and Its Causative Virus. *The American Journal of Tropical Medicine and Hygiene* 1941; **s1–21**(1): 75 – 90.

106 Hammon WM, Reeves WC. California encephalitis virus, a newly described agent. *Calif Med* 1952; **77**(5): 303–9.

107 Causey OR, Causey CE, Maroja OM, Macedo DG. The isolation of arthropod-borne viruses, including members of two hitherto undescribed serological groups, in the Amazon region of Brazil. *Am J Trop Med Hyg* 1961; **10**: 227–49.

108 Groot H, Oya A, Bernal C, Barreto-Reyes P. Guaroa virus, a new agent isolated in Colombia, South America. *Am J Trop Med Hyg* 1959; **8**: 604–9.

109 van Tongeren HA. Occurrence of arboviruses belonging to the C-, Bunyamwera and Guama groups, and of Oropouche, Junin, Tacaiuma and Kwatta viruses in man in the province of Brokopondo, Surinam: a serological survey. *Trop Geogr Med* 1967; **19**(4): 309–25.

110 Derodaniche E, Paesdeandrade A, Galindo P. Isolation of Two Antigenically Distinct Arthropod-Borne Viruses of Group C in Panama. *Am J Trop Med Hyg* 1964; **13**: 839–43.

111 Williams MC, Woodall JP, Corbet PS. Nyando Virus: A Hitherto Undescribed Virus Isolated from Anopheles Funestus Giles Collected in Kenya. *Arch Gesamte Virusforsch* 1965; **15**: 422–7.

112 Anderson CR, Spence L, Downs WG, Aitken TH. Oropouche virus: a new human disease agent from Trinidad, West Indies. *Am J Trop Med Hyg* 1961; **10**: 574–8.

113 Scherer WF, Anderson K, Dickerman RW, Ordonez JV. Studies of Patois group arboviruses in Mexico, Guatemala, Honduras, and British Honduras. *Am J Trop Med Hyg* 1972; **21**(2): 194–200.

114 Moore DL, Causey OR, Carey DE, et al. Arthropod-borne viral infections of man in Nigeria, 1964–1970. *Ann Trop Med Parasitol* 1975; **69**(1): 49–64.

115 Sirhongse S, Johnson CM. Wyeomyia Subgroup of Arbovirus: Isolation from Man. *Science* 1965; **149**(3686): 863–4.

116 Travassos da Rosa AP, Tesh RB, Pinheiro FP, Travassos da Rosa JF, Peterson NE. Characterization of eight new phlebotomus fever serogroup arboviruses (Bunyaviridae: Phlebovirus) from the Amazon region of Brazil. *Am J Trop Med Hyg* 1983; **32**(5): 1164–71.

117 Sather GE. Punta Toro (PT) strain. D 4021A. *Am J Trop Med Hyg* 1970; **19**(6): Suppl:1103–4.

118 Daubney R, Hudson JR, Garnham PC. Enzootic hepatitis or rift valley fever. An undescribed virus disease of sheep cattle and man from east africa. *The Journal of Pathology and Bacteriology* 1931; **34**(4): 545–79.

119 Sabin AB, Philip CB, Paul JR. Phlebotomus (pappataci or sandfly) fever: A disease of military importance summary of existing knowledge and preliminary report of original investigations. *Journal of the American Medical Association* 1944; **125**(9): 603–06.

120 Zhang YZ, Zhou DJ, Xiong Y, et al. Hemorrhagic fever caused by a novel tick-borne Bunyavirus in Huaiyangshan, China. *Zhonghua Liu Xing Bing Xue Za Zhi* 2011; **32**(3): 209–20.

121 Sekeyová M, Gresíková M, Stúpalová S. Serological study on distribution of Uukuniemi virus in man. *Folia Parasitologica* 1970; **17**(4): 341–43.

122 Pereira HG, Fialho AM, Flewett TH, Teixeira JM, Andrade ZP. Novel viruses in human faeces. *Lancet* 1988; **2**(8602): 103–4.

123 Plummer G. An equine respiratory virus with enterovirus properties. *Nature* 1962; **195**: 519–20.

124 Pilz W, Garbe HG. [Further cases of foot-and-mouth disease virus infections in man]. *Zentralbl Bakteriol Orig* 1965; **198**(1): 154–7.

125 Smadel JE, Warren J. The virus of encephalomyocarditis and its apparent causation of disease in man. *J Clin Invest* 1947; **26**(6): 1197.

126 Casals J. Immunological Characterization of Vilyuisk Human Encephalomyelitis Virus. *Nature* 1963; **200**: 339–41.

127 Kapoor A, Victoria J, Simmonds P, et al. A highly prevalent and genetically diversified Picornaviridae genus in South Asian children. *Proc Natl Acad Sci U S A* 2008; **105**(51): 20482–7.

128 Holtz LR, Finkbeiner SR, Kirkwood CD, Wang D. Identification of a novel picornavirus related to cosaviruses in a child with acute diarrhea. *Virol J* 2008; **5**: 159.

129 Kapusinszky B, Phan TG, Kapoor A, Delwart E. Genetic diversity of the genus Cosavirus in the family Picornaviridae: a new species, recombination, and 26 new genotypes. *PLoS One* 2012; **7**(5): e36685.

130 Sickles GM, Dalldorf G. Serologic differences among strains of the Coxsackie group of viruses. *Proc Soc Exp Biol Med* 1949; **72**(1): 30.

131 Flexner S, Lewis PA. The transmission of acute poliomyelitis to monkeys. *Journal of the American Medical Association* 1909; **LIII**(20): 1639–39.

132 Schieble JH, Fox VL, Lennette EH. A probable new human picornavirus associated with respiratory diseases. *Am J Epidemiol* 1967; **85**(2): 297–310.

133 Moscovici C, Laplaca M, Maisel J, Kempe H. Studies of bovine enteroviruses. *Am J Vet Res* 1961; **22**: 852–63.

134 Conrad ME. Infectious hepatitis: An unresolved military problem. *Mil Med* 1965; **130**: 594–600.

135 Andrewes CH, Chaproniere DM, Gompels AE, Pereira HG, Roden AT. Propagation of common-cold virus in tissue cultures. *Lancet* 1953; **265**(6785): 546–7.

136 Tyrrell DA, Parsons R. Some virus isolations from common colds. III. Cytopathic effects in tissue cultures. *Lancet* 1960; **1**(7118): 239–42.

137 Lau SK, Yip CC, Tsoi HW, et al. Clinical features and complete genome characterization of a distinct human rhinovirus (HRV) genetic cluster, probably representing a previously undetected HRV species, HRV-C, associated with acute respiratory illness in children. *J Clin Microbiol* 2007; **45**(11): 3655–64.

138 Kriegshauser G, Deutz A, Kuechler E, Skern T, Lussy H, Nowotny N. Prevalence of neutralizing antibodies to Equine rhinitis A and B virus in horses and man. *Vet Microbiol* 2005; **106**(3–4): 293–6.

139 Feinstone SM, Kapikian AZ, Purceli RH. Hepatitis A: detection by immune electron microscopy of a viruslike antigen associated with acute illness. *Science* 1973; **182**(4116): 1026–8.

140 Yamashita T, Kobayashi S, Sakae K, et al. Isolation of cytopathic small round viruses with BS-C-1 cells from patients with gastroenteritis. *J Infect Dis* 1991; **164**(5): 954–7.

141 Ramos-Alvarez M, Sabin AB. Enteropathogenic viruses and bacteria; role in summer diarrheal diseases of infancy and early childhood. *J Am Med Assoc* 1958; **167**(2): 147–56.

142 Niklasson B, Heller KE, Schonecker B, et al. Development of type 1 diabetes in wild bank voles associated with islet autoantibodies and the novel ljungan virus. *Int J Exp Diabesity Res* 2003; **4**(1): 35–44.

143 Greninger AL, Runckel C, Chiu CY, et al. The complete genome of klassevirus - a novel picornavirus in pediatric stool. *Virol J* 2009; **6**: 82.

144 Kayali G, Ortiz EJ, Chorazy ML, et al. Serologic evidence of avian metapneumovirus infection among adults occupationally exposed to Turkeys. *Vector Borne Zoonotic Dis* 2011; **11**(11): 1453–8.

145 van den Hoogen BG, de Jong JC, Groen J, et al. A newly discovered human pneumovirus isolated from young children with respiratory tract disease. *Nat Med* 2001; **7**(6): 719–24.

146 Chanock R, Roizman B, Myers R. Recovery from infants with respiratory illness of a virus related to chimpanzee coryza agent (CCA). I. Isolation, properties and characterization. *Am J Hyg* 1957; **66**(3): 281–90.

147 Florio L, Stewart MO, Mugrage ER. The Etiology of Colorado Tick Fever. *J Exp Med* 1946; **83**(1): 1–10.

148 Malkova D, Holubova J, Kolman JM, et al. Antibodies against some arboviruses in persons with various neuropathies. *Acta Virol* 1980; **24**(4): 298.

149 DOHERTY RL. STUDIES OF ABORIGINES AT AURUKUN AND WEIPA MISSIONS, NORTH QUEENSLAND 2. OTHER LABORATORY STUDIES. *Journal of Paediatrics and Child Health* 1967; **3**(4): 213–18.

150 Chumakov MP, Sarmanova ES, Bychkova MV, et al. [Identification of the Virus of Kemerovo Tick-Borne Fever. Evidence of the Antigenic Independence of This Virus]. *Vopr Virusol* 1963; **29**: 440–4.

151 Ramos-Alvarez M, Sabin AB. Characteristics of poliomyelitis and other enteric viruses recovered in tissue culture from healthy American children. *Proc Soc Exp Biol Med* 1954; **87**(3): 655–61.

152 Chua KB, Crameri G, Hyatt A, et al. A previously unknown reovirus of bat origin is associated with an acute respiratory disease in humans. *Proc Natl Acad Sci U S A* 2007; **104**(27): 11424–9.

153 Bishop RF, Davidson GP, Holmes IH, Ruck BJ. Virus particles in epithelial cells of duodenal mucosa from children with acute non-bacterial gastroenteritis. *Lancet* 1973; **2**(7841): 1281–3.

154 Hung T, Chen GM, Wang CG, et al. Waterborne outbreak of rotavirus diarrhoea in adults in China caused by a novel rotavirus. *Lancet* 1984; **1**(8387): 1139–42.

155 Bridger JC, Pedley S, McCrae MA. Group C rotaviruses in humans. *J Clin Microbiol* 1986; **23**(4): 760–3.

156 Wang C, Hu C, Huang F, X. C, Hung T. A novel discovered rotavirus from adult acute diarrhoeal patients in China [Article in Chinese]. Chinese Journal of Virology. 1987;03(04):321-5+411.

157 Xu PT, Wang YM, Zuo JM, Lin JW, Xu PM. New orbiviruses isolated from patients with unknown fever and encephalitis in Yunnan Province. *Chinese Journal of Virology* 1990; **6**(1): 27–34.

158 Poiesz BJ, Ruscetti FW, Gazdar AF, Bunn PA, Minna JD, Gallo RC. Detection and isolation of type C retrovirus particles from fresh and cultured lymphocytes of a patient with cutaneous T-cell lymphoma. *Proc Natl Acad Sci U S A* 1980; **77**(12): 7415–9.

159 Kalyanaraman VS, Sarngadharan MG, Robert-Guroff M, Miyoshi I, Golde D, Gallo RC. A new subtype of human T-cell leukemia virus (HTLV-II) associated with a T-cell variant of hairy cell leukemia. *Science* 1982; **218**(4572): 571–3.

160 Calattini S, Chevalier SA, Duprez R, et al. Discovery of a new human T-cell lymphotropic virus (HTLV-3) in Central Africa. *Retrovirology* 2005; **2**: 30.

161 Barre-Sinoussi F, Chermann JC, Rey F, et al. Isolation of a T-lymphotropic retrovirus from a patient at risk for acquired immune deficiency syndrome (AIDS). *Science* 1983; **220**(4599): 868–71.

162 Kanki PJ, Barin F, M'Boup S, et al. New human T-lymphotropic retrovirus related to simian T-lymphotropic virus type III (STLV-IIIAGM). *Science* 1986; **232**(4747): 238–43.

163 Khabbaz RF, Rowe T, Murphey-Corb M, et al. Simian immunodeficiency virus needlestick accident in a laboratory worker. *Lancet* 1992; **340**(8814): 271–3.

164 Rua R, Betsem E, Calattini S, Saib A, Gessain A. Genetic characterization of simian foamy viruses infecting humans. *J Virol* 2012; **86**(24): 13350–9.

165 Achong BG, Mansell PW, Epstein MA. A new human virus in cultures from a nasopharyngeal carcinoma. *J Pathol* 1971; **103**(2): P18.

166 Schweizer M, Falcone V, Gange J, Turek R, Neumann-Haefelin D. Simian foamy virus isolated from an accidentally infected human individual. *J Virol* 1997; **71**(6): 4821–4.

167 Brooks JI, Rud EW, Pilon RG, Smith JM, Switzer WM, Sandstrom PA. Cross-species retroviral transmission from macaques to human beings. *Lancet* 2002; **360**(9330): 387–8.

168 Samaratunga H, Searle JW, Hudson N. Non-rabies Lyssavirus human encephalitis from fruit bats: Australian bat Lyssavirus (pteropid Lyssavirus) infection. *Neuropathol Appl Neurobiol* 1998; **24**(4): 331–5.

169 Meredith CD, Prossouw AP, Koch H. An unusual case of human rabies thought to be of chiropteran origin. *S Afr Med J* 1971; **45**(28): 767–9.

170 Selimov MA, Tatarov AG, Botvinkin AD, Klueva EV, Kulikova LG, Khismatullina NA. Rabies-related Yuli virus; identification with a panel of monoclonal antibodies. *Acta Virol* 1989; **33**(6): 542–6.

171 Lumio J, Hillbom M, Roine R, et al. Human rabies of bat origin in Europe. *Lancet* 1986; **1**(8477): 378.

172 Liu Y, Zhang S, Zhao J, Zhang F, Hu R. Isolation of Irkut virus from a Murina leucogaster bat in China. *PLoS Negl Trop Dis* 2013; **7**(3): e2097.

173 Familusi JB, Osunkoya BO, Moore DL, Kemp GE, Fabiyi A. A fatal human infection with Mokola virus. *Am J Trop Med Hyg* 1972; **21**(6): 959–63.

174 Negri A. Beitrag zum Studium der Aetiologie der Tollwuth. *Zeitschrift für Hygiene und Infektionskrankheiten* 1903; **43**(1): 507–28.

175 Grard G, Fair JN, Lee D, et al. A novel rhabdovirus associated with acute hemorrhagic fever in central Africa. *PLoS Pathog* 2012; **8**(9): e1002924.

176 Stremlau MH, Andersen KG, Folarin OA, et al. Discovery of novel rhabdoviruses in the blood of healthy individuals from West Africa. *PLoS Negl Trop Dis* 2015; **9**(3): e0003631.

177 Federer KE, Burrows R, Brooksby JB. Vesicular stomatitis virus--the relationship between some strains of the Indiana serotype. *Res Vet Sci* 1967; **8**(1): 103–17.

178 Bhatt PN, Rodrigues FM. Chandipura: a new Arbovirus isolated in India from patients with febrile illness. *Indian J Med Res* 1967; **55**(12): 1295–305.

179 Jonkers AH, Shope RE, Aitken TH, Spence L. COCAL VIRUS, A NEW AGENT IN TRINIDAD RELATED TO VESICULAR STOMATITIS VIRUS, TYPE INDIANA. *Am J Vet Res* 1964; **25**: 236–42.

180 Patterson WC, Mott LO, Jenney EW. A study of vesicular stomatitis in man. *J Am Vet Med Assoc* 1958; **133**(1): 57–62.

181 Tesh R, Saidi S, Javadian E, Loh P, Nadim A. Isfahan virus, a new vesiculovirus infecting humans, gerbils, and sandflies in Iran. *Am J Trop Med Hyg* 1977; **26**(2): 299–306.

182 Travassos da Rosa AP, Tesh RB, Travassos da Rosa JF, Herve JP, Main AJ, Jr. Carajas and Maraba viruses, two new vesiculoviruses isolated from phlebotomine sand flies in Brazil. *Am J Trop Med Hyg* 1984; **33**(5): 999–1006.

183 Hanson RP, Rasmussen AF, Jr., Brandly CA, Brown JW. Human infection with the virus of vesicular stomatitis. *J Lab Clin Med* 1950; **36**(5): 754–8.

184 Pinheiro FP, Bensabath G, Andrade AH, et al. Infectious diseases along Brazil's trans-amazon highway: surveillance and research. *Bull Pan Am Health Organ* 1974; **8**(2): 111–22.

185 Vale TG, Carter IW, McPhie KA, James GS, Cloonan MJ. Human arbovirus infections along the south coast of New South Wales. *Aust J Exp Biol Med Sci* 1986; **64 ( Pt 3)**: 307–9.

186 Ross RW. The Newala epidemic. III. The virus: isolation, pathogenic properties and relationship to the epidemic. *J Hyg (Lond)* 1956; **54**(2): 177–91.

187 Howitt B. Recovery of the Virus of Equine Encephalomyelitis from the Brain of a Child. *Science* 1938; **88**(2289): 455–6.

188 Ehrenkranz NJ, Sinclair MC, Buff E, Lyman DO. The natural occurrence of Venezuelan equine encephalitis in the United States. *N Engl J Med* 1970; **282**(6): 298–302.

189 Doherty RL. Arthropod-borne viruses in Australia and their relation to infection and disease. *Prog Med Virol* 1974; **17**(0): 136–92.

190 Meehan PJ, Wells DL, Paul W, et al. Epidemiological features of and public health response to a St. Louis encephalitis epidemic in Florida, 1990-1. *Epidemiol Infect* 2000; **125**(1): 181–8.

191 Luciani K, Abadia I, Martinez-Torres AO, et al. Madariaga virus infection associated with a case of acute disseminated encephalomyelitis. *Am J Trop Med Hyg* 2015; **92**(6): 1130–2.

192 Anderson CR, Downs WG, Wattley GH, Ahin NW, Reese AA. Mayaro virus: a new human disease agent. II. Isolation from blood of patients in Trinidad, B.W.I. *Am J Trop Med Hyg* 1957; **6**(6): 1012–6.

193 Pisano MB, Oria G, Beskow G, et al. Venezuelan equine encephalitis viruses (VEEV) in Argentina: serological evidence of human infection. *PLoS Negl Trop Dis* 2013; **7**(12): e2551.

194 Demucha Macias J, S'Anchez Spindola I. TWO HUMAN CASES OF LABORATORY INFECTION WITH MUCAMBO VIRUS. *Am J Trop Med Hyg* 1965; **14**: 475–8.

195 Kokernot RH, McIntosh BM, Worth CB. Ndumu virus, a hitherto unknown agent, isolated from culicine mosouitoes collected in northern Natal. Union of South Africa. *Am J Trop Med Hyg* 1961; **10**: 383–6.

196 Williams MC, Woodall JP. O'nyong-nyong fever: an epidemic virus disease in East Africa. II. Isolation and some properties of the virus. *Trans R Soc Trop Med Hyg* 1961; **55**: 135–41.

197 Vasconcelos PF, Da Rosa JF, Da Rosa AP, Degallier N, Pinheiro Fde P, Sa Filho GC. [Epidemiology of encephalitis caused by arbovirus in the Brazilian Amazonia]. *Rev Inst Med Trop Sao Paulo* 1991; **33**(6): 465–76.

198 Contigiani MS, de Basualdo M, Camara A, et al. [Presence of antibodies against Venezuelan equine encephalitis virus subtype VI in patients with acute febrile illness]. *Rev Argent Microbiol* 1993; **25**(4): 212–20.

199 Doherty RL, Carley JG, Best JC. Isolation of Ross River virus from man. *Med J Aust* 1972; **1**(21): 1083–4.

200 Willems WR, Kaluza G, Boschek CB, et al. Semliki forest virus: cause of a fatal case of human encephalitis. *Science* 1979; **203**(4385): 1127–9.

201 Taylor RM, Hurlbut HS, Work TH, Kingston JR, Frothingham TE. Sindbis virus: a newly recognized arthropodtransmitted virus. *Am J Trop Med Hyg* 1955; **4**(5): 844–62.

202 Digoutte JP, Girault G. [The protective properties in mice of tonate virus and two strains of cabassou virus against neurovirulent everglades Venezuelan encephalitis virus (author's transl)]. *Ann Microbiol (Paris)* 1976; **127B**(3): 429–37.

203 Causey OR, Casals J, Shope RE, Udomsakdi S. Aura and Una, Two New Group a Arthropod-Borne Viruses. *Am J Trop Med Hyg* 1963; **12**: 777–81.

204 Casals J, Curnen EC, Thomas L. Venezuelan Equine Encephalomyelitis in Man. *J Exp Med* 1943; **77**(6): 521–30.

205 Ross RW, Miles JA, Austin FJ, Maguire T. INVESTIGATIONS INTO THE ECOLOGY OF A GROUP A ARBOVIRUS IN WESTLAND, NEW ZEALAND. *Aust J Exp Biol Med Sci* 1964; **42**: 689–702.

206 Habel K. Transmission of Rubella to Macacus mulatta Monkeys. *Public Health Reports (1896–1970)* 1942; **57**(31): 1126–39.

207 Rizzetto M, Canese MG, Arico S, et al. Immunofluorescence detection of new antigen-antibody system (delta/anti-delta) associated to hepatitis B virus in liver and in serum of HBsAg carriers. *Gut* 1977; **18**(12): 997–1003.
