# Supplementary material for: Global discovery of human-infective RNA viruses: A modelling analysis
Source: PLoS Pathog. 2020 Nov 30;16(11):e1009079. doi: 10.1371/journal.ppat.1009079 (PMC7728385; doi:10.1371/journal.ppat.1009079)
Supplement: S2 Table — (DOCX) [file ppat.1009079.s010.docx]

**S2 Table Resolution and covered grid cells for virus discovery data**

|  | **Polygon data** | | |  | **Point data** | **Total** |
| --- | --- | --- | --- | --- | --- | --- |
|  | **Country level** | **State level** | **City level** |  |  |  |
| **Virus counts** | 3 (1.4%) | 44 (19.7%) | 50 (22.4%) |  | 126 (56.5%) | 223 |
| **Gridded cell counts** | 74 | 507 | 53 |  | 191^*^ | 825 |

^*^Grid cell counts here include viruses first detected in multiple points from the literature
