# Supplementary material for: Global discovery of human-infective RNA viruses: A modelling analysis
Source: PLoS Pathog. 2020 Nov 30;16(11):e1009079. doi: 10.1371/journal.ppat.1009079 (PMC7728385; doi:10.1371/journal.ppat.1009079)
Supplement: S3 Table — (DOCX) [file ppat.1009079.s011.docx]

## S3 Table List of explanatory factors included in the model

| **Variable of interest** | **Definition** | **Group** | **Resolution** | **Source** |
| --- | --- | --- | --- | --- |
| Temperature | 1. Annual mean temperature (℃) from 1901 to 2015: calculated from monthly average temperature  2. Annual mean temperature change (℃) from 1901 to 2015: calculated from monthly average temperature  3. Maximum temperature of the warmest month (℃) from 1901 to 2015: calculated from monthly maximum temperature  4. Minimum temperature of the coldest month (℃) from 1901 to 2015: calculated from monthly minimum temperature  5. Annual mean diurnal temperature range (℃) from 1901 to 2015: calculated from monthly diurnal temperature range | Climatic | 0.5° | Climatic Research Unit (CRU): High-resolution gridded datasets^1^ |
| Precipitation | 1. Annual total precipitation (mm) from 1901 and 2015: calculated by summing the monthly precipitation  2. Annual total precipitation change (mm) from 1901 and 2015: calculated by summing the monthly precipitation  3. Maximum precipitation (mm) from 1901 to 2015: precipitation of wettest month, calculated from monthly precipitation  4. Minimum precipitation (mm) from 1901 to 2015: precipitation of driest month, calculated from monthly precipitation  5. Annual rainy days (counts) from 1901 to 2015: calculated by summing the monthly rainy-day counts | Climatic | 0.5° | Climatic Research Unit (CRU): High-resolution gridded datasets^1^ |
| Latitude | In angular (ranges from 0° to 90° ) | Climatic | - | - |
| Human population | 1. Human population count from 1970 to 2000 (counts, in persons)  2. Population growth: The decadal difference of human population (counts, in persons) | Socio-economic | 30" | Global Rural-Urban Mapping project (GRUMP)^2^ |
| Gross domestic product (GDP) | 1. Mean GDP (unit: PPP, billion US$2005/yr) for three time steps: 1980, 1990, 2000 and 2010, which are estimated by downscaling actual GDPs by country  2. GDP growth: The decadal difference of GDP (unit: PPP, billion US$2005/yr) | Socio-economic | 0.5° | Centre for Global Environmental Research (CGER): Global dataset of gridded population and GDP scenarios^3^ |
| University count | Higher education institutions count offering at least a 4-year professional diploma or a post-graduate degree in each country | Socio-economic | Country level | World Higher Education Database (WHED) |
| Land use | The percentage of 1. Cropland, 2. Pasture, 3. Urban land, 4. Primary land, and 5. Secondary land in each grid cell from 1900 to 2015.  Primary land: natural vegetation (either forest or non-forest) that has never been impacted by human activities since 1700; Secondary land is natural vegetation (either forest or non-forest) that is recovering from previous human disturbance.  6. Growth of cropland area, 7. Growth of pasture area, 8. Growth of urbanized land area, 9. Growth of primary land area, and 10. Growth of secondary land area: The percentage of land area change for each category in each grid cell from 1900 to 2015  11. Urbanization of cropland, 12. Urbanization of pasture, 13. Urbanization of primary land, and 14. Urbanization of secondary land: The percentage of land area change from cropland/pasture/primary land/secondary land to urban land in each grid cell from 1900 to 2015  15. The percentage of cultivated and managed vegetation in each grid cell | Land use | 0.5°/30" | Harmonized Global Land Use^4^  EarthEnv^5^ |
| Mammal species richness | Mammal species richness for 2015 represented the number of species in a particular class, family or International Union for the Conservation of Nature (IUCN) threatened category. | Biodiversity | 30" | Gridded Species Distribution^6^ |
| Livestock headcount | Domestic animal headcount, summed cattle, buffalo, sheep, goats, pigs | Biodiversity | 30" | Gridded Livestock of the World (GLW)^7^ |

**Reference**

1. Harris I, Jones PD, Osborn TJ, et al. Updated high-resolution grids of monthly climatic observations – the CRU TS3.10 Dataset. *International Journal of Climatology* 2014;34(3):623-42. doi: doi:10.1002/joc.3711

2. Center for International Earth Science Information Network - CIESIN - Columbia University. Global Population Count Grid Time Series Estimates. Palisades, NY: NASA Socioeconomic Data and Applications Center (SEDAC), 2017.

3. Murakami D, Yamagata Y. Estimation of gridded population and GDP scenarios with spatially explicit statistical downscaling; 2016.

4. Chini LP, Hurtt GC, Frolking S. Harmonized Global Land Use for Years 1500 -2100, V1: ORNL Distributed Active Archive Center, 2014.

5. Tuanmu M-N, Jetz W. A global 1-km consensus land-cover product for biodiversity and ecosystem modelling. *Global Ecology and Biogeography* 2014;23(9):1031-45. doi: doi:10.1111/geb.12182

6. International Union for Conservation of Nature - IUCN, Center for International Earth Science Information Network - CIESIN - Columbia University. Gridded Species Distribution: Global Mammal Richness Grids, 2015 Release. Palisades, NY: NASA Socioeconomic Data and Applications Center (SEDAC), 2015.

7. Robinson TP, Wint GR, Conchedda G, et al. Mapping the global distribution of livestock. *PloS one* 2014;9(5):e96084. doi: 10.1371/journal.pone.0096084 [published Online First: 2014/05/31]
