# Supplementary material for: Global discovery of human-infective RNA viruses: A modelling analysis
Source: PLoS Pathog. 2020 Nov 30;16(11):e1009079. doi: 10.1371/journal.ppat.1009079 (PMC7728385; doi:10.1371/journal.ppat.1009079)
Supplement: S4 Table — (DOCX) [file ppat.1009079.s012.docx]

**S4 Table Model validation statistics for stratified analyses**

| **Model** | **% of deviance explained (95% quantiles)** | **ICC (95% quantiles)** |
| --- | --- | --- |
| **Strictly zoonotic** | 60.0% (53.9%–65.3%) | 0.44 (0.36–0.51) |
| **Transmissible** | 44.7% (40.1%–49.7%) | 0.63 (0.55–0.70) |
| **Vector-borne** | 63.3% (55.6%–71.7%) | 0.43 (0.34–0.52) |
| **Non-vector-borne** | 40.8% (36.2% –45.3%) | 0.67 (0.62–0.73) |

ICC, infraclass correlation coefficient
