# Supplementary material for: Global discovery of human-infective RNA viruses: A modelling analysis
Source: PLoS Pathog. 2020 Nov 30;16(11):e1009079. doi: 10.1371/journal.ppat.1009079 (PMC7728385; doi:10.1371/journal.ppat.1009079)
Supplement: S5 Table — (DOCX) [file ppat.1009079.s013.docx]

## S5 Table Model parameters for sensitivity analyses and stratified analyses

| **Model** | **Tree complexity** | **Learning rate** | **Bag fraction** | **No. of trees** |
| --- | --- | --- | --- | --- |
| **Sensitivity analysis (1)** | 2 | 0.0015 | 0.5 | 1129 |
| **Sensitivity analysis (2)** | 5 | 0.0030 | 0.5 | 1051 |
| **Strictly zoonotic** | 4 | 0.0020 | 0.5 | 1114 |
| **Transmissible** | 2 | 0.0020 | 0.5 | 1430 |
| **Vector-borne** | 2 | 0.0040 | 0.5 | 1147 |
| **Non-vector-borne** | 2 | 0.0035 | 0.5 | 1080 |

(1) Using data from 1980 to 2000 only; (2) Removing the 22 discovery reports that were not patients’ locations
