# Supplementary material for: Global discovery of human-infective RNA viruses: A modelling analysis
Source: PLoS Pathog. 2020 Nov 30;16(11):e1009079. doi: 10.1371/journal.ppat.1009079 (PMC7728385; doi:10.1371/journal.ppat.1009079)
Supplement: S1 Text — (DOCX) [file ppat.1009079.s014.docx]

## S1 Text. Georeferencing human RNA virus discovery locations

The discovery locations were georeferenced as precisely as possible according to the original literature, ranging from precise coordinates of points to polygon-level data (e.g., city, county, district, state, or country) (**S1 Table**). Where no point location was provided, location was assigned to a set of grid cells corresponding to the maximum resolution available (e.g. city, district, country). A spatial polygon was created for those locations at administrative level 3 and above. As shown in **S1 Table**, administrative divisions of different countries vary: level 3 may be city, county, district, municipality; level 2 includes state, province, region, department, district, prefecture, city, county; level 1 is country. All grid cells falling into these polygons were recorded. Grid cells that have more than ½ area falling in the polygon were also added manually. If no grid cell was identified, the coordinate of the centroid of the polygon was used instead. For locations equal to and lower than administrative level 4, we used the grid cell that the centroid of the location falls into. **S2 Table** summarized types of data within the occurrence database. The majority of occurrence records involved point data (56.0%), while the remainder were recorded at city or state or even country level.

Thirty-five viruses were found in multiple locations according to the first published report. For these, we selected one of these locations based on three criteria in order: (i) the virus being named after the location; (ii) the location was the same as first author or the laboratory; (iii) the location with the highest number of cases.
