# Supplementary material for: Global discovery of human-infective RNA viruses: A modelling analysis
Source: PLoS Pathog. 2020 Nov 30;16(11):e1009079. doi: 10.1371/journal.ppat.1009079 (PMC7728385; doi:10.1371/journal.ppat.1009079)
Supplement: S2 Text — (DOCX) [file ppat.1009079.s015.docx]

## S2 Text. Transformation of resolution for explanatory factors and data extrapolation

All explanatory factors and virus locations were matched by 1° spatial grid cell, having rescaled or transformed the data where necessary. For gridded data at the 30" and 0.5° resolution, we have rescaled them to 1° resolution by using the resampling approach in R package “raster”. The “bilinear” interpolation was used to compute values for the re-gridded data.

For data at country level, we first assigned each country to the grid cells it covers, which can be realized by the “over” function in R package “sp”, and then matched variables at country level with gridded data by coordinates.

The BRT model matches RNA virus discovery count in each grid cell with historical decadal climatic variables, population, GDP, and land use, so we extrapolated the data for these variables back to 1901 (Population and GDP only, as climatic variables and land use data have full temporary coverage from 1901 to 2015).

We extrapolated the data for population and GDP by using the growth rate at grid cell level or country level. Gridded data for population from 1970 to 2000 was available from SEDAC’s Global Rural-Urban Mapping Project. Gridded population counts after 2000 were estimated from the gridded population data from 2000 to 2020, which is also provided by SEDAC (<http://sedac.ciesin.columbia.edu/data/set/gpw-v4-population-count-rev10>). To reconcile the population difference from the two databases, we calculated the population growth rate in each grid cell after 2000 based on the gridded data from 2000 to 2020, ignoring the absolute population size. We then estimated the population after 2000 for each grid cell by multiplying the value of gridded population in 2000 (from gridded population data from 1970 to 2000) by the growth rate we calculated above. Data before 1970 were estimated by population growth at country level, as there is no population data at grid level for this stage. We assumed that the population growth rate for each grid cell in the same country was similar. The total population for each country was obtained from Our World in Data (<https://ourworldindata.org/world-population-growth>), and the growth rate of population for each country before 1970 was derived from these data. Similarly, by applying the growth rate for the gridded population data for 1970, we calculated the population in each grid cell from 1900 to 1960.

Data for GDP before 1980 were estimated by GDP growth at country level. The GDP for each country was obtained from Our World in Data (<https://ourworldindata.org/grapher/world-gdp-over-the-last-two-millennia>).
