# Supplementary material for: Global discovery of human-infective RNA viruses: A modelling analysis
Source: PLoS Pathog. 2020 Nov 30;16(11):e1009079. doi: 10.1371/journal.ppat.1009079 (PMC7728385; doi:10.1371/journal.ppat.1009079)
Supplement: S3 Text — (DOCX) [file ppat.1009079.s016.docx]

## S3 Text. Result of model validation

The model’s predictive performance was assessed by calculating the cross-validated explained deviance of the bootstrap model (a measure of the goodness-of-fit between the predicted and raw data), ^1^ as well as by conducting 50 rounds of ten-fold cross-validation. Fifty data sets were randomly selected from the data for 1000 replicated models to conduct 50 rounds of ten-fold cross-validation. As before, 223 discovery present and 446 discovery absent samples were drawn in each of the 50 rounds, and the calculated virus count in each grid cell by decade was used as the response. The dataset of each round was randomly divided into ten subsets. For each round of ten-fold cross-validation, ten different training sets comprising unique combinations of 9 subsets were used to fit models, and the remaining one was used to evaluate the predictive performance of the model as a test set. The median infraclass correlation coefficient (ICC) with 95% quantiles was used as the validation statistics. The ICC ranges from 0 and 1, with an ICC of less than 0.40 suggesting a poor predictive ability, 0.40–0.59 suggesting a fair predictive ability, 0.60–0.74 suggesting a good predictive ability, and 0.75–1 suggesting an excellent model. ^2^

The bootstrap 1000 replicate BRT full model explained a median of 40.9% of deviance in the data (95% quantiles: 36.5%–45.5%). Using 50 runs of 10-fold cross-validation, the model had a median ICC of 0.55 (95% quantiles: 0.50–0.61). The model validation statistics for the stratified model were shown in **S4 Table**. Combining these measures, our BRT model predictions range from fair to good. ^2^

**Reference**

1. Elith J, Leathwick JR, Hastie T. A working guide to boosted regression trees. *The Journal of animal ecology* 2008;77(4):802-13. doi: 10.1111/j.1365-2656.2008.01390.x [published Online First: 2008/04/10]

2. Cicchetti DV. Guidelines, criteria, and rules of thumb for evaluating normed and standardized assessment instruments in psychology. *Psychol Assess* 1994;6(4):284-90. doi: 10.1037/1040-3590.6.4.284
