# Supplementary material for: Global discovery of human-infective RNA viruses: A modelling analysis
Source: PLoS Pathog. 2020 Nov 30;16(11):e1009079. doi: 10.1371/journal.ppat.1009079 (PMC7728385; doi:10.1371/journal.ppat.1009079)

**S4 Text. Source and permission for the world shapefile used in the study**

The world shapefile used in the study was obtained from Data and Maps for ArcGIS (formerly Esri Data & Maps, <https://www.arcgis.com/home/group.html?id=24838c2d95e14dd18c25e9bad55a7f82#overview>). The source file for data redistribution rights was attached. The specific file we used–continent.gdb–was under ‘Yes 1, 2, 3’; detailed descriptions of the redistribution rights were shown blow.

“Yes 1” Redistribution rights are granted by the data vendor for hard-copy renditions or static, electronic map images (e.g. .gif, .jpeg, etc.) that are plotted, printed, or publicly displayed with proper metadata and source/copyright attribution to the respective data vendor(s).

“Yes 2” Geodata are redistributable with a Value-Added Software Application developed by Esri Business Partners on a royalty-free basis with proper metadata and source/copyright attribution to the respective data vendor(s).

“Yes 3” Geodata are redistributable without a Value-Added Software Application (i.e., adding the sample data to an existing, [non]commercial dataset for redistribution) with proper metadata and source/copyright attribution to the respective data vendor(s).


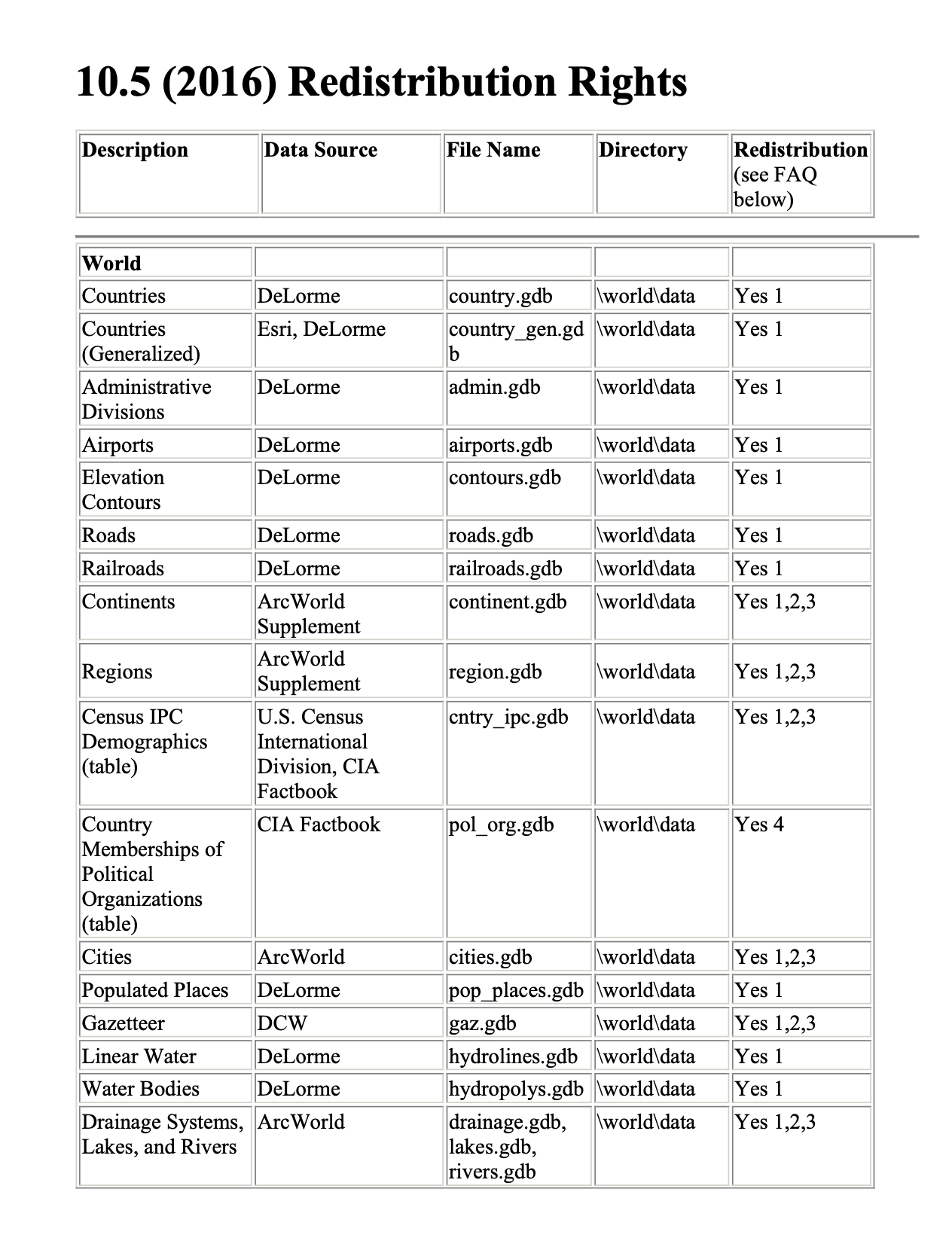


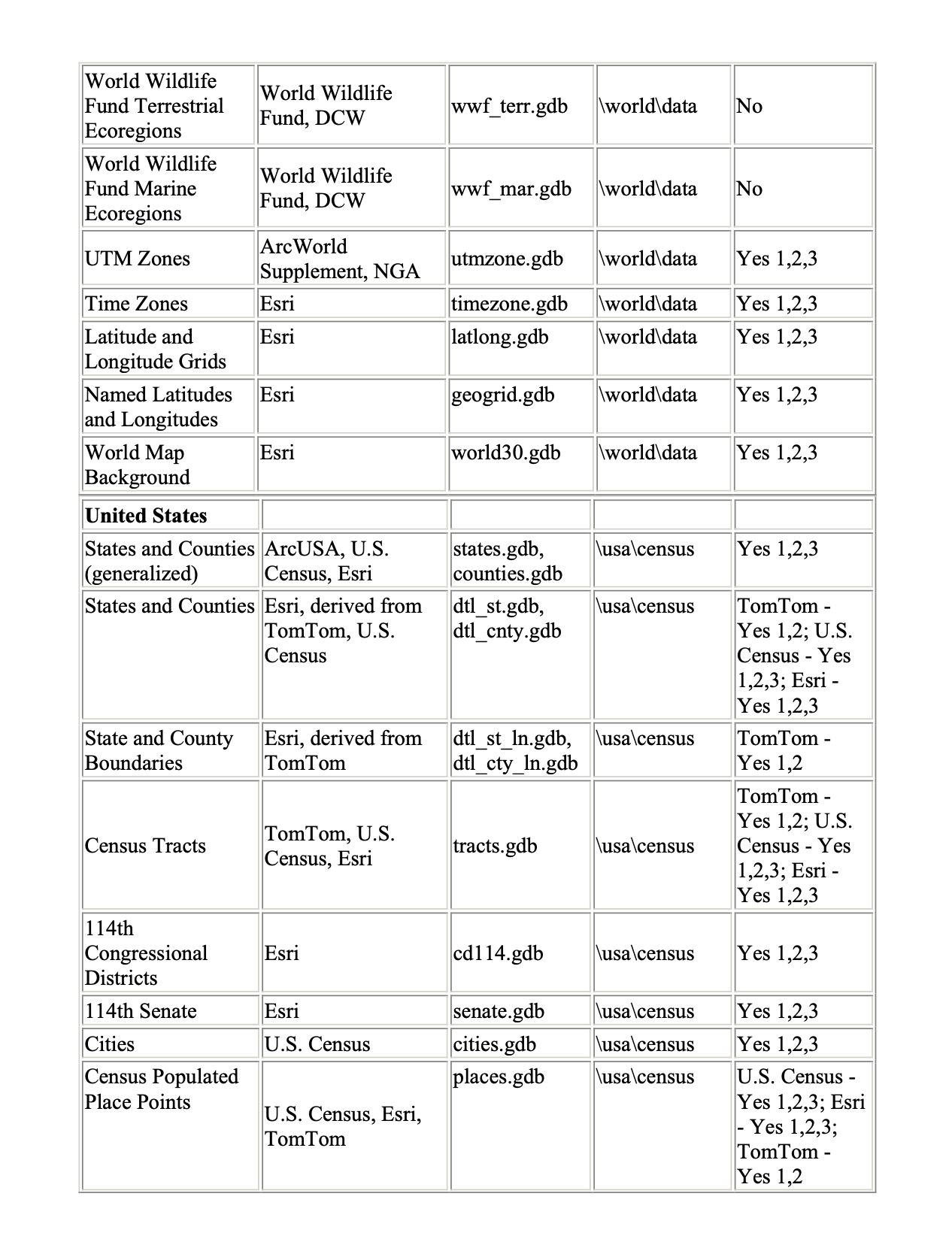


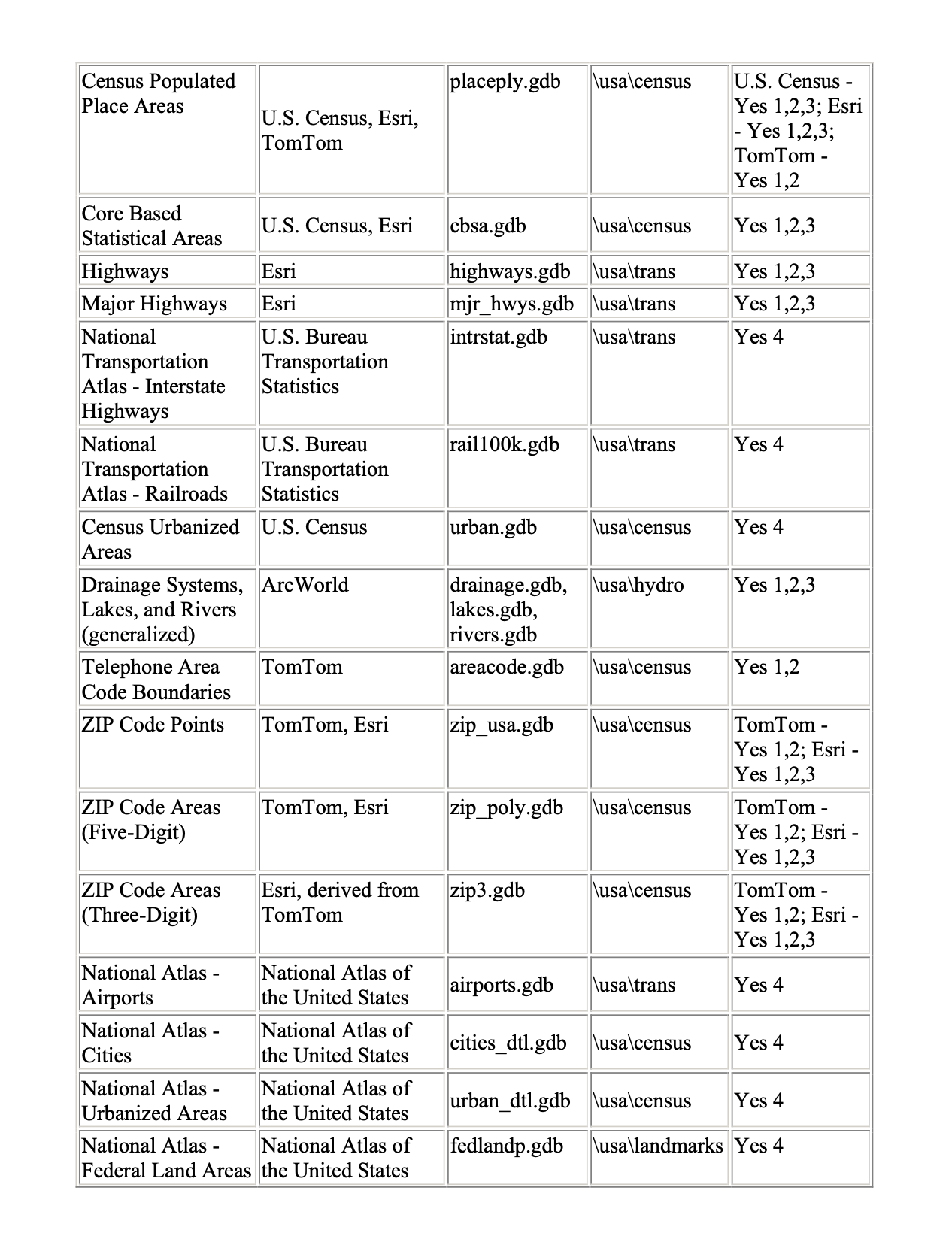


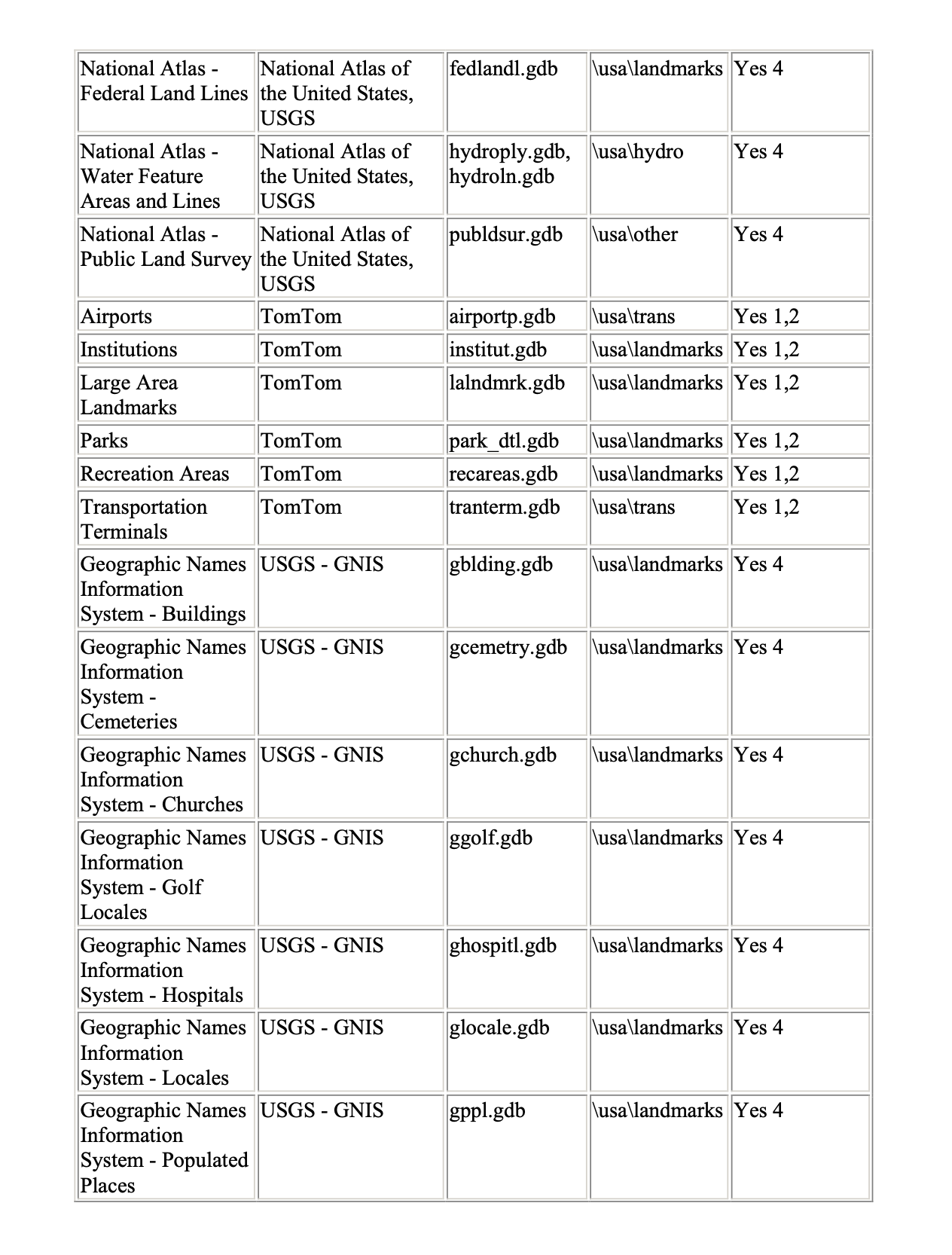


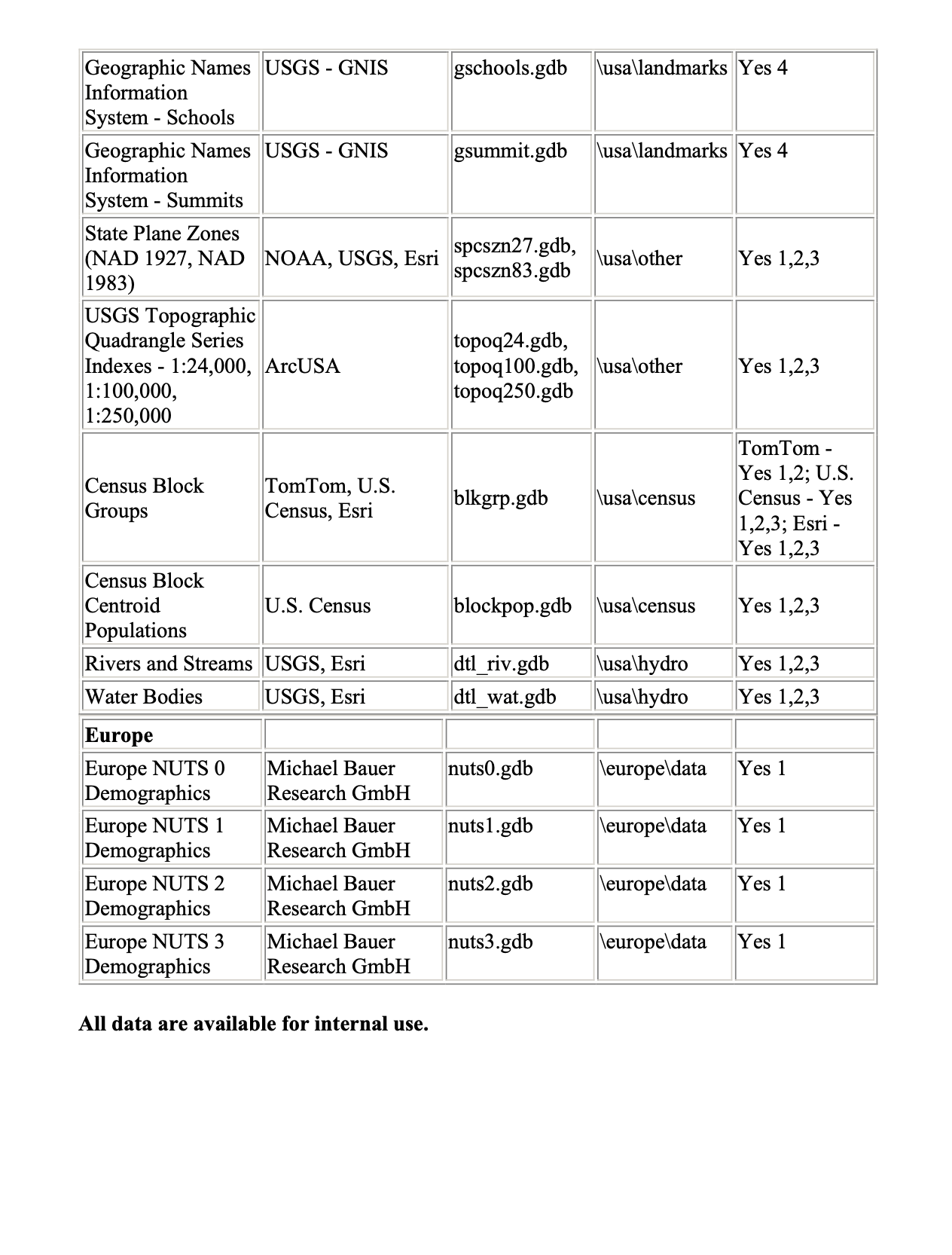


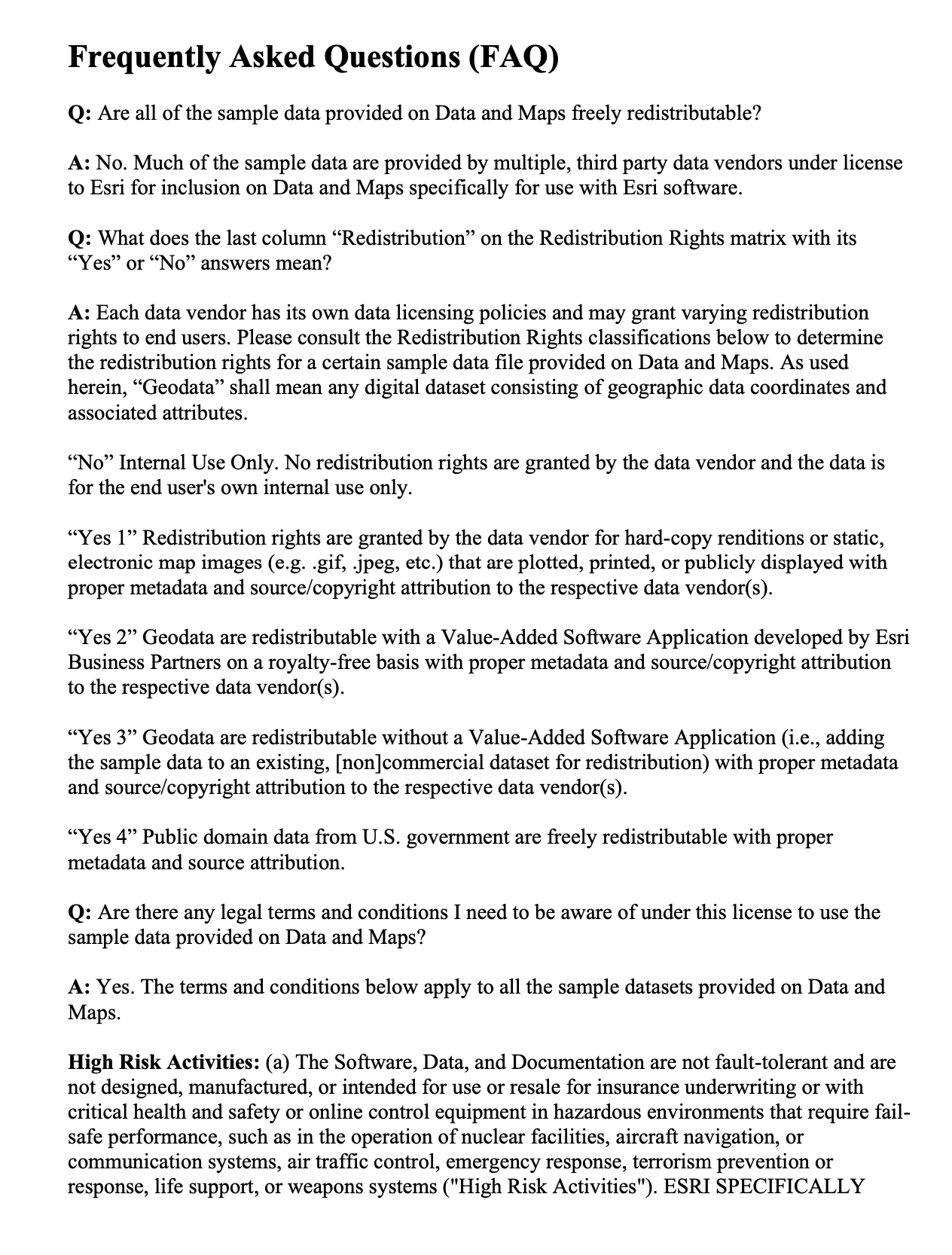


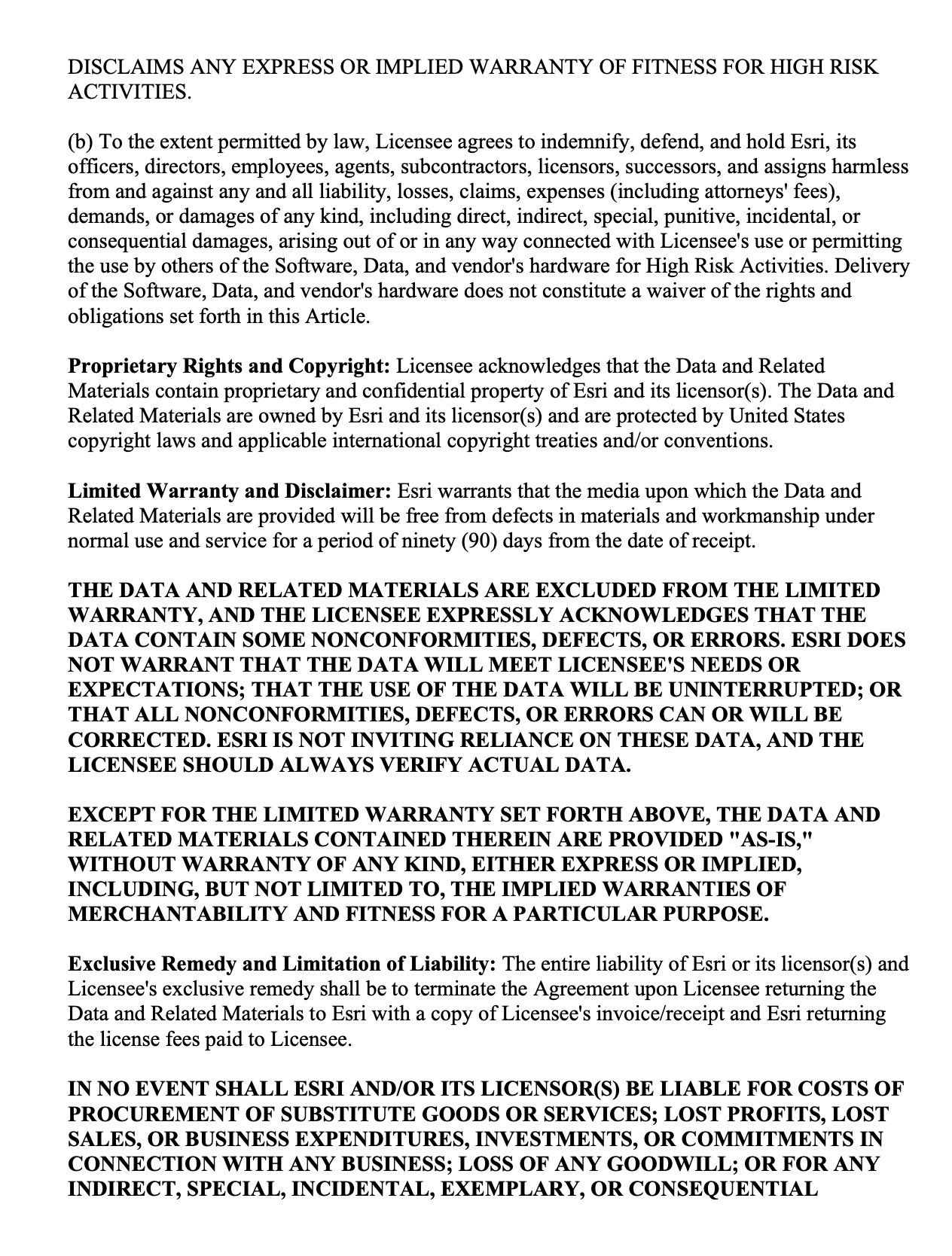


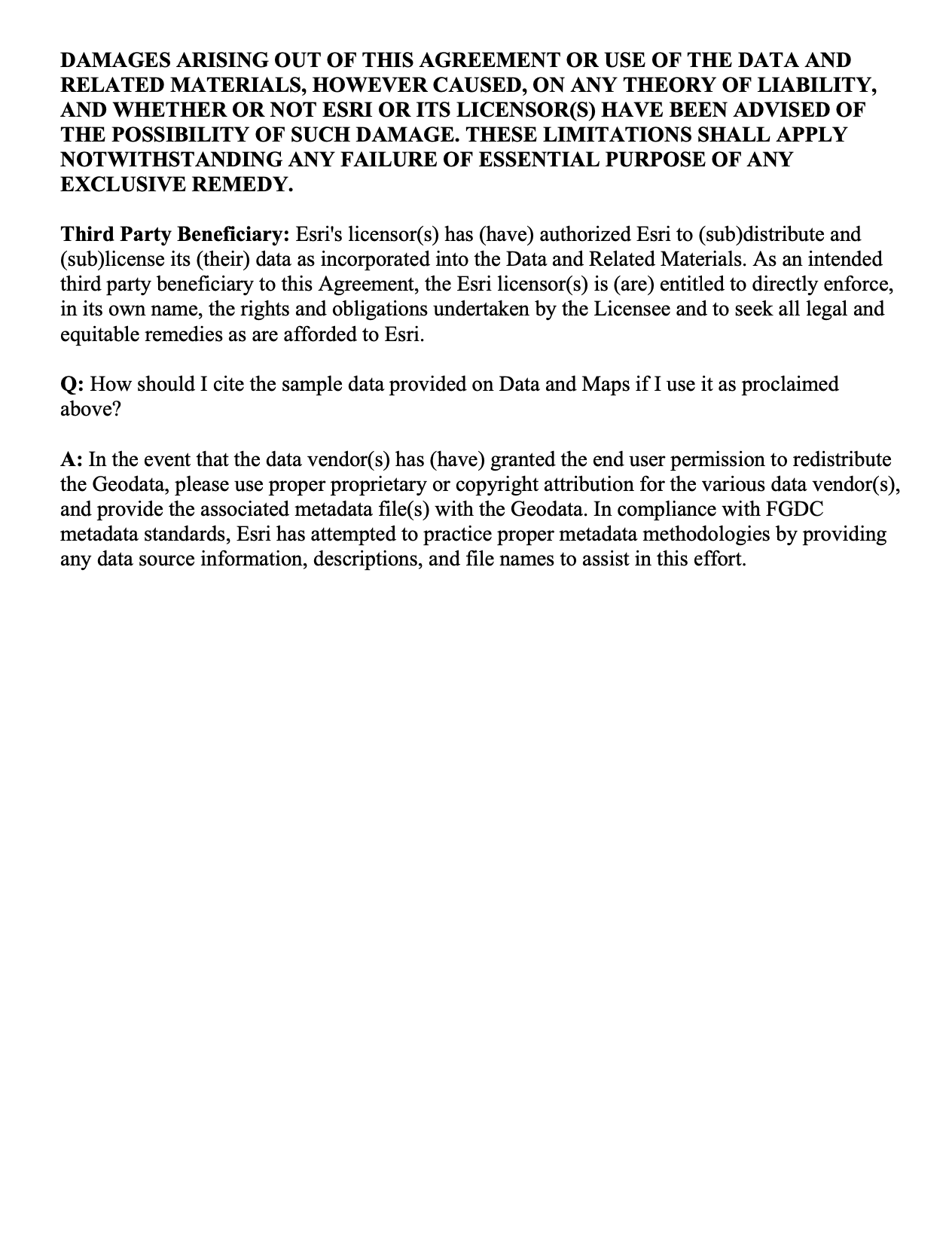

Supplement: S4 Text — (DOCX) [file ppat.1009079.s017.docx]
