# Supplementary material for: Global discovery of human-infective RNA viruses: A modelling analysis
Source: PLoS Pathog. 2020 Nov 30;16(11):e1009079. doi: 10.1371/journal.ppat.1009079 (PMC7728385; doi:10.1371/journal.ppat.1009079)
Supplement: Attachment — Submitted filename: Response to Editors and Reviewers.docx [file ppat.1009079.s020.docx]

**Responses to Editors’ and Reviewers’ Comments**

**Responses to Editors:**

*1. The manuscript has significant strengths. Many of the specific points have been addressed by the reviewers. Here, I would like to summarize some overarching points, as well as reinforcing some of the reviewers' comments.*

*The manuscript is a methodological tour de force, consonant with this group's previous work. The approach is very interesting, building on this group's earlier work on rates and locations of pathogen discovery (e.g., their Ref. 11). However, as the manuscript was not submitted as a methodological paper, therefore this manuscript should explicitly discuss how the work advances current knowledge.*

**Response:** Thanks for your comments. As discussed in our cover letter and in the comments from Reviewer 1. Our work advances the current knowledge in a couple ways.

Firstly, we have used a unique database on the discovery of new human RNA viruses and a different set of explanatory factors to solve a similar question as previous studies ^1,2^ by following a similar method. The similar results on some explanatory factors such as climate and mammal species richness validate previous studies. We added more discussions on the effect of climate and mammal species on lines 355-357 and 368-374, respectively. The different results on some explanatory factors such as population and urbanization are the novel findings from our study. We explained our new findings on lines 344-352.

Secondly, previous analyses of a related question – global distributions of outbreaks of emerging infectious diseases – reported in high profile publications ^1,2^ have had to devise metrics in an attempt to account for spatial variation in discovery effort. This is difficult to do and is widely regarded as a major weakness of such studies. We avoid this problem by re-phrasing the question, explicitly including predictors related to discovery effort in our models. This leads us to identify GDP as a key driver of the spatial distribution of virus discovery. This is a useful result in its own right but also means that the contribution of other predictors can be estimated having allowed for the contribution of GDP, making our analysis considerably more robust.

Lastly, our study has the advantage that we have performed further stratified analyses (distinguishing viruses transmissible in humans or strictly zoonotic, and vector-borne or non-vector-borne) and identified differences between explanatory factors for the discovery of specific categories of viruses. These results further understanding of the spatial distribution of virus discovery for different types, and also demonstrate that such a method can be used to identify such these sorts of differences between, say strictly zoonotic and human-transmissible viruses, and vector-borne or non-vector-borne viruses. We have included this as an advantage of our study on lines 401-407.

Ref

1. Jones KE, Patel NG, Levy MA, Storeygard A, Balk D, Gittleman JL, et al. Global trends in emerging infectious diseases. Nature. 2008;451(7181):990-3.

2. Allen T, Murray KA, Zambrana-Torrelio C, Morse SS, Rondinini C, Di Marco M, et al. Global hotspots and correlates of emerging zoonotic diseases. Nature communications. 2017;8(1):1124.

*2. In addition, as Reviewer 2 notes, the distinction and overlap between results from studies of (i) pathogen discovery and (ii) pathogen emergence should be clarified. In the absence of formal analysis of reporting/publication bias, disentangling these threads becomes particularly critical. The authors themselves at times seem unsure which of these they're referring to. For example, on p. 3, ll. 47-50, the authors state, "The results of our study further understanding of the spatial distribution of human RNA virus discovery, and maps the likelihood of further discoveries across the world. By identifying where new viruses are most likely to occur in the near future the study helps identify priority areas for surveillance.", but in the Introduction, the authors note, quite astutely, "We assume virus discovery is determined by two underlying spatiotemporal patterns: the geographical distribution of viruses in nature, and the process of virus detection—a human activity." In the cover letter, the authors imply that using this complementary approach can help to correct for resource bias in identification (discovery), among other things. In that case, it should be possible to demonstrate the valued added by this approach. Some specific examples could be used here. What are the significance and interpretation of these results, and are there recommendations? If there is a close correlation of explanatory factors between studies of discovery and studies of "emergence", why is that so? Does that imply we're looking in the right places, or simply the obvious ones, or that the factors that enhance discovery coincide with those that promote emergence, such as urbanization, travel, and increasing scientific capacity in developing countries? Does the analysis suggest a better strategy for searching? With an analysis this detailed one hopes it would be possible to go beyond the descriptive and begin more critically exploring mechanisms and hypotheses.*

**Response:** We have re-phrased where we have used ‘emergence’ mistakenly in the revised manuscript. We have also re-written our discussion by adding more mechanisms and recommendations. Please see response to Reviewer 2, Part I, comment 1 for details.

*3. The figures are very elegant, but to this digital non-native, some seemed duplicative, and seemed to obscure the main points. The text itself is rather dense. For the readership of PLoS Pathogens, I think more explanation and context are required. Similarly, the Discussion* *should more explicitly discuss any unique benefits and added value of this approach. Again, an example or a few examples of how this can be used to advance our knowledge would be invaluable. Earlier analyses appear to have arrived at similar conclusions from a different vantage point so ways to use the analyses in the manuscript to extend this knowledge beyond the descriptive would be very useful. The analysis does study trends over time (including a video), and that would seem to merit more discussion as well.*

*This is admittedly a difficult subject. The authors' group has a developed excellent database that they have generously and most commendably shared freely with all interested researchers. As they indicate, however, the data are still sparse and rife with questions of definition. Nevertheless, it remains a useful resource, and I hope that this manuscript can advance understanding of the mechanism of the emergence process. The Discussion seems somewhat limited, and I would encourage the authors to consider additional conclusions and implications of their work in their discussion.*

**Response:** We agree some figures look duplicative, and we have removed quite a few figures in the revised manuscript (see response to Reviewer 2, Part II, comment 2 for details). In the result, we have added more description for S1 video on the trends over time, definition for some land use factors and the legend for figures to allow readers to understand more easily.

Again, we have improved our discussion by adding more mechanism underlying the identified explanatory factors, such as GDP, GDP growth, urbanization and mammal species richness and proposed recommendation for future surveillance. We also re-written our conclusion with some additional conclusions.

**Responses to Reviewer #1:**

***Part I - Summary***
***Please use this section to discuss strengths/weaknesses of study, novelty/significance, general execution and scholarship.***

*1. This paper uses a number of techniques, most significantly an ensemble of boosted regression trees (BRT)-based approach, to find associations between a set of predictor variables and their outcome variable representing RNA viral discovery.*

*The paper appears to follow methods and workflows strongly derived from Allen et. al. (2017) (currently citation 13), particularly in the generation of its BRT models and associated figures. I would suggest that the Methods section include a statement like, “We followed methods and used code derived from Allen et al (2017)…,” where describing derived methods/code/workflows.*

**Response:** Thank you for the suggestion. We have followed the methods and workflows derived from Allen et. al. (2017) (though we have changed some like use of the Poisson BRT model not the Bernoulli, and therefore the validation statistics have changed from AUC and TSS to ICC) and we appreciated the wonderful work Allen et. al have done and the generosity for sharing their code online. We added the statement in the Methods overview that ‘In this study, we followed methods and used code derived from Allen et al’.

*2. In addition, the paper should share the code used to generate it’ models, as per the recommendations in PLOS’s Materials and Software Sharing policies (https://journals.plos.org/plospathogens/s/materials-and-software-sharing). Sharing model and figure code removes any ambiguity that may be present in the natural language description of a study’s methods as a result of space constraints or the difficulty of describing complex processes in natural language. Code should preferably be shared with an appropriate license on GitHub and assigned a DOI using a service such as Zenodo, but could also be included with the study’s Supplementary Information, as its dataset already is.*

**Response:** We agree that sharing code for model and figures would help readers better understand the study and removes any ambiguity. We have now included our raw data and R code for the full model in S1 R script.

*3. Following a similar process but with a separate outcome dataset with different inclusion criteria and a number of different predictors, the paper comes to similar conclusions as other studies on the same topic (particularly references 12 and 13) for its main model. That the different datasets would yield finds similar results undergirds the conclusions of the earlier papers. Some of their high-influence variables are similar (e.g. mammal species richness) and others are divergent (e.g. finding an association and significant impact for climate variables). These differences between similar previous studies are worth publishing.*

*This study also runs different BRT models for biologically distinct subsets of RNA viruses, and the finds notable differences between the drivers of these models. These findings are notable because they describe differences in the global distribution of viral discovery for different types of viruses, and second because they demonstrate that such a method can be used to identify such these sorts of differences between, say, zoonotic-only and human-transmissible diseases.*

*It’s my opinion that these contributions — the influence of climate variables and the stratified analyses — are the biggest points of departure from earlier literature, and this this studies best contributions.*

*There are a few other points that could be improved in the study’s discussion of its findings. These are noted below.*

**Response:** Thank you for the encouraging comments.

***Part II – Major Issues: Key Experiments Required for Acceptance
Please use this section to detail the key new experiments or modifications of existing experiments that should be absolutely required to validate study conclusions.***

***Generally, there should be no more than 3 such required experiments or major modifications for a "Major Revision" recommendation. If more than 3 experiments are necessary to validate the study conclusions, then you are encouraged to recommend "Reject".***

*There are no major new experiments required for publication.*

***Part III – Minor Issues: Editorial and Data Presentation Modifications
Please use this section for editorial suggestions as well as relatively minor modifications of existing data that would enhance clarity.***

*1.On line 49, the authors state that they identify “where new viruses are most likely to occur”, but they are actually identifying where they are most likely to be discovered. This might seem like splitting hairs, but since the authors did not treat the effect of reporting bias separately, it’s an important distinction to make.*

**Response:** Yes, we agree we made a minor mistake here – we have changed our statement on line 49 (now on line 51) to “where new viruses are most likely to be discovered”.

*2. The authors’ discussion of this very phenomenon on lines 74–83 is cogent, and succinct. Distinguishing between virus species range and discovery probability is one of the clearest explanations of this subject I’ve read.*

*However, their methodological treatment of reporting bias is not clearly described here or in the methods section. In the introduction, it is described on lines 84-86: “Here, we take a different approach by identifying explanatory factors of the raw virus discovery data and then considering whether these relate to virus geographic range or discovery effort or both.” This might be made clearer by saying something like “and then interpreting in the results whether these effects might relate to virus geographic range or discovery or both.*

**Response:** Thank you for the encouraging comments and we have changed the statement to “and then interpreting in the discussion whether these effects might relate to virus geographic range or discovery or both” on lines 84-86 (now on lines 89-91) in the introduction. We also added a sentence on lines 133-135 in the methods section that ‘We expect GDP, GDP growth and university count etc. to be correlated with discovery effort as they imply more resources that could be invested in virus research’ to better describe our methodological treatment of reporting bias.

*3. The Discussion section notes that the paper does not attempt to explicitly correct for reporting bias (line 361). In this mention, they cite Jones et al. (ref. 12) but not the subsequent paper (ref. 13) which was an improvement on the Jones et al. method. However, the study includes variables expected to be associated with discovery effort, e.g. GDP and university count. Earlier in the discussion, it is noted that GDP growth is among the top predictors, and its effect differs across the stratified models. That the study does not attempt to factor out reporting effort means that its authors should be careful to interpret their results only as “viral discovery” and not “viral emergence”.*

**Response:** Sorry we missed to cite the paper of Allen et al (2017) on line 361 (now on line 395) and we have now added it now. Emergence is hard to define and discovery always occurred after emergence, and discovery is the best description of our database. We have tried our best to use the location of the first infected human to infer the likely origin of each virus because this is more relevant to the public health, but we acknowledged we focus on the “viral discovery” in this paper and not “viral emergence”.

*4. The paper should be given a minor copy edit (e.g. “spatial dependencies” -> “spatially dependent” on line 93).*

**Response:** We have changed “spatial dependencies” to “spatially dependent” on line 97 (now on line 150) as you suggested.

*5. The contribution of the paper’s use of k-means clustering to its central research questions is unclear. The paper’s initial discussion of the k-means clustering analysis suggests that this was perhaps used as selection criteria for including points in the model (line 92, “if the spherical K function detected a clustered pattern, a Poisson boosted…”). However, the flow chart in the Supplementary Information gives the impression that the clustering analysis was not part of the BRT modeling workflow, but a separate analysis. If the k-means analysis is part of the modeling workflow, its relation to the BRT models should be made more clear. If it is a separate analysis, the authors should devote space in the Discussion section to interpreting its outcome (the figure in the supplementary information is not self-explanatory) or remove it from the paper entirely.*

**Response:** K-means clustering was part of the BRT modelling workflow, though we failed to show this clearly in the supplementary information and so we have removed the workflow figure from the paper. In fact, BRT model can deal with both spatially dependent data and normal data so we feel we can just use the BRT modelling directly and there is no need to introduce the spherical K means here (as you have noticed we calculated Moran’s I for both the raw virus data and the model residuals already) and so we also removed the part of spherical K means clustering totally from the paper.

*6. Line 167: For readers unfamiliar with BRTs, perhaps move the sentence about partial dependence plots to a new paragraph, or introduce it in a slightly different way, something like, “Partial dependence plots are a method of visualizing the relationships between a BRT’s predictive variables and its outcome…”.*

**Response:** We have changed the description for partial dependence plots on line 167 (now on line 175) as you suggested.

*7. Line 199: The paper talks about making predictions for 2015-2024, but these predictions are in fact the model’s output for 2015 variables. The authors do not go into enough detail about how the variables were matched to decade for us to assess this. If the outcome of the model uses 2015 variables because these are the most recent ones available, the model’s conclusions are still valid, but stating those predictions as “2015-2024” without justification would be misleading.*

**Response:** Initially, we have used 2015 values for variables as a proxy for 2020 values to make predictions for 2015-2024 because these are the most recent ones available when we started the analysis in earlier 2018. But now we realised that we’d better say we have made predictions for 2010-2019 using 2015 values as we always matched the middle values of variables for each decade with viruses detected in that decade. For example, 2010 values of variables were matched with viruses discovered in 2005-2014 (we added this example to the main paper one lines 164-165 to better explained how we matched virus data with time varying explanatory factors). We have changed ‘2015-2024’ to ‘2010-2019’ throughout the paper.

*8. Line 231: The use of Moran’s I to demonstrate the model’s removal of spatial residuals is very clear.*

**Response:** Thanks for the encouraging comments.

*9. Figures 3 and 4 uses the labels “Relative Contribution” and “Relative Influence” inconsistently. It should use only one. Readability would be much improved if the subplots were titled, rather than just being labeled A and B, so the reader can see which group they represent rather than referring to the caption.*

**Response:** Sorry about our mistakes. We have changed “Relative Influence” to “Relative contribution” in Fig 3 and Fig 4. We also added titles for the subplots in these two figures and S4 Fig and S5 Fig as you suggested.

*10. The maps use a color palette which is not perceptually uniform — a color palette such as Viridis or Google’s Turbo color palette would be better for displaying this kind of continuous data on a map.*

**Response:** Thanks for your suggestion - we have now changed to Viridis color palette for Figure 6, S6 Fig, S7 Fig and S8 Fig.

*11. Line 365: The high-resolution variables were all scaled down to a 1º grid, so the higher resolution of the source variables was not a factor in the models’ output.*

**Response:** We agree with your comments and we have removed this from our advantages. We aimed to use data with higher resolution but are limited by my computer capability.

**Responses to Reviewer #2:**

***Part I - Summary***
***Please use this section to discuss strengths/weaknesses of study, novelty/significance, general execution and scholarship.***

*1. There are many strengths to this study, including their recognition of the need to deal with the critical issue of variation in research effort.*

*However, throughout the manuscript I found myself challenged by what ‘virus discovery’ really means in the context of this study. I was often confused about whether the authors are truly focused on the factors that influence virus discovery, or if they are actually focused on factors that influence virus emergence. Indeed, the opening two sentences of the abstract reflect this unresolved conflict between predictors of emergence (“…the prediction of where new RNA viruses will emerge is a significant public health concern”) and virus discovery (“we matched these data….to predict the probability of virus discovery”). While this might seem like semantics, I think it is critical to identify and articulate specifically what the paper is trying to achieve. Discovery and emergence are not the same process, and thus presumably should be approached differently in study design and interpretation. There are many many examples throughout the text where the two processes are confused and, unfortunately, this limited my ability to evaluate the specific contribution of this study.*

**Response:** Thanks for your comments. We focus on the factors that influence virus discovery, rather than emergence. This is determined by the attribute of the database itself, i.e. the first report of each human RNA virus from the literature review. We can hardly know when and where each virus emerged, but we can know when and where they are discovered from published literature. Discovery always come after emergence, and the discovery location location may represent the origin of the virus, but also may not if the virus can spread and get reported from a local patient in a relative developed region. We define ‘discovery’ not to mean the location of the laboratories in which they were studied (except in a minority of cases where no other data were available), but the place where infected human(s) were first identified (see below) – this is a more of public health concern.

Therefore, we have revised our paper where we have mistakenly used ‘emergence’, e.g. on line 23, ‘where new RNA viruses will emerge’ was changed to ‘where new RNA viruses are likely to be discovered’. We also added one paragraph on lines 311-317 in the discussion why we have focused on discovery rather than emergence.

*2. I kept going back to the example of Reston virus, which was discovered in Virginia (U.S), but geocoded to Manilla, Philippines. If the study is about understanding factors underlying virus discovery, it seems odd to ascribe the discovery of Reston virus to Manilla. Reston virus was discovered because infected animals happened to end up in the hands of an ebolavirus expert at a high-containment lab, in a completely different country. I can understand why the authors decided to code Reston virus to the Philippines (it is where the virus naturally circulates); however, I question whether these spatial covariates are relevant to its discovery in the U.S. In this case, I think the spatial data that is linked to the Philippines would tell us more about virus transmission and emergence than discovery, and I worry about spurious associations. The same issue exists for Ebola virus (species Zaire ebolavirus), which was ascribed to DRC when it was actually discovered by Prof Piot in Belgium.*

*Ignoring, for a moment, whether these viruses are appropriately geocoded for their particular question, there is another problem. That is, the approach taken to geocode the viruses is inconsistent. For example, while Reston virus and Ebola virus were coded to reflect their point of origin (i.e., where the virus was naturally circulating), other viruses like influenza were coded to reflect where they were indeed first ‘discovered’. Influenza was first characterized in 1933 in Mill Hill, London, and the authors have geocoded it accordingly. The approach taken in coding influenza therefore appears to be different from the approach taken for Reston and Ebola. While the spatial covariates for Reston virus and Ebola virus are capturing one type of information (location of original circulation), the covariates for other viruses like Influenza or Hepatitis C are capturing a different type of information (location where the virus was actually discovered, but not necessarily or exclusively where it was circulating).*

**Response:** As we mentioned on lines 117-124, we defined “discovery” as the first report of an ICTV-recognised RNA virus species from human(s) in the peer-reviewed literature, and the location of initial human exposure/infection with the virus was taken as the discovery location. In our database, human(s)’ addresses were used for 223-3-19=201 (90%) viruses, and we have performed the sensitivity analysis by removing the 22 discovery reports that were not human(s)’ locations (as these are less precise), and revealed consistent trends with the full model, though with several changes of relative contribution.

We acknowledged it’s difficult to confirm which is the initial description of human infection of one certain virus, but the paper we used as discovery for Reston virus described seropositive individuals in Manila, and was the first paper describing human infection to meet our criteria for robust evidence ^1,2^. Similarly, we selected DRC as the discovery location for species Zaire ebolavirus because the samples were taken from patients from Zaire, though scientists from Belgium, England and the USA detected and published this virus on the same issue of Lancet in 1977.

While capturing emergence would be more relevant to public health, it would also be much more challenging and data-deficient because most viruses have circulated in humans for many years before people discovered and published them. However, even though we cannot know exactly where and when emergence took place, we always know by this for discovery by definition, and emergence must always precede discovery, which motivated our approach.

For some widely distributed viruses like Influenza or Hepatitis C, it was always those developed countries firstly discovered them, but no one clearly knows where these viruses were originally from.

Ref

1. Miranda ME, White ME, Dayrit MM, Hayes CG, Ksiazek TG, Burans JP. Seroepidemiological study of filovirus related to Ebola in the Philippines. *Lancet* 1991; **337**(8738): 425–6.

2. Woolhouse MEJ, Brierley L. Epidemiological characteristics of human-infective RNA viruses. *Scientific data* 2018; **5**: 180017.

*3. Throughout the paper, I worry that ‘discovery’ and ‘emergence’ have been confused. In the author summary, they state “By identifying where new viruses are most likely to occur in the future, the study helps identify priority areas for surveillance.” The phrase “most likely to occur” suggests they are indeed interested in emergence. Equally, in the discussion they state “This implies that it is the change in habitat—from natural or rural to urban—has a greater influence on virus discovery than human population size or density.” Again, this sentence implies that the study is about emergence, not discovery. However, elsewhere in the text they refer specifically to need to “identify the factors driving the discovery of RNA viruses”. I think it would be important to more clearly articulate exactly what the study is focused on (I think this is virus discovery) and comment on how this links or informs on our understanding of viral emergence (if indeed it does). Currently, I am not sure how to interpret the following statement in their abstract: “…areas with the highest predicted probability for 2015–2024 include new foci in East and Southeast Asia, India, and Central America”. Does this really mean areas where new viruses are likely to be discovered? Or does it mean areas where new viruses are likely to emerge, and then subsequently discovered simply because they are emerging?*

**Response:** Thanks for your comments – again, we have revised our paper where we have mistakenly used ‘emergence’.

On lines 337-338 in the discussion, we stated that in contrast to GDP, all other explanatory factors identified in this study appear more directly associated with virus geographic distributions. It seems that the change of habitat doesn’t directly influence discovery, but here in fact we meant it would influence the virus geographic distributions in nature which then results in discovery (with the statement added on line 351).

On line 34 in the abstract, the newly identified areas include both areas with a hard discovery effort and areas where viruses naturally existed. We changed ‘new foci’ to ‘three new regions including East and Southeast Asia, India, and Central America, which likely reflect both increasing surveillance and diversity of their virome’.

*4. I also found the definition of ‘discovery’ confusing. It was defined as ‘the first isolation of a virus in a human patient.’ Given that many human viruses were first isolated years after they were actually discovered, it would be good to clarify whether the authors really do mean ‘isolated’. (To a virologist, this means culture of the virus in vitro or vivo). In reviewing the supplemental material, I see that the authors have used 1989 for hepatitis C. This makes me think they do not actually mean ‘isolated’, but rather ‘discovered’. Hep C was first cloned in 1989, but it was not successfully grown in culture (‘isolated’) until around 2005. Again, this is inconsistent with examples like influenza. Note, I did not review the list of viruses exhaustively, so would recommend that the authors double-check everything. In addition, the term ‘patient’ is included in their definition and is perhaps misleading. Many of the viruses they have included are probably not human pathogens, or at least have not been conclusively linked with human pathology. Reston virus is a good example of this.*

**Response:** Sorry, our statement here was improper - we meant the first report of evidence of the specific virus from human(s). We have changed our definition on line 117 accordingly. Our published database^1^ indicated that both virus isolation and serological methods (but not symptom reports) were accepted evidence for human infections of a virus. We have gone through our database again and confirmed evidence of human infection is robust for each virus.

Ref

1. Woolhouse MEJ, Brierley L. Epidemiological characteristics of human-infective RNA viruses. *Scientific data* 2018; **5**: 180017.

***Part II – Major Issues: Key Experiments Required for Acceptance
Please use this section to detail the key new experiments or modifications of existing experiments that should be absolutely required to validate study conclusions.***

***Generally, there should be no more than 3 such required experiments or major modifications for a "Major Revision" recommendation. If more than 3 experiments are necessary to validate the study conclusions, then you are encouraged to recommend "Reject".***

*(No Response)*

***Part III – Minor Issues: Editorial and Data Presentation Modifications
Please use this section for editorial suggestions as well as relatively minor modifications of existing data that would enhance clarity.***

*1. The authors considered a combination of socio-economic, land use, climate, and biodiversity variables as correlates of virus discovery. What is the rationale for how/why they are relevant to virus discovery? And given that land use, climate, and biodiversity variables seem more associated with emergence, can they provide some discussion on why these were selected, beyond saying ‘it’s a way to deal with variation in research effort’.*

**Response:** We added in the methods on lines 133-137 why we select these variables. GDP, GDP growth and university count etc. are expected to be correlated with discovery effort as they imply more resources that could be invested in virus research. Other groups of variables including land use, climate, and biodiversity are more likely to be related to the natural geographic range of the virus, and affect discovery via the intermediate step of emergence.

*2. For their future predictions – did they account for projected changes in the factors that were correlated? Forecasted changes in GDP, land use, climate, etc.*

**Response:** No, we didn’t, and we just used 2015 values for GDP, population, land use, and climate. There are predicted gridded data sets for climatic variables ^1^ and land use ^2^ in 2020-2100, based on different scenarios (RCP 2.6, RCP 4.5, RCP 6.0, and RCP 8.5), but we didn’t find gridded data sets for population and GDP with matched scenarios.

Ref.

1. Swart, N. C., Cole, J. N. S., Kharin, V. V., Lazare, M., Scinocca, J. F., Gillett, N. P., Anstey, J., Arora, V., Christian, J. R., Jiao, Y., Lee, W. G., Majaess, F., Saenko, O. A., Seiler, C., Seinen, C., Shao, A., Solheim, L., von Salzen, K., Yang, D., Winter, B., and Sigmond, M.: CCCma CanESM5 model output prepared for CMIP6 CMIP, Earth System Grid Federation, available at: https://doi.org/10.22033/ESGF/CMIP6.3673, 2019b.

2. Chini LP, Hurtt GC, Frolking S. Harmonized Global Land Use for Years 1500 -2100, V1: ORNL Distributed Active Archive Center, 2014.

*3. Line 61 – “Human RNA viruses”. Can they authors define what they mean by a human RNA virus? Is it a virus that was found in a human at least once? And if so, is that sufficient to call it a human virus?*

**Response:** Yes, human RNA virus meant a virus was found in humans at least once. As defined in the earlier paper^1^, both natural infections and unintentional iatrogenic or artificially-acquired infections were considered as evidence supporting human infection, and intentional infections and in vitro infections were excluded. Acceptance of viruses as human-infective was primarily based on expert review, where the following were systematically taken into consideration: diagnostic method(s) used to identify human infection, number of reported infections, number of authors independently reporting infection, author confidence in identity and infectivity of virus. There are 33 viruses which have been reported only once, with 13 viruses reported from one single person. We agree this maybe not sufficient to call it a human virus therefore we have performed a sensitivity analysis by removing these 33 viruses and get roughly consistent result as the main result, i.e. similar relative contribution rankings of variables and similar hotspots.

Ref

1. Woolhouse MEJ, Brierley L. Epidemiological characteristics of human-infective RNA viruses. *Scientific data* 2018; **5**: 180017.

*4. Lines 62-64: Species don’t circulate; viruses do. Please correct taxonomy and use virus names (not species names) when talking about viruses as nouns. No italics.*

**Response:** Thanks for your suggestion. We have edited the sentence on lines 62-64 (now lines 64-67) as you suggested.

*5. Line 66-67: “…some have been identified during active viral discovery programmes”. Please provide citations as examples. Also consider adding a sentence to say that some viruses have been discovered by chance, as incidental findings as part of a disease investigation. (I.e., a virus was identified in a sick person, but the virus was not thought to be the cause of the disease. Just an incidental finding).*

**Response:** We have provided citations and added the sentence on lines 70-71.

*6. Lines 109-111. I realize that this is likely not a fair thing to ask, but given the impact of the current pandemic, it seems that including SARS-CoV-2 would be good?*

**Response:** We can’t include SARS-CoV-2, though we also wanted to. The ICTV *Coronaviridae* Study Group (ICTV-CSG) determined that SARS-CoV-2 belongs to the existing species, *Severe acute respiratory syndrome-related coronavirus*^1^, which we have included in our study already.

Ref

1. Gorbalenya, A.E., Baker, S.C., Baric, R.S. *et al.* The species *Severe acute respiratory syndrome-related coronavirus*: classifying 2019-nCoV and naming it SARS-CoV-2. *Nat Microbiol* **5,** 536–544 (2020). https://doi.org/10.1038/s41564-020-0695-z

*7. I would appreciate some justification for the taxonomic boundaries they have used, or at least some discussion about the potential limitations of this approach. While it might seem robust to use ICTV classifications – species demarcations vary widely by virus family. The factors used to demarcate species in one family can be quite different from the factors used to demarcate species in another family. I recognize that there is clearly no good way to address this problem, just as there is no clear answer for how to deal with variation in research effort. However, like the research effort problem, this has the potential to significantly impact their results. For example – is it reasonable to consider all influenza viruses as one example while separating out enteroviruses into so many species?*

**Response:** Thanks for the valuable suggestion. We have included this as a limitation of the study on lines 389-393.

*8. Figure 6. Do these hotspots indicate where good lab capacity exists? I am trying to understand the specific significance of this figure. What does it really mean and how would I use it to guide surveillance?*

**Response:** The hotspots indicate either places with good detection capacity (noting that this is a product of capacities beyond laboratories, e.g. field surveillance, hospital infrastructure) and/or places with more new viruses in nature. We should focus those hotspots in less-developed areas where land use, climate and biodiversity are the main factor for virus discovery there but not GDP (with the implications added on lines 414-417).

**Responses to Reviewer #3:**

***Part I - Summary***
***Please use this section to discuss strengths/weaknesses of study, novelty/significance, general execution and scholarship.***

*This paper is a timely advance for understanding discovery of human RNA viruses that will be of interest to researchers working on emerging infectious disease. The methods are rigorous and this work uses a new approach to evaluate patterns in virus detections. This work highlights important correlates of newly recognized RNA viruses that can help predict future emergence. The insights are not especially novel but findings are important and confirm findings previously reported using new data and methods. The only major concern is that the very broad presentation of correlates in a model limits the impact and relevance of this work.*

**Response:** Thanks for the encouraging comments. Virus discovery is a so complicated process that many factors may have been involved in. Variable selection always hurts, and the boosted regression tree (BRT) model we used in the paper have the capability to capture complex relationships and interactions between variables and to rank the relative contribution of these variables. We have also categorised these variables into different groups to get them interpreted easier.

***Part II – Major Issues: Key Experiments Required for Acceptance
Please use this section to detail the key new experiments or modifications of existing experiments that should be absolutely required to validate study conclusions.***

***Generally, there should be no more than 3 such required experiments or major modifications for a "Major Revision" recommendation. If more than 3 experiments are necessary to validate the study conclusions, then you are encouraged to recommend "Reject".***

*1. The analyses are thorough and evaluate a wide range of potential correlates for virus discovery. But a major limitation of this manuscript is that the inferences are not clear beyond very broad assessment of relative contributions for factors evaluated that are presented in numerous figures in the main text and SI. Concrete and meaningful functional relationships between key predictors and the outcome are not derived or explained in the results and discussion. Exploration of specific values for predictive factors and how these influence quantity of virus discovery would bring meaning to these findings. For example, the authors should explore how urbanization influences virus discovery – what levels of urbanization were more influential and what does this mean for virus discovery based on expected future trends in urbanization? Examples to highlight virus discoveries and the factors related to their first detection that underlie broad patterns in the data would bring clarity and meaning to findings.*

**Response:** We have described how the risk of virus discovery changes as a function of our variables on line 241-244, and added potential mechanistic explanations in the discussion (e.g. on line 324-336).

*2. The many figures in the main paper and SI are largely of the same variety of relative contributions to the model and probability maps – these should be narrowed down to only those that highlight major findings with improved quality. A figure that shows specific values of the one or two key factors and the predicted influence on number of viruses discovered would enhance the presentation of results.*

**Response:** Thanks for your suggestion. We have now cut down our figures by removing figures for sensitivity analyses, because they have provided the similar information with the full model. We also deleted the workflow chart following other reviewers’ advice. The spherical K function was deleted because it seems to be duplicative with Moran’s I we have calculated. We deleted the figure showing virus count by country because it did not add much valuable information in comparison with Figure 1 in the main paper.

We agree a figure that shows specific values of the one or two key factors and the predicted influence on number of viruses discovered would enhance the presentation of results, but a figure like that will provide duplicative information with Fig 6 in the main paper and the S6 Fig in the SI. Readers can compare themselves freely. In the discussion, we have mentioned what levels for the key factors that are more influential on virus discovery.

*3. Can the authors further explore the rate of detection over time? How did rate of detection or the temporal distribution vary by the 4 classifications of viruses; did the rate of detection vary by geographic region? The influence of the predictors on prediction is the most novel aspect of this work yet it is not explored beyond highlighting broad regions with increased probability. Can the authors add specific inferences based on the analysis in terms of the number of viruses expected to be discovered every year, by virus groups examined? Is it possible to predict this beyond 2024?*

**Response:** As Fig 1 in the main paper, we have added a timeline of the virus discovery for the 4 classifications of viruses at the bottom-left corner in S1 Fig, and added description of the temporal distribution of each category in the results (on lines 224-228).

We also added more description for the S1 Video, i.e. the rate of detection by geographic region (on lines 221-223). There is variation for the rate of detection (as shown in R1 Fig below).

**R1 Fig**. Virus species count split by continent over time

We can’t infer the number of viruses expected to be discovered for each year (neither for the four classifications). We can only predict the number of viruses by decade – that’s determined by the method we fitted the model. Our model matched the RNA virus discovery count in each grid cell with historical decadal climatic variables, population, GDP, and land use data. For example, 2010 values of variables were matched with viruses discovered in 2005-2014. Therefore, we can only predict decadal number of viruses discovered. We are also unable to predict this beyond 2024. As how we respond to Reviewer 2 in Part III, comment 2, we didn’t find gridded data sets for population and GDP with matched scenarios with climatic variables and land use.

***Part III – Minor Issues: Editorial and Data Presentation Modifications
Please use this section for editorial suggestions as well as relatively minor modifications of existing data that would enhance clarity.***

*1. Abstract should include mention of investigative effort specifically (the process of virus detection) as a driver of discovery, as highlighted in the paper.*

**Response:** We mentioned this on line 25 by changing ‘33 covariates’ to ‘33 explanatory factors related to natural virus distribution and research effort’.

*2. Given there is overlap between zoonotic viruses, human transmissible viruses, and vector-borne viruses, this overlap should be clarified in the text, by showing % overlap among categories, ie for human transmissible viruses, what % are zoonotic.*

**Response:** We clarified this on line 341 that 78·5% (73 out of 93) of strictly zoonotic viruses are vector-borne.

*3. Information on how the geocoded location for virus detection was ascertained should be described at least briefly in the main paper, as opposed to only in the SI. When describing patient’s address, was this the location of the hospital or clinic, site of initial human exposure/infection with the virus? Were discoveries of virus in people that likely contacted the virus while travelling included? If so, how would this affect the findings?*

**Response:** We have moved some information on how the geocoded location for virus detection was ascertained from the SI to the main paper on lines 117-126. None of these viruses were contracted while travelling. See the main paper for details.

*4. The methods for data collection should be fully characterized, including search terms, databases searched, and inclusion or exclusion criteria, to enable repeatability. This information does not appear to be readily available in the link provided.*

**Response:** The link we provided is only for the database. The reference^1^ we cited in the main paper provided details for search terms, databases searched, and inclusion or exclusion criteria (with this sentence added on line 110).

Ref

1. Woolhouse MEJ, Brierley L. Epidemiological characteristics of human-infective RNA viruses. Scientific data 2018; **5**: 180017.

*5. Line 224 - The partial dependence plots do not provide detailed descriptions of the relationships. This should be rephrased. For all figures of partial dependence plots, the x axes should be labelled. Many of these variables (eg primary land, secondary land, urbanization of primary land) are not readily understood unless the SI table is reviewed. Brief descriptions within the main text would ensure improved understanding of the main findings.*

**Response:** We have changed the sentence on line 224 (now on line 239) to **‘**The partial dependence plots shown in S2 Fig showed the relationships between these explanatory factors and virus discovery’. For all figures of partial dependence plots, we have added the units for each explanatory factor. We have also added brief descriptions on some variables such as secondary land and urbanization of cropland. Primary land was not identified as an important variable so we didn’t explain this in the main text.

*6. The relationship between GDP and virus discovery should be better explained, especially with respect to how the GDP of the country of virus detection and GDP change is related to detection. Is the GDP of a country where a virus was first detected generally reflect effort or resources spent toward investigative efforts at that location? Share evidence to support interpretation. Brazil, Nigeria, and Uganda are areas with high virus detection – how does GDP explain these detections and what were the specific factors involved in virus discovery; was this evenly distributed in time or based on unique efforts?*

**Response:** We added more explanation for the relationship between GDP and virus discovery as you suggested on lines 324-336. We agree with the statement that the GDP of a country where a virus was first detected generally reflect effort or resources spent toward investigative efforts at that location. Comparison of Fig 1 with S6 Fig suggested that more viruses have been discovered in developed regions with/without fast GDP growth including North America, Europe, and eastern Australia. We note that more developed countries are more likely to first capture viruses circulating in multiple regions. Over the last 100 years, North America and Europe have witnessed a decreasing fraction of discovered viruses in more recent decades (1985–2018: 32/86=37%) than previously (1901–1984: 78/137=57%), but Asia has accounted for a higher fraction (1901–1984: 16/137=12%; 1985–2018: 22/86=26%) (S1 Video). This can be partly explained by the higher GDP and faster GDP growth in Asia in recent decades. In addition, there have also been historical hotspots in individual countries (e.g. Brazil, Nigeria and Uganda) associated with active virus discovery initiatives such as those supported by the Rockefeller Foundation.

*7. Lines 327-331 – does urbanization reflect a change in habitat? If so over what time scale? Or is this variable reflective of the level of urbanization at the time of analysis? Provide supporting evidence or documentation characterizing urbanization as a change.*

**Response:** Yes, the urbanization reflects a change in habit. This happens gradually over time, but with dramatic changes after 1970 -1980 (R2 Fig – R5 Fig) ^1^. Urbanization concerns encroachment on natural environments or exploitation of previously unsettled geographic areas. It has not only brought about alterations of the ecological system and species assemblage, but also contributed to closer contact between humans and domesticated/wild animals as well as vectors, resulting in the increased chance of spill-over ^2,3^. We added evidence on line 350.

Ref

1. Chini LP, Hurtt GC, Frolking S. Harmonized Global Land Use for Years 1500 -2100, V1: ORNL Distributed Active Archive Center, 2014.

2. Mackey TK, Liang BA, Cuomo R, Hafen R, Brouwer KC, Lee DE. Emerging and reemerging neglected tropical diseases: a review of key characteristics, risk factors, and the policy and innovation environment. Clinical microbiology reviews. 2014;27(4):949-79.

3. Hassell JM, Begon M, Ward MJ, Fevre EM. Urbanization and Disease Emergence: Dynamics at the Wildlife-Livestock-Human Interface. Trends in ecology & evolution. 2017;32(1):55-67.

**R2 Fig**. Distribution of urbanized land over time

**R3 Fig**. Distribution of growth of urbanized land area over time

**R4 Fig**. Distribution of urbanized of cropland over time

**R5 Fig**. Distribution of urbanized of secondary land over time

*8. Line 347 – the effect of biodiversity on virus emergence has been debated and this finding is minimally discussed here even though this is one of the effects that could be causally related to virus discovery in humans. Could the authors add more discussion of the relationship between biodiversity and virus discovery – examine this by potentially causal mechanisms and infer what this means for future trends?*

**Response:** We have added more discussion of the relationship between biodiversity and virus discovery on lines 368-374.

*9. The conclusion could be more forward looking, rather than a restatement of main findings already discussed*

**Response:** We have improved our conclusion on lines 408-417 in the revised version.

*Minor editorial suggestions*

*1. Line 31: is = are*

**Response:** We have changed ‘is’ to ‘are’ on line 31 (now on line 32) and on line 33.

*2. Line 89: Expand literature review methods description.*

**Response**: In the methods overview, we aimed to provide the brief introduction on the workflow of the paper, not extending any details. But again, this study was based on the published data from our group. To avoid repetition and redundancy, we only mentioned on line 110 that ‘Search terms, databases searched, and inclusion or exclusion criteria for data collection was provided in our previous paper’

*3.* Results: start this section with a description of the major findings, not an immediate reference to a figure in the first word of the first sentence.

**Response**: We have moved the reference of the figure after the main findings on line 214.

*4.* Line 331: rephrase, missing a verb

**Response**: We have rephrased the sentence on line 331 (now on line 350).

*5.* Line 359: awkward sentence, especially as start of new paragraph – what were other limitations mentioned?

**Response:** We mentioned our first limitation on line 379 i.e. ‘this suggests that our model is missing explanatory factors (positive or negative) relevant to these regions’. We agree it looks awkward at the start of a new paragraph so we repeat the first limitation again on line 359 (now on line 385).

*6.* Use of the term ‘species’ in figures and legends should be rephrased as ‘virus species’.

**Response:** We have changed ‘species’ to ‘virus species’ in Fig 1.

*7.* Figure 1: Legend should provide more detail and clarity on data points (to replace “occurrences” and define “centroids”. The 1B figure should have a descriptive y axis label.

**Response**: We extended the legend for Fig 1, replaced occurrence with discovery, and defined centroids. We also have changed the y axis label to ‘cumulative virus species count’ for Fig 1 (B).

*8.* Figure 5: revise to have a title and descriptive legend.

**Response:** We have added a descriptive legend for Fig 5.

*9.* SI Fig. this methods overview is not especially helpful in understanding approach or methods.

**Response:** We have removed workflow figure from our revised version.

*10.* S2 Fig needs a more descriptive legend. The bar plots overlap the numbers and are difficult to discern. Improve this graphic for readability and quality.

**Response:** We have removed S2 Fig from our revised version because it didn’t add much valuable information in comparison to Fig 1 and the S1 video.

*11.* S4 Fig, S7 Fig, S9 Fig, S10 Fig; units are unclear for some plots.

**Response:** We have added units for all explanatory factors in S4 Fig (now S2 Fig), S10 Fig (now S4 Fig), and S5 Fig (now S5 Fig). S7 Fig to S9 Fig have been removed from our revised version.

*12.* S12 Fig – the units of measure are not clear for many plots. Colors on maps do not always match the range shown on the color bar. The legend does not seem to match this figure; expand description of what is being shown in the maps.

**Response:** We have added the unit for each explanatory factor above the corresponding plot. Many variables followed a skewed distribution and we have done the log transformation to allow for a balanced color bar range. There are two remaining variables - Population change (counts) and GDP growth (PPP, billion US$2005/yr)-that we cannot do log transformation because of negative values, but we have adjusted the cut-off values to get every color be visible on the map.

We have expanded the legend of S12 Fig (now S6 Fig) on lines 586-589 to get it well matched with the figure.
